# Living in darkness: Exploring adaptation of *Proteus anguinus* in 3 dimensions by X-ray imaging

**DOI:** 10.1093/gigascience/giac030

**Published:** 2022-04-05

**Authors:** Markéta Tesařová, Lucia Mancini, Edgardo Mauri, Gregor Aljančič, Magdalena Năpăruş-Aljančič, Rok Kostanjšek, Lilijana Bizjak Mali, Tomáš Zikmund, Markéta Kaucká, Federica Papi, Jana Goyens, Anass Bouchnita, Andreas Hellander, Igor Adameyko, Jozef Kaiser

**Affiliations:** Central European Institute of Technology, Brno University of Technology, Purkyňova 123, Brno, 61200, Czech Republic; Elettra-Sincrotrone Trieste S.C.p.A., S.S. 14 - km 163,5 in Area Science Park, Basovizza, Trieste, 34149, Italy; Speleovivarium Erwin Pichl, Adriatic Speleology Society, Via Guido Reni, 2/C, Trieste, 34123, Italy; Institute Tular Cave Laboratory, Oldhamska 8a, Kranj, 4000, Slovenia; Institute Tular Cave Laboratory, Oldhamska 8a, Kranj, 4000, Slovenia; Research Centre of the Slovenian Academy of Sciences and Arts: Karst Research Institute, Titov trg 2, Postojna, 6230, Slovenia; University of Ljubljana, Biotechnical Faculty, Department of Biology, Večna pot 111, Ljubljana, 1000, Slovenia; University of Ljubljana, Biotechnical Faculty, Department of Biology, Večna pot 111, Ljubljana, 1000, Slovenia; Central European Institute of Technology, Brno University of Technology, Purkyňova 123, Brno, 61200, Czech Republic; Max Planck Institute for Evolutionary Biology, August-Thienemann-Str. 2, Plon, 24306, Germany; Speleovivarium Erwin Pichl, Adriatic Speleology Society, Via Guido Reni, 2/C, Trieste, 34123, Italy; Laboratory of Functional Morphology, University of Antwerp, Universiteitsplein 1, Wilrijk, 2610, Belgium; Department of Information Technology, Uppsala University, Box 337, Uppsala, 755 01, Sweden; Department of Integrative Biology, University of Texas at Austin, Austin, 78712, Texas, USA; Department of Information Technology, Uppsala University, Box 337, Uppsala, 755 01, Sweden; Medical University of Vienna, Center for Brain Research, Department of Neuroimmunology, Spitalgasse 4, 1090 Vienna, Austria; Karolinska Institutet, Department of Physiology and Pharmacology, Solnavagen 9, 17165 Solna, Sweden; Central European Institute of Technology, Brno University of Technology, Purkyňova 123, Brno, 61200, Czech Republic

**Keywords:** Proteus anguinus, Ambystoma mexicanum, olm, axolotl, X-ray microCT, microtomography, salamander, cave animal, subterranean adaptations

## Abstract

**Background:**

Lightless caves can harbour a wide range of living organisms. Cave animals have evolved a set of morphological, physiological, and behavioural adaptations known as troglomorphisms, enabling their survival in the perpetual darkness, narrow temperature and humidity ranges, and nutrient scarcity of the subterranean environment. In this study, we focused on adaptations of skull shape and sensory systems in the blind cave salamander, *Proteus anguinus*, also known as olm or simply proteus—the largest cave tetrapod and the only European amphibian living exclusively in subterranean environments. This extraordinary amphibian compensates for the loss of sight by enhanced non-visual sensory systems including mechanoreceptors, electroreceptors, and chemoreceptors. We compared developmental stages of *P. anguinus* with *Ambystoma mexicanum*, also known as axolotl, to make an exemplary comparison between cave- and surface-dwelling paedomorphic salamanders.

**Findings:**

We used contrast-enhanced X-ray computed microtomography for the 3D segmentation of the soft tissues in the head of *P. anguinus* and *A. mexicanum*. Sensory organs were visualized to elucidate how the animal is adapted to living in complete darkness. X-ray microCT datasets were provided along with 3D models for larval, juvenile, and adult specimens, showing the cartilage of the chondrocranium and the position, shape, and size of the brain, eyes, and olfactory epithelium.

**Conclusions:**

*P. anguinus* still keeps some of its secrets. Our high-resolution X-ray microCT scans together with 3D models of the anatomical structures in the head may help to elucidate the nature and origin of the mechanisms behind its adaptations to the subterranean environment, which led to a series of troglomorphisms.

## Data Description

### Context

*Proteus anguinus*, also known as the olm or simply proteus, has attracted the attention of scientists and animal traders for centuries. *P. anguinus* is an apex predator of the karst underground waters. Its presence indicates the stability of food chains in the subterranean ecosystem. Its geographic distribution is limited to the Dinaric Karst; it ranges from the Gulf of Trieste in Italy, through the southern half of Slovenia, coastal mainland of Croatia and parts of Bosnia and Hercegovina, as far as adjacent parts of Montenegro. With its extremely fragmented and limited habitat, *P. anguinus* is particularly vulnerable to pollution of groundwater. Some populations have been locally destroyed or endangered by pollution or habitat destruction [1, 2]. *P. anguinus* is an important object of research from at least 2 perspectives. First, *P. anguinus* played a historical role during the formation of modern science from the 17th to 19th century, puzzling the minds of most prominent early naturalists, from Valvasor to Linnaeus, Scopoli, Cuvier, and Humboldt, and from Lamarck to Darwin and Goethe. Second, *P. anguinus* has potential to answer the questions of the science of today and the future (e.g., regeneration, cave-related adaptations, enormous genome, and conservation of subterranean biodiversity) [3].

*P. anguinus* was first mentioned by Janez Vajkard Valvasor in 1689. Its scientific name *Proteus anguinus* was given by Josephus Nicolaus Laurenti in 1768, and thus *P. anguinus* became the first taxonomically described cave animal in the world [4]. In the past 2 centuries, more detailed research has been performed on *P. anguinus*. Two of the central figures of the early *P. anguinus* research were Žiga Zois, a naturalist from Ljubljana, who first described its behaviour and conducted the earliest physiological and ecological observations. Then, Karl von Schreibers, a Viennese zoologist, was the first to explore the anatomy of *P. anguinus* [5]. This mysterious animal, which retains larval features at the adult stage, started to interest the scientific community in the early 19th century, with a focus on its secretive mode of reproduction. In 1859, *P. anguinus* served as an example of blind cave animals in the famous monograph *On the Origin of Species*, where Charles Darwin attributed the reduction of eyes wholly to their disuse in darkness [6]. *P. anguinus* also became a model species in classical studies of comparative anatomy of the late 19th century. In the 20th century, more systematic research was enabled by overcoming the inaccessibility of *P. anguinus*'s subterranean habitat in captivity of cave laboratories worldwide, including France, Slovenia, and Germany; the studies focused on cave-related physiology, ecology, behaviour, and molecular phylogeny [3]. Fortunately, the attention to *P. anguinus* and its habitat has gradually received an important cultural and conservation attitude (1951 protected species in Slovenia, 1971 Ramsar convention, 1979 Bern convention, and 1992 EU Habitat Directive). The year 2019 represented another milestone in the research of *P. anguinus*. The public presentation of the *P. anguinus* genome sequencing project [7] aimed to decipher the *P. anguinus* genetic information coded in its genome, which is ∼15 times larger than the human genome.

Obligate cave-dwellers often result in a set of specific morphological, physiological, and behavioural traits (i.e., troglomorphism). Compared with their epigean relatives, cave-dwelling animals may have phylogenetically retained older sensory properties, improved them, or acquired new ones, enabling their survival in dark habitats [8]. *P. anguinus* evolved a range of adaptations such as the loss of pigmentation, slow metabolism, and capability of surviving extreme starvation. Moreover, the loss of eyesight is compensated by other specialized senses that enable navigation in complete darkness. The mode of cave life and other biological peculiarities of *P. anguinus* and other troglobionts evoke the potential role of underwater audio-, mechano-, electro-, and magnetoreception [9, 10].

Specimens of *P. anguinus* are valuable study material because they are protected and vulnerable. Our knowledge of *P. anguinus* larval and juvenile stages is still scarce. For these reasons, we aimed to use *P. anguinus* specimens from existing collections to avoid collecting them from nature. These animals died by natural causes and their bodies were preserved. We accessed the collection, applied non-destructive staining and a non-destructive imaging method (X-ray computed microtomography [microCT]), and then returned the specimens back to the collection. In this way, we obtained high-quality and high-resolution 3D data without needing to kill any animal.

X-ray microCT has become a powerful method in developmental biology for exploring morphological changes in 3 dimensions. Geometric morphometrics based on X-ray microCT has already been used previously for an exploratory analysis of the morphology of the cranial osteology in the white and black subspecies of *P. anguinus* [11, 12]. However, without contrast-enhanced techniques, less dense tissues such as sensory organs would be unrecognizable or inaccurately captured owing to insufficient contrast for detailed 3D analysis. In our study, we use staining of soft tissues by phosphotungstic acid (PTA) or iodine to visualize soft tissues in the head of *P. anguinus*. For the first time, we look at volumetric internal structures by using a non-destructive imaging technique and we show the sensory organ with a high spatial and contrast resolution.

*P. anguinus* has been an important object of study in the history of international nature research, intriguing scientists. Thanks to studies in the past 300 years [5], the mysteries of this cave amphibian are slowly being unravelled. However, *P. anguinus* still leaves gaps in our knowledge about its ecology, evolution, and physiology. Our high-resolution microCT scans, together with 3D models of anatomical structures in the head, could help to elucidate the nature and origin of the mechanisms behind its adaptations to the subterranean lightless environment, which led to the acquisition of the troglomorphic features.

### Methods

#### Sample preparation

Approval for the capture, handling, maintenance, and breeding in captivity of the animals used in the study was granted by the Ministry of the Environment and Spatial Planning of the Republic of Slovenia, Slovenian Agency for the Environment (Permits Nos. 35701-36/01, 35601-95/2009-4, and 35601-132/2014-4), by the Italian Republic, Friuli Venezia Giulia Region (Permit Nos. 4105/6MU4/95/04/12), and by the Italian Republic, Ministry of Ecological Transition (Permits Nos. 3006/015590-93, 39/04). No animals were killed for this study. Our experimental plan involved scanning *P. anguinus* and the axolotl *Ambystoma mexicanum* samples at different stages to study various parts of the skull and sensory organs.

All 5 *P. anguinus* specimens (NCBI:txid221568) were stored in 75% ethanol. Specimens of *A. mexicanum* (NCBI:txid8296) had been reared at the Speleovivarium Erwin Pichl (Italy) since 2004 and included a 5-year-old adult female (died in 2009) with a total length of 194 mm, and a larva of 6 days old (died in 2012) with a total length of 11 mm. Specimens of *P. anguinus* included 1 larva, 3 weeks old (died in 2007), length 23 mm, captive breeding originating from the Postojna-Planina Cave System, from the collection of the Tular Cave Laboratory (Slovenia); 1 juvenile, 35 mm long, deceased at the collection site in spring near Metković (Croatia), from the collection of the Department of Biology, Biotechnical Faculty, University of Ljubljana (Slovenia); 1 adult, sex unknown, length 276 mm, collected in Postojna-Planina Cave System (Slovenia, 1989), died in captivity of the Speleovivarium in 1999, from the collection of the Speleovivarium Erwin Pichl (Italy).

We modified a contrast-enhancing protocol initially developed by Brian Metscher [13] that has been successfully applied on salamander tissues before [14]. Larval samples of both *P. anguinus* and *A. mexicanum*, and juvenile *P. anguinus* were stained with 1% PTA in 90% methanol for 7 weeks. The solution was exchanged with a fresh one once a week. The adult *P. anguinus* and *A. mexicanum* specimens were stained with 2% iodine (instead of PTA) in 90% methanol for 6 weeks to ensure that the contrasting agent would penetrate the entire sample because iodine penetrates better than PTA. Subsequently, the samples were gradually rehydrated in ethanol series (90%, 80%, 70%, and 50%), 1 day for each concentration (i.e., 4 days of rehydration). The samples were then stabled in polyamide tubes filled with 1% low-melting agarose gel to prevent sample movement during CT scan.

#### Image acquisition

The head region of the samples was scanned by using a laboratory X-ray microCT system GE Phoenix v|tome|x L 240 (Waygate Technologies / Baker Hughes Digital Solutions GmbH, Wunstorf, Germany). The system was equipped with a 180 kV/15 W maximum power nanofocus X-ray tube and a high-contrast flat-panel detector DXR250 with 2,048 × 2,048 pixels resolution and (200 × 200) μm^2^ pixel size. A total of 2,000 projections over a total scan angle of 360° were acquired with an exposure time of 900 ms per projection. Each projection was captured 3 times, and an average of the signal was used to improve the signal-to-noise ratio. The acceleration voltage of the X-ray tube was set to 60 kV and the tube current to 200 μA for larval and juvenile *P. anguinus*; 80 kV and 250 μA were used, respectively, for voltage and current for adult samples. The X-ray beams of lower energies were filtered with a 0.2-mm-thick aluminium plate for larval and with a 1-mm-thick aluminium plate for adult samples. The voxel sizes of the reconstructed data were as follows: 3.5 μm for juvenile *P. anguinus*, 5.8 μm for larval *P. anguinus*, 25 μm for adult *P. anguinus*, 2.8 μm for larval *A. mexicanum*, and 27.5 μm for adult *A. mexicanum*.

#### Tomographic data processing

The tomographic reconstruction was performed using GE phoenix datos|x 2.0 software (Waygate Technologies / Baker Hughes Digital Solutions GmbH, Wunstorf, Germany) (Phoenix Datos|x 2.0, RRID:SCR_017996). A segmentation procedure was then applied to reconstructed slices. The Avizo 7.1 (Thermo Fisher Scientific, Waltham, MA, USA) (Avizo 3D Software, RRID:SCR_014431) image processing software was used for semi-automatic segmentation [15–17] of structures in the head. To reduce the load of the 3D segmentation volume, every third slice was manually segmented, and the rest was calculated by linear interpolation between manually segmented slices [17]. We converted the semi-manually segmented models into polygonal meshes and imported this in VG Studio MAX 2.2 software (Volume Graphics GmbH, Heidelberg, Germany) (VG Studio MAX, RRID:SCR_017997) for 3D visualizations.

### Data validation and quality control

**Figure 1:**
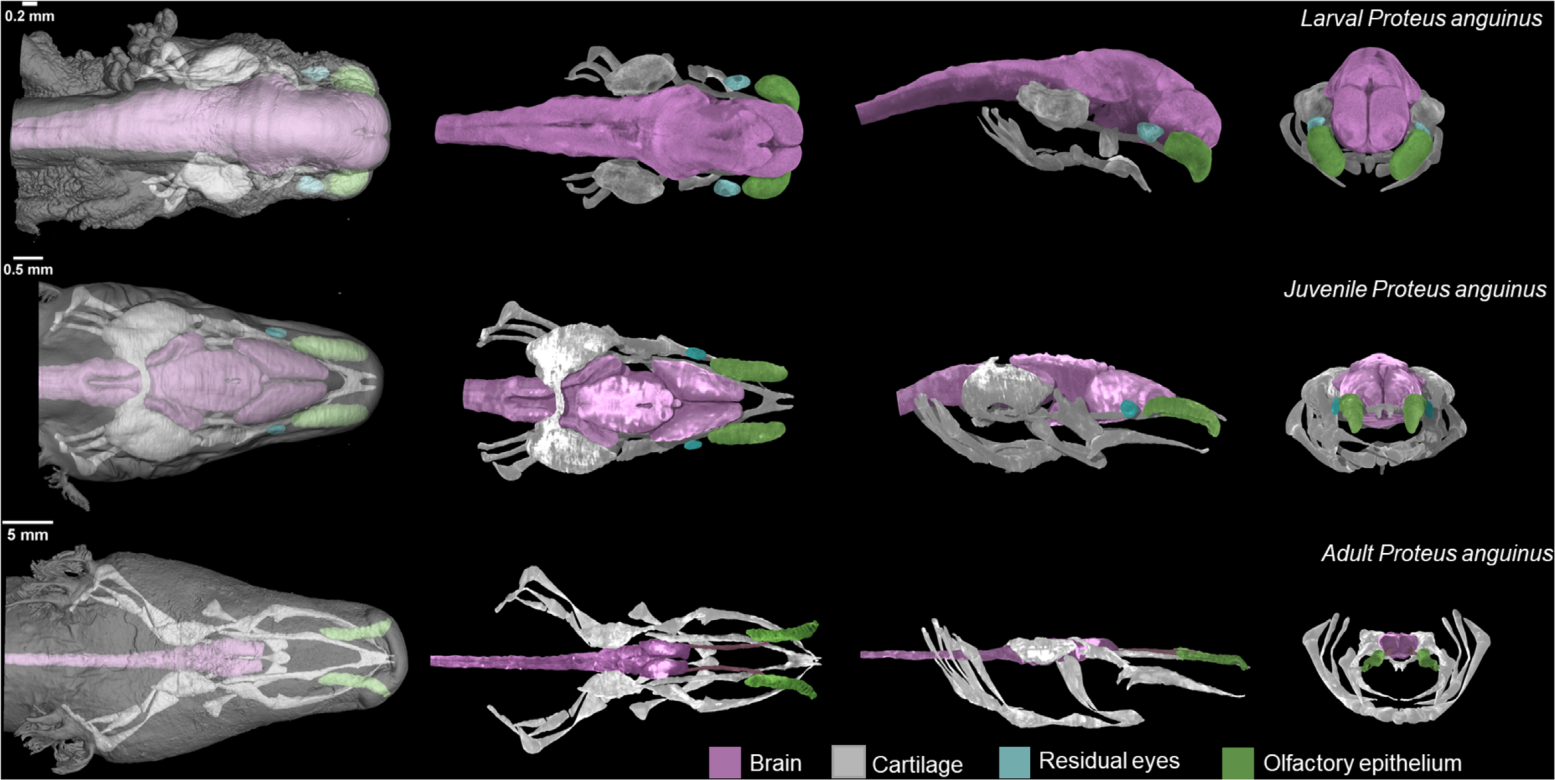
3D reconstructions of *P. anguinus* head based on X-ray microCT data. Larva (top), juvenile (middle), and adult (bottom) *P. anguinus*. Images in the first column show semi-transparent 3D renderings of the head with skin in dorsal view. Dorsal, lateral, and frontal views of the segmented and color-coded internal soft structures are shown in the second to the fourth columns.

By contrast-enhanced X-ray microCT scan, we were able to visualize the internal structures of the *P. anguinus* head. Figure 1 shows the manually segmented cartilaginous chondrocranium, as well as the position and the shape of the brain, the remnant eyes, and the olfactory epithelium. A considerable portion of the cranial skeleton in the adult *P. anguinus* specimen remains cartilaginous.

**Figure 2:**
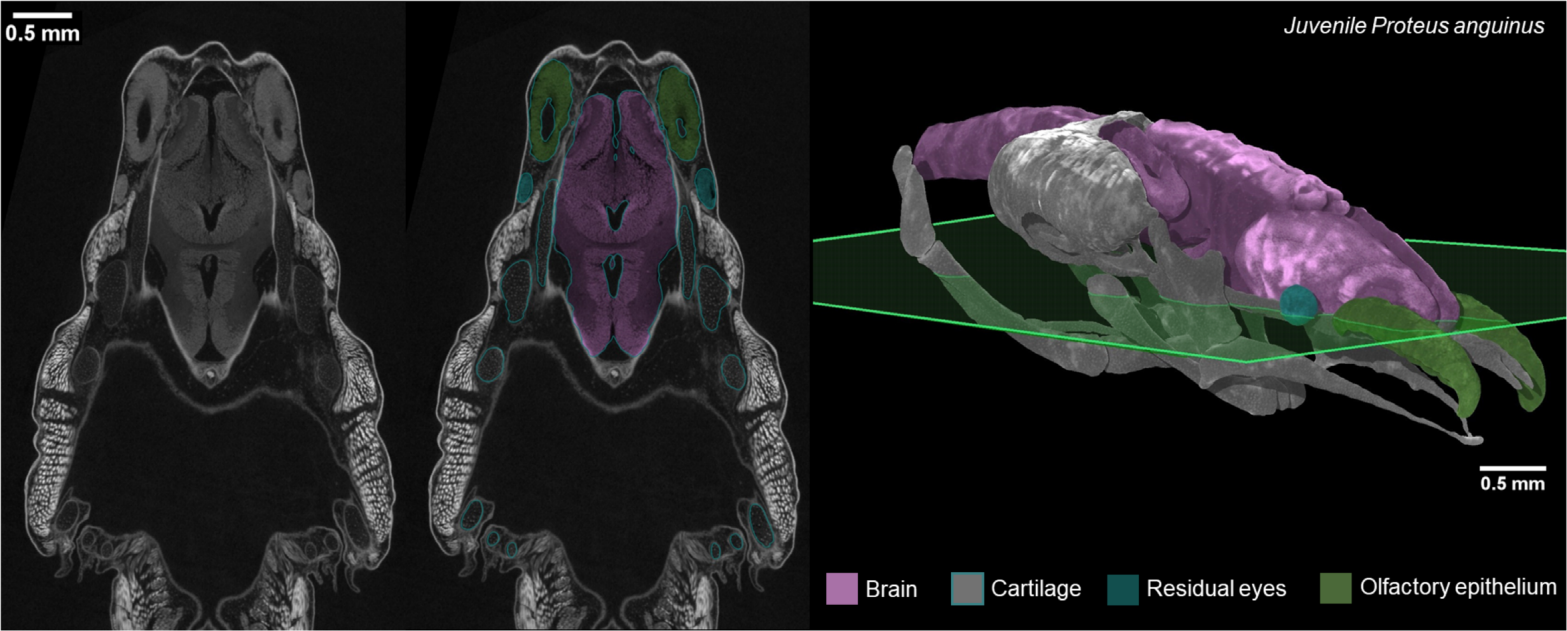
Accuracy validation of semi-automatic segmentations of X-ray microCT data in juvenile *P. anguinus*. Raw CT image (left) and the corresponding segmented image (middle) through the transverse plane (green) of the head of a juvenile *P. anguinus* (right).

A validation of the semi-automatic segmentation procedure is presented in Fig. 2. The 3D models were created by an operator based on the grey-scale value contrast and the shape of the structures. The detailed procedure is described in the Methods section and followed our previous study [17].

**Figure 3:**
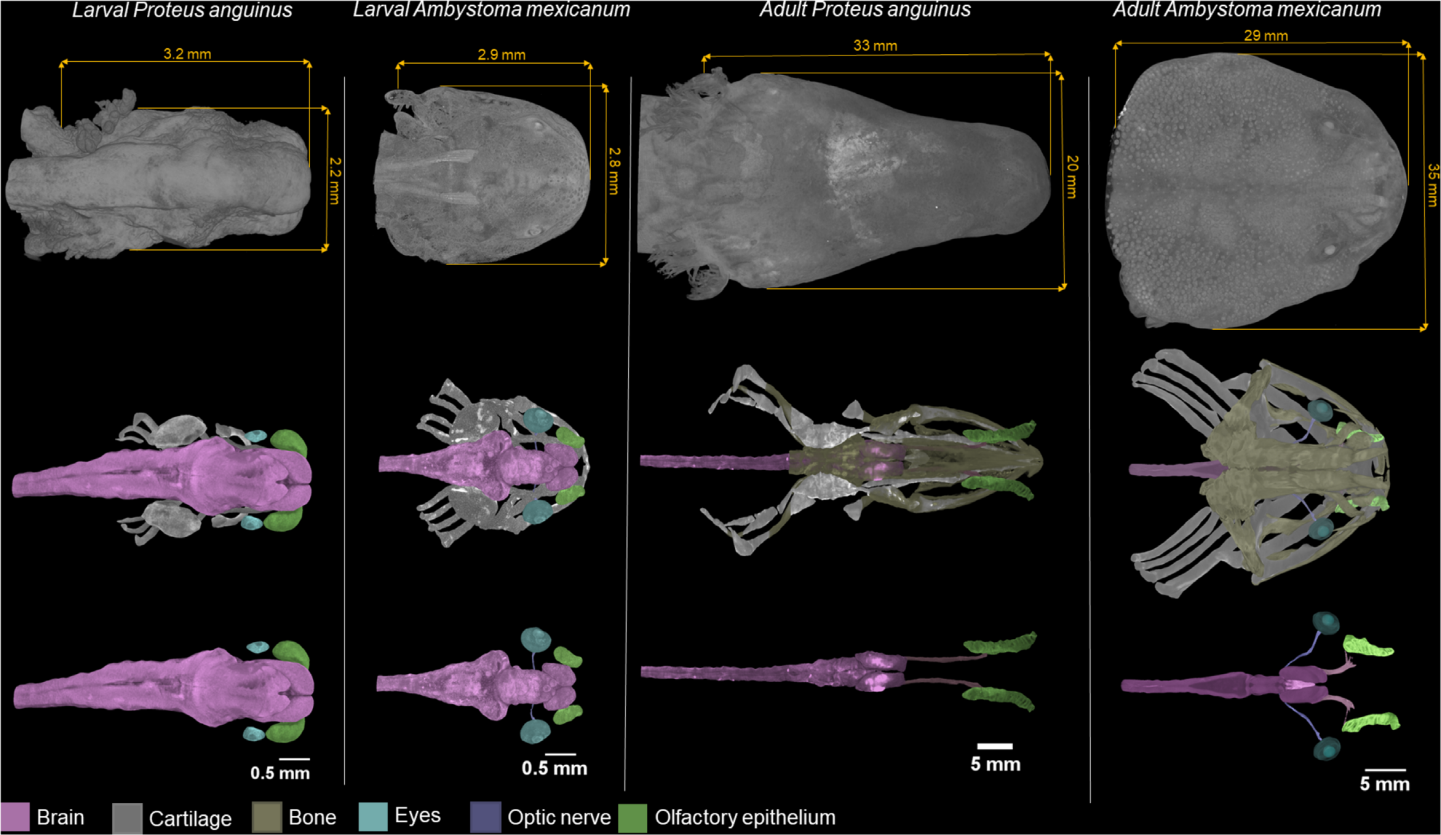
Comparison of the head and internal soft-tissue anatomy of the cave-dwelling *P. anguinus* with *A. mexicanum* in larval and adult specimens. Images in the first row show 3D renderings of the head with skin in dorsal view. The second row shows color-coded segmented brain, cartilage, bones, and eyes with optic nerve and olfactory epithelium. The third row shows these structures without bone and cartilage for better clarity.

In Fig. 3, we compare the microCT datasets and segmentations of the internal head structures of the larval and adult specimens of both *P. anguinus* and *A. mexicanum*. The head of the troglobiotic *P. anguinus* is narrower and more elongated in comparison with the epigean *A. mexicanum*, which lives in open surface bodies of water but not underwater caves. Our 3D segmentations show remnant eyes at larval and juvenile stage *P. anguinus*, but no remnant of eyes was noticed in the adult specimen. The progressive degeneration of the eye in the development of *P. anguinus* may lead to the apparent disappearance of the eye in the adult animal [18]. By contrast, 3D models of *A. mexicanum* clearly show the eyes, together with an optic nerve that leads to the brain, in both the larval and adult stages. However, the absence of remnant eyes and optic nerves in our data could be caused by low contrast of these structures in microCT data because the segmentation was done manually by the operator and based on their grey-scale values and their shape.

### Reuse potential

#### Museum-type documentation of rare and endangered species

The presented datasets give insight not only into the developmental biology of *P. anguinus*. Together with 3D datasets of *A. mexicanum*, they are important study materials for the conservation efforts to preserve these endangered amphibians. According to the global assessment of the International Union for Conservation of Nature (IUCN), 43% of amphibian species are in decline while 32% are threatened with extinction [19]; *P. anguinus* is currently classified as vulnerable. The X-ray microCT method enables anatomical studies without damaging the morphology of the specimens and is therefore exceptionally appropriate for studying endangered species with a limited amount of available specimens. Semi-automatically segmented images and the extracted 3D models could also be taken as an input into a machine learning algorithm. The field of image processing is becoming dominated by deep learning algorithms and convolutional neural networks [20]. Creating an online database could also be beneficial for student studies and distance learning. Especially, last year showed the importance of easy access to online study materials because of Covid-19 restrictions.

#### Perspectives: Cellular resolution

**Figure 4:**
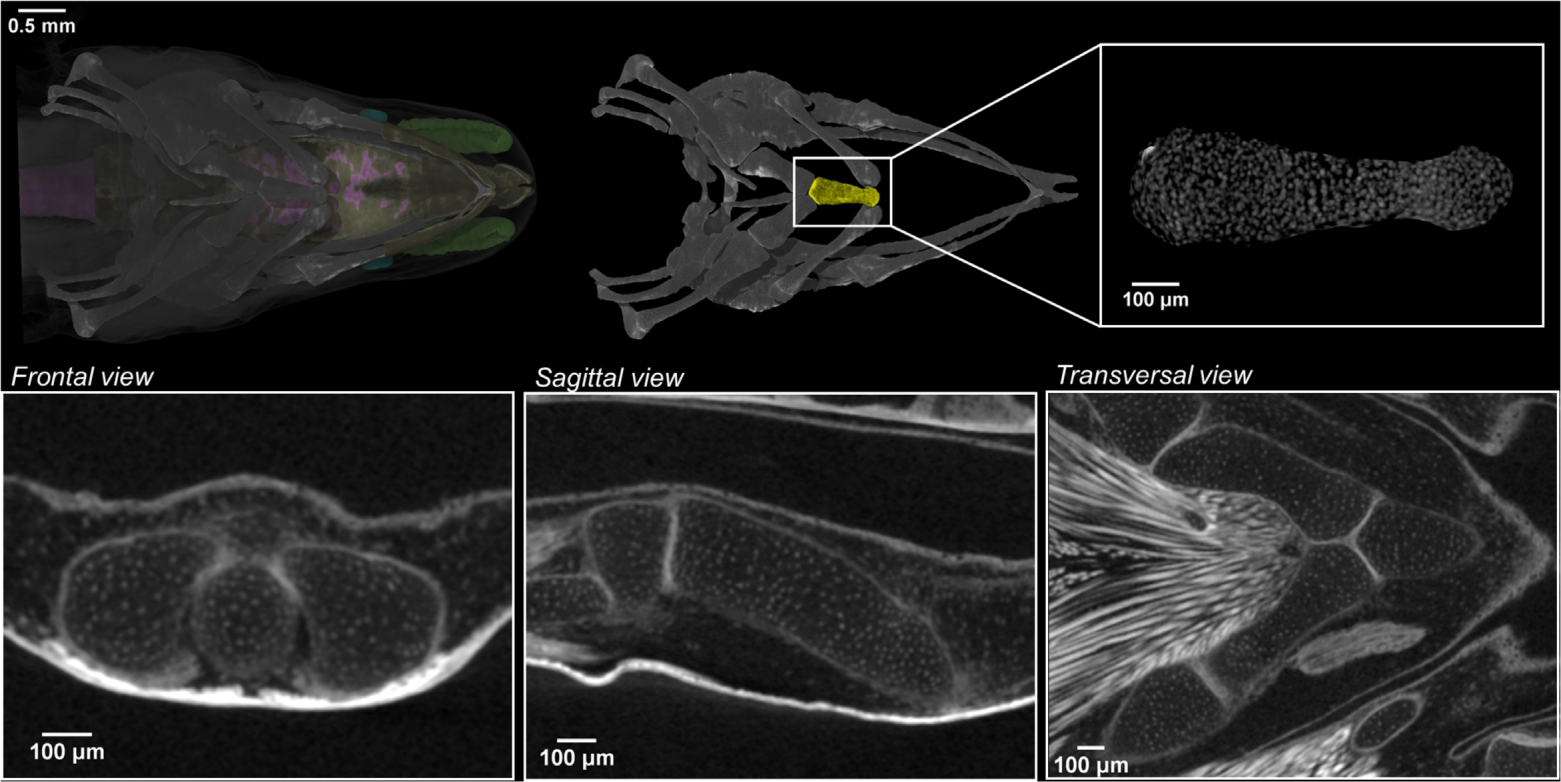
Images at near cellular-level resolution showing the cartilaginous elements in juvenile *P. anguinus* obtained by microCT. The white dots represent cell nuclei. 3D detail of the cartilaginous first basibranchial element of the hyobranchial apparatus in ventral view (yellow; top row) with 3 orthogonal CT slices along the frontal, sagittal, and transverse planes (second row).

Using microCT scan with a conventional X-ray source, we obtained data of excellent quality that depict single cells in the cartilaginous elements (Fig. 4). Despite the fact that the cells can be visually detected, their automatic segmentation and quantification is challenging. The potential of X-ray microCT imaging with synchrotron sources for the study of 3D cell distribution was demonstrated in our previous study on salamander limbs [12], and the potential for biomedical applications was shown before [21, 22]. The data with cellular resolution can be used as the input for the study of polarization of cells in the extracellular matrix in salamander limbs or for mathematical modelling of joint formation [14].

#### Research outlook

**Figure 5:**
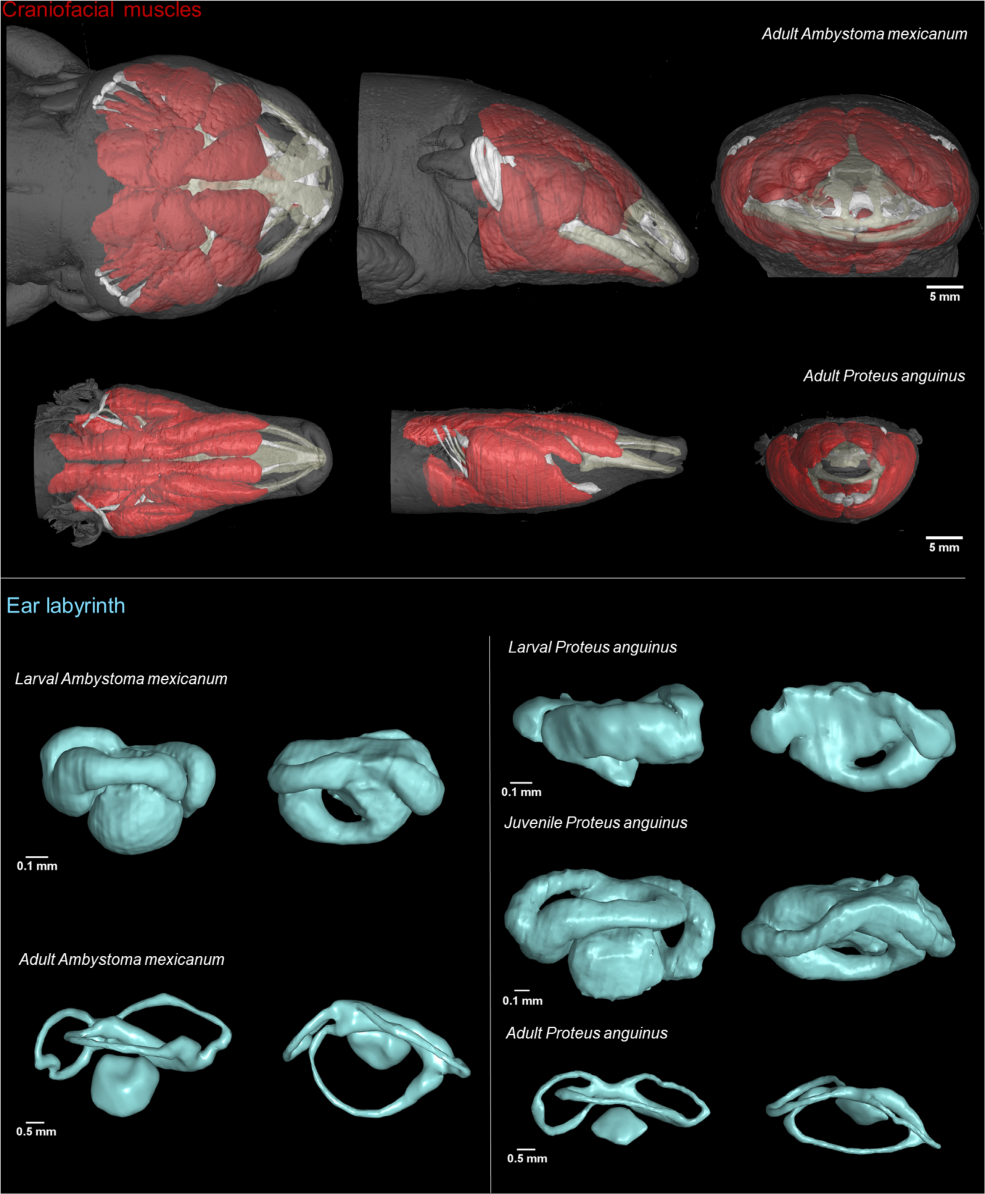
Potential use of contrast-enhanced X-ray microCT data. Segmentation of craniofacial muscles and ear labyrinth in *A. mexicanum* and *P. anguinus*.

The morphology of the cranium carries important information related to mechanics involved in feeding, as well as competitive, reproductive, and anti-predatory behaviour. Even small differences in cranial skeleton may have important biomechanical and ecological implications [23]. The most detailed descriptions of the *P. anguinus* skull are those of Dolivo-Dobrovolsky [24, 25], Ivanović et al. [11], Papi et al. [12], and Bizjak Mali and Sket [26]. MicroCT contrast-enhanced data included overall 3D information and a further segmentation. Working on the provided data can be taken as an input for various type of analyses (Fig. 5). The visualization of muscles of the upper and lower jaw could be used for biting analyses that may shed light on the predatory abilities of *P. anguinus*.

In general, our datasets will help to investigate how evolutionary changes in the shape of the head and its integrated musculoskeletal apparatus, nervous system, and sensory organs would help for troglomorphic adaptation. Because *P. anguinus* is a cave-dwelling animal, the craniofacial design underwent a number of cave-related evolutionary adaptations, acquiring protracted and longitudinally elongated skeletal elements, in comparison with the epigean *A. mexicanum*, as revealed by comparative microCT scanning analysis and in-depth 3D analysis. Indeed, when compared to *P. anguinus, A. mexicanum* exhibits a wider skull with a massive jaw. Elongated skull, body, and limbs are common features of troglomorphism for organisms inhabiting subterranean habitats [27, 28]. Only 14 cave-obligate species are known (all plethodontid and proteid salamanders), but this number is likely to be an underestimate because of cryptic species [29]. In this regard, the results of our study provide insight into evolutionary trends in adaptive morphological traits as a result of convergent evolution across phyla. Even without evolutionary comparison across phyla, our results enable further studies on evolution of the skeletal parts in the adaptive landscapes individually for closely related species.

Not only evolutionary questions but also developmental patterns can be tackled with these data. For instance, to investigate whether the elongated skull shape develops evenly with the growth of the animal, we investigated the larval *P. anguinus*. The larva of *P. anguinus*, although small, revealed its miniaturized protracted chondrocrania with similar spatial proportions to the adult form. Thus, the elongated body and stretched cranium may indicate possible benefits at any phase of development. Such comparative developmental and growth-related studies open up new opportunities to look into the dynamics of skeletal shape development in relation to the specifics of the environment.

However, when it comes to the adaptations in sensory organs, the 3D analysis of the head revealed major differences in visual and olfactory systems of *P. anguinus* and *A. mexicanum*. First, *P. anguinus* is blind, a typical troglomorphic trait that is explained by the troglobiotic way of life. The eye development in *P. anguinus* larva begins as in other amphibians. The regression of the almost normally formed eyes starts soon after hatching and gradually leads to a considerable reduction of the eyes in adult *P. anguinus* [18], while surface-dwelling salamanders develop fully functional eyes. The morphological comparisons of the *P. anguinus* developmental stages with their gradual reduction of eyes may improve our knowledge on the mechanisms of eye degeneration in *P. anguinus* and possibly other similar blind cave-dwellers. Moreover, the eyeballs develop in a complex conjunction with muscular apparatus [30] and optical nerve. Here, in *P. anguinus*, it is possible to question how the induction and degeneration of auxiliary tissues is achieved during the degradation of the pre-shaped eyeballs.

The animals with eye regression are generally known to inhabit caves; therefore vision is nearly useless to them. The blind Mexican cave fish (*Astyanax mexicanus*) is one of the popular model systems to study the loss of vision and developmental arrest of the visual system [31]. However, blind fish and other model organisms, including *P. anguinus*, still have a great potential for comparison with other eyeless vertebrates [32]. Therefore, our results and datasets may enable new insights into evolutionary trends in the eye development and degeneration strategies in cave-dwellers across vertebrate clades.

Olfaction plays an important role in the life of salamanders [8]. The analysis of 3D-rendered olfactory organs revealed striking differences between the surface-dwelling *A. mexicanum* and the cave-dwelling *P. anguinus*. Elongated and tube-shaped olfactory cavities in *P. anguinus* likely emerge as another adaptation to the cave environment, where enhanced olfaction capabilities pose an advantage in the absence of visual signals [33]. Comparing to *A. mexicanum*, the elongated olfactory cavities of *P. anguinus* might enable a higher dynamic range of sensitivity, owing to a more efficient longitudinal diffusion of signals upon entry via the nostrils. In line with this, the olfactory nerves of *P. anguinus* are also considerably elongated, which is explained by a rather long rostral part of the skull in *P. anguinus*. Therefore, studies of evolutionary divergence of the olfactory system and its sensitivity and general design could benefit from the deposited datasets.

Finally, salamanders represent a well-established model system for the research of regeneration, and the fundamental principles of multi-tissue regeneration have already been revealed [34]. Regeneration of *P. anguinus* has been described previously, yet the provided data offer an important insight into the evolutionary differences in regeneration among salamanders with fundamentally different lifestyles.

## Data Availability

The datasets underlying this article are available in the *GigaScience* Database repository [35]. We provide reconstructed slices as DICOM image stacks and segmented structures in STL format for the head region of 3 specimens (1 larva, 1 juvenile, and 1 adult) of *P. anguinus* and 2 specimens (1 larva and 1 adult) of *A. mexicanum*. For segmented structures, we also provide segmented masks as DICOM image stacks—1 stack for each structure. The folders are structured so that each folder represents 1 sample containing a folder with DICOM stack, a folder with STL files, and a folder with segmented masks. The DICOM image stacks can be opened in any image viewer supporting this format; we recommend ImageJ for viewing the data [36]. To explore datasets in 3 dimensions, specialized free viewers are available—Drishti [37], DragonFly (Object Research Systems [ORS], Inc., Montreal, QC, Canada), or others. For analysis and further segmentation, we recommend the use of ITK-SNAP [38] or commercial software, e.g., Avizo or VG Studio MAX. A detailed description and a manual for segmentation of biological data can be found in our previous works [17, 39]. The STL files can be also explored in 3D mesh viewers: popular free open-source software, e.g., MeshLab [40] or Blender [41], as well as in the sketchfab repository [42].

## Abbreviations

DICOM: Digital Imaging and Communications in Medicine; microCT: X-ray computed microtomography; PTA: phosphotungstic acid; STL: Standard Triangle Language.

## Competing Interests

The authors declare that they have no competing interests.

## Funding

We acknowledge CzechNanoLab Research Infrastructure supported by MEYS CR (LM2018110). M.T. acknowledges grant CEITEC VUT-J-21-7145, the Brno City Municipality as a Brno Ph.D. Talent Scholarship Holder and Martina Roeselova Memorial Fellowship. J.G. was funded the Fonds Wetenschappelijk Onderzoek–Vlaanderen by a postdoctoral fellowship (Grant No. 12R5118N).

## Authors' Contributions

M.T.: Writing—original draft, Visualization; L.M.: Conceptualization, Methodology; E.M.: Investigation, Writing—original draft; G.A.: Investigation, Writing—original draft; M.N.A.: Investigation, Writing—original draft; R.K.: Validation, Writing—review & editing; L.B-M.: Validation; T.Z.: Project administration; M.K.: Methodology; Writing—review & editing; F.P.: Methodology; J.G.: Data curation, Writing—review & editing; A.B.: Data curation; A.H.: Project administration; I.A.: Conceptualization, Project administration; J.K.: Funding acquisition​, Supervision.

## Supplementary Material

giac030_GIGA-D-21-00259_Original_Submission

giac030_GIGA-D-21-00259_Revision_1

giac030_Response_to_Reviewer_Comments_Revision_1

giac030_Reviewer_1_Report_Original_Submission Jia Jia -- 10/8/2021 Reviewed

giac030_Reviewer_2_Report_Original_Submission Claudio Polisseni -- 10/11/2021 Reviewed

giac030_Reviewer_2_Report_Revision_1 Claudio Polisseni -- 2/7/2022 Reviewed

giac030_Reviewer_3_Report_Original_Submission Chris Armit -- 10/11/2021 Reviewed

giac030_Reviewer_3_Report_Revision_1 Chris Armit -- 2/1/2022 Reviewed

giac030_Reviewer_4_Report_Original_Submission Alexander Kupfer -- 10/19/2021 Reviewed
