# Supplementary material for: Living in darkness: Exploring adaptation of Proteus anguinus in 3 dimensions by X-ray imaging
Source: Gigascience. 2022 Apr 5;11:giac030. doi: 10.1093/gigascience/giac030 (PMC8982192; doi:10.1093/gigascience/giac030)

GigaScience

Living in darkness: Exploring adaptation of Proteus anguinus in 3D by X-ray imaging

--Manuscript Draft--

**Manuscript Number:**	GIGA-D-21-00259
**Full Title:**	Living in darkness: Exploring adaptation of Proteus anguinus in 3D by X-ray imaging
**Article Type:**	Data Note
**Funding Information:**	Ministerstvo Školství, Mládeže a Tělovýchovy (LM2018110) — Prof. Jozef Kaiser<br>Vysoké Učení Technické v Brně (CEITEC VUT-J-21-7145) — Mrs. Markéta Tesařová<br>Fonds Wetenschappelijk Onderzoek (12R5118N) — Dr. Jana Goyens
**Abstract:**	Background: The lightless caves can harbour a wide range of living organisms. Cave animals developed a set of morphological, physiological and behavioural adaptations known as troglomorphisms, enabling their survival in perpetual darkness, narrow temperature and humidity ranges, and nutrient scarcity of subterranean environment. In this study, we focused on adaptations of skull shape and sensory systems in blind cave salamander, Proteus anguinus, also known as olm or simply proteus - the largest cave tetrapod and the only European amphibian living exclusively in subterranean environments. This extraordinary amphibian compensates the loss of sight by enhanced non-visual sensory systems including mechanoreceptors, electroreceptors and chemoreceptors. We compared developmental stages of Proteus anguinus with Ambystoma mexicanum, also known as axolotl, as an exemplary comparison between cave- and surface-dwelling salamanders.<br>Findings: We used contrast-enhanced X-ray computed microtomography for the 3D segmentation of the soft tissues in the head of proteus and axolotl. Sensory organs were visualised in order to help understanding how the animal is adapted to living in complete darkness. X-ray microCT datasets together with 3D models are provided for larvae, juvenile and adult salamanders, showing the cartilage of chondrocranium, the position, shape and size of the brain, eyes and olfactory epithelium.<br>Conclusions: Proteus anguinus still keeps some of its secrets to itself. Our high-resolution X-ray microCT scans together with 3D models of anatomical structures in head could help understand the nature and origin of the mechanisms behind its adaptations to the subterranean environment, which led to the set of troglomorphies.
**Corresponding Author:**	Jozef Kaiser<br><br>CZECH REPUBLIC
**Corresponding Author Secondary Information:**	
**Corresponding Author's Institution:**	
**Corresponding Author's Secondary Institution:**	
**First Author:**	Markéta Tesařová
**First Author Secondary Information:**	
**Order of Authors:**	Markéta Tesařová
	Lucia Mancini
	Edgardo Mauri
	Gregor Aljančič
	Magdalena Năpăruș-Aljančič
	Rok Kostanjšek

Powered by Editorial Manager® and ProduXion Manager® from Aries Systems Corporation

	Lilijana Biziak Mali
	Tomáš Zikmund
	Markéta Kaucká
	Federica Papi
	Jana Goyens
	Anass Bouchnita
	Andreas Hellander
	Igor Adameyko
	Jozef Kaiser
**Order of Authors Secondary Information:**	
**Additional Information:**	
**Question**	**Response**
Are you submitting this manuscript to a special series or article collection?	No
**Experimental design and statistics**<br><br>Full details of the experimental design and statistical methods used should be given in the Methods section, as detailed in our Minimum Standards Reporting Checklist. Information essential to interpreting the data presented should be made available in the figure legends.<br><br>Have you included all the information requested in your manuscript?	Yes
**Resources**<br><br>A description of all resources used, including antibodies, cell lines, animals and software tools, with enough information to allow them to be uniquely identified, should be included in the Methods section. Authors are strongly encouraged to cite Research Resource Identifiers (RRIDs) for antibodies, model organisms and tools, where possible.<br><br>Have you included the information requested as detailed in our Minimum Standards Reporting Checklist?	Yes

**Availability of data and materials**<br><br>All datasets and code on which the conclusions of the paper rely must be either included in your submission or deposited in publicly available repositories (where available and ethically appropriate), referencing such data using a unique identifier in the references and in the "Availability of Data and Materials" section of your manuscript.<br><br>Have you have met the above requirement as detailed in our Minimum Standards Reporting Checklist?	Yes

Powered by Editorial Manager® and ProduXion Manager® from Aries Systems Corporation

Main text of the manuscript
Click here to access/download;Manuscript;Proteus_Tesarova_gigascience_f

Living in darkness: Exploring adaptation of *Proteus anguinus* in 3D by X-ray imaging

Authors:

Markéta Tesařová[1], Lucia Mancini[2], Edgardo Mauri[3], Gregor Aljančič[4], Magdalena Năpăruş-Aljančič[4,5], Rok Kostanjšek[6], Lilijana Biziak Mali[6], Tomáš Zikmund[1], Markéta Kaucká[7], Federica Papi[3], Jana Goyens[8], Anass Bouchnita[9,10], Andreas Hellander[9], Igor Adameyko[11] and Jozef Kaiser[1*]

[1] Central European Institute of Technology, Brno University of Technology, Czech Republic

[2] Elettra-Sincrotrone Trieste S.C.p.A., Basovizza, Trieste, Italy

[3] Speleovivarium Erwin Pichl, Adriatic Speleology Society, Trieste, Italy

[4] Tular Cave Laboratory, Society for Cave Biology, Kranj, Slovenia

[5] Research Centre of the Slovenian Academy of Sciences and Arts: Karst Research Institute, Postojna, Slovenia

[6] University of Ljubljana, Biotechnical Faculty, Department of Biology, Ljubljana, Slovenia

[7] Max Planck Institute for Evolutionary Biology, Plon, Germany

[8] Laboratory of Functional Morphology, University of Antwerp, Belgium

[9] Department of Information Technology, Uppsala University, Sweden

[10] Department of Integrative Biology, University of Texas at Austin, USA

[11] Medical University of Vienna, Vienna, Austria

* Jozef.Kaiser@ceitec.vutbr.cz

Abstract

Background: The lightless caves can harbour a wide range of living organisms. Cave animals developed a set of morphological, physiological and behavioural adaptations known as troglomorphisms, enabling their survival in perpetual darkness, narrow temperature and humidity ranges, and nutrient scarcity of subterranean environment. In this study, we focused on adaptations of skull shape and sensory systems in blind cave salamander, *Proteus anguinus*, also known as olm or simply proteus - the largest cave tetrapod and the only European amphibian living exclusively in subterranean environments. This extraordinary amphibian compensates the loss of sight by enhanced non-visual sensory systems including mechanoreceptors, electroreceptors and chemoreceptors. We compared developmental stages of *Proteus anguinus* with *Ambystoma mexicanum,* also known as axolotl*,* as an exemplary comparison between cave- and surface-dwelling salamanders.

Findings: We used contrast-enhanced X-ray computed microtomography for the 3D segmentation of the soft tissues in the head of proteus and axolotl. Sensory organs were visualised in order to help understanding how the animal is adapted to living in complete darkness. X-ray microCT datasets together with 3D models are provided for larvae, juvenile and adult salamanders, showing the cartilage of chondrocranium, the position, shape and size of the brain, eyes and olfactory epithelium.

Conclusions: *Proteus anguinus* still keeps some of its secrets to itself. Our high-resolution X-ray microCT scans together with 3D models of anatomical structures in head could help understand the nature and origin of the mechanisms behind its adaptations to the subterranean environment, which led to the set of troglomorphies.

Keywords

Proteus anguinus, Ambystoma mexicanum, olm, axolotl, X-ray microCT, microtomography, salamander, cave animal, subterranean adaptations

Data Description

Context

Proteus anguinus, also known as olm or simply proteus has been under the attention of scientists and animal traders for centuries. Proteus is an apex predator of the karst underground waters, which presence indicates the stability of food chains and absence of pollution in subterranean ecosystem. Proteus is today an important object of research from at least two perspectives. On one side, proteus had a historical role during the formation of the modern science from 17th to 19th century, puzzling the minds of most prominent early naturalists, from Valvasor to Linnaeus, Scopoli, Cuvier and Humboldt, and from Lamarck to Darwin and Goette. On the other side, proteus carries its potential to bring answers on the questions of the science today and tomorrow (e.g., regeneration, cave-related adaptations, enormous genome, and conservation of subterranean biodiversity) [1].

The animal was first mentioned in 1689 by Janez Vajkard Valvasor. Its scientific name *Proteus anguinus* was given by Josephus Nicolaus Laurenti in 1768 and thus proteus became the first taxonomically described cave animal in the world [2]. In the last two centuries, more detailed research has been performed on proteus. Two of the central figures of the early proteus research were Ljubljana naturalist Žiga Zois, the first who described proteus behaviour, and conducted earliest physiological and ecological observations, together with Viennese zoologist Karl von Schreibers, who first described the anatomy of proteus [3]. This mysterious animal with larval features gained the interest of the scientific community from the early 19th century, focused particularly on its secretive mode of reproduction. In 1859, proteus served as an example of blind cave animals in the famous monograph *On the Origin of Species,* where Charles Darwin attributed the reduction of eyes wholly to their disuse in darkness [4]. Proteus also became a model species in classical studies of comparative anatomy of the late 19th century. In the 20th century, more systematic research became possible by overcoming the inaccessibility of its subterranean habitat in captivity of cave laboratories worldwide, including France, Slovenia and Germany, with studies on cave-related physiology, ecology, behaviour and molecular phylogeny [1]. Fortunately, attention to proteus and its habitat have gradually received an

important cultural and conservation attitude (1951 protected species in Slovenia, 1971 Ramsar convention, 1979 Bern convention and 1992 EU Habitat Directive). The year 2019 represents another milestone in the research of proteus. The public presentation of the project of *Proteus anguinus* genome sequencing [5] is aiming to decipher proteus genetic information coded in the genome, approximately 15-times larger than the human genome.

Obligate cave-dwelling often leads to a specific set of morphological, physiological and behavioural traits (i.e., troglomorphism). Compared to their epigean relatives, cave animals may have retained phylogenetically older sensorial properties, improved them, or acquired new ones, enabling their survival in dark habitats [6]. It evolved a range of adaptations such as loss of pigmentation, slow metabolic rate, and capability of extreme starvation, as well as compensation of the loss of eyesight by other specialized senses which allow it to navigate in complete darkness. The mode of cave life and other biological peculiarities of proteus and other troglobionts evoke the potential role of underwater audio-, mechano-, electro- and magnetoreception [7, 8].

The specimens of *Proteus anguinus* are most precious study material, because of a high vulnerability of this protected species, while the larval and juvenile stages are also extremely rare. For these reasons, we aimed to use proteus specimens from existing collections to avoid collecting them from nature. Natural causes of death of these animals in captivity and their body preservations related to the use of high technologies such as X-ray computed microtomography (microCT) has allowed the discovery of a large amount of high-quality data reducing at minimum the number of sacrificed specimens.

X-ray microCT has become a powerful method for exploring morphological changes in 3D. Geometric morphometrics based on X-ray microCT has been used previously for an exploratory analysis of the morphology of the cranium in the white and black proteus [9, 10]. However, without contrast-enhanced techniques, low-absorbing tissues such as sensory organs, lack of sufficient contrast for detailed 3D analysis. In our study, we use staining of soft tissues by phosphotungstic acid (PTA) and iodine to visualise soft tissues in the head of proteus. For the first time, we are looking to volumetric internal structures by using a non-destructive imaging technique and we show sensory organ with high spatial and contrast resolution.

This extraordinary amphibian has been an important object of study in the history of international nature research, intriguing scientists and thanks to studies in the last 300 years [3], mysteries of this cave amphibian are slowly being unravelled. However, *Proteus anguinus* still leave gaps in our knowledge about its ecology, evolution and physiology. Our high-resolution microCT scans together with 3D models of anatomical structures in the head could help to understand the nature and origin of the mechanisms behind its adaptations to the subterranean lightless environment, which led to the acquisition of troglomorphic features.

Methods

Sample preparation

The approval for the capture, handling, maintenance and breeding in captivity of the animals used in the study was granted by the Ministry of the Environment and Spatial Planning of the Republic of Slovenia, Slovenian Agency for the Environment (Permits no. 35701-36/01, 35601-95/2009-4 and 35601-132/2014-4), by the Italian Republic, Friuli Venezia Giulia Region (Permit no. 4105/6MU4/95/04/12), and by the Italian Republic, Ministry of Ecological Transition (Permits no. 3006/015590-93, 39/04). No animals were sacrificed for this study. Our experimental plan involved scanning different proteus and axolotl samples at different stages to study various parts of the skull and sensory organs.

Specimens were stored in 75% ethanol. Specimens of *Ambystoma mexicanum* were reared at the Speleovivarium Erwin Pichl (Italy) since 2004 and include 5-year-old adult female (died in 2009) with the length of 194 mm and a larva, 6 days old (died in 2012), with the length of 11 mm.

Specimens of *Proteus anguinus anguinus*: larva, 3 weeks old (died in 2007), length 23 mm, captive breeding originating from the Postojna-Planina Cave System, from the collection of the Tular Cave Laboratory (Slovenia); juvenile, 35 mm long, collected in a spring near Metković (Croatia), from the collection of the Department of Biology, Biotechnical Faculty, University of Ljubljana (Slovenia); adult, sex unknown, length 276 mm, collected in Postojna-Planina Cave System (Slovenia, 1989), died in captivity of the Speleovivarium in 1999, from the collection of the Speleovivarium Erwin Pichl (Italy).

We modified a contrasting protocol initially developed by Brian Metscher [11] that has been successfully applied on salamander tissues before [12]. Larvae samples, both proteus and axolotl, and juvenile proteus were stained with 1% phosphotungstic acid (PTA) in 90% MeOH for seven weeks. The solution was changed with the fresh one weekly. The adult proteus and axolotl specimens were stained with 2% iodine in 90% methanol for six weeks. Subsequently, the samples were rehydrated in ethanol series (90%, 80%, 70% and 50%) and fixed in polyimide tubes filled with 1% low-melting agarose gel to prevent the sample movement during the stage rotation.

Image acquisition

The samples were scanned using a laboratory X-ray microCT system GE Phoenix v|tome|x L 240 (GE Sensing & Inspection Technologies GmbH, Germany). The system was equipped with a 180 kV/15 W maximum power nanofocus X-ray tube and a high-contrast flat panel detector DXR250 with 2,048 × 2,048 pixels resolution and (200 × 200) μm^2 pixel size. 2,000 projections over a total scan angle of 360° were acquired with an exposure time of 900 ms per projection. Each projection was captured three times, and an average of the signal was used to improve the signal-to-noise ratio. The acceleration voltage of the X-ray tube was set to 60 kV and the tube current to 200 µA for larval and juvenile proteus; 80 kV and 250 µA were used, respectively, for Voltage and current for adult samples. The X-ray beams of lower energies were filtered with a 0.2 mm-thick aluminium plate for larval and with a 1 mm-thick aluminium plate for adult samples. The voxel size of the reconstructed data were the followings: 3.5 µm for juvenile proteus, 5.8 µm for larval proteus, 25 µm for adult proteus, 2.8 µm for larval axolotl and 27.5 µm for adult axolotl.

Tomographic data processing

The tomographic reconstruction was performed using GE phoenix datos|x 2.0 software (GE Sensing & Inspection Technologies GmbH, Germany). A segmentation procedure was then applied to reconstructed slices. The Avizo 7.1 (Thermo Fisher Scientific, USA) image processing software was used for semi-automatic segmentation [13-15] of structures in the head. To make the load of 3D segmentation volume smaller, every 3rd slice was manually segmented, and the rest was calculated by linear interpolation between manually segmented slices by the operator [15]. We converted the semi-manually segmented models in to polygonal meshes and imported this in VG Studio MAX 2.2 software (Volume Graphics GmbH, Germany) for 3D visualisations.

Data validation and quality control

By contrast-enhanced X-ray microCT scanning, we were able to visualise the internal structures of proteus head. Figure 1 shows the manually segmented cartilaginous chondrocranium as well as the position and the shape of the brain, the remnant eyes and the olfactory epithelium. A considerable portion of the cranial skeleton in adult proteus specimen remains cartilaginous.

A validation of the semi-automatic segmentation procedure is presented in Figure 2. The 3D models were created by an operator based on the grey-scale value contrast and the shape of the structures. The detailed procedure is described in the methods section and follows a previous study of the authors [15].

In Figure 3, we compare the microCT datasets and segmentations of the internal head structures of the larval and adult proteus to their axolotl counterparts. The head of the troglobiotic proteus is narrower and more elongated in comparison with the epigean *axolotl* which doesn't live in caves. Our 3D segmentations show remnant eyes at larval and juvenile proteus, but no remnant of eyes was noticed in the adult specimen. The progressive degeneration of the eye in the development of the proteus can often lead to the apparent disappearance of the eye in the adult animal [16]. On the other hand, 3D models of axolotl clearly show the eyes together with an optic nerve that leads to the brain in all stages. We would like to also point out on size

of the olfactory epithelium which plays an important role in foraging and communication of proteus living in complete dark.

Re-use potential

Museum-type documentation of rare and endangered species

The presented datasets are giving insight not only to life of *Proteus anguinus*. Together with 3D datasets of *Ambystoma mexicanum*, they can be important study material to guide the conservation efforts to preserve these endangered amphibians. According to the global assessment of the International Union for Conservation of Nature, 43% of amphibian species are in decline while 32% are threatened with extinction [17], proteus is currently classified as vulnerable. The X-ray microCT method enables anatomical studies without damaging the morphology of the specimens and is therefore exceptionally appropriate for studying endangered species with limited amount of available specimens. Semi-automatically segmented images and the extracted 3D models could also be taken as an input into machine learning algorithm. The field of image processing is becoming dominated by deep learning algorithms and convolutional neural networks [18]. Creating online database could be also beneficial for student studies and distant learning. Especially, last year showed the importance of easy access to online study material because of Covid-19 restrictions.

Perspectives: Synchrotron-based microCT studies

By X-ray microCT scanning with conventional source, we obtained data of excellent quality on which single cells could be identified in the cartilaginous elements (Figure 4). Despite the fact that the cells can be visually detected, their automatic segmentation and quantification is further challenging. The potential of X-ray microCT imaging with synchrotron sources for the study of 3D-cell distribution was demonstrated in our previous study on salamander limbs [12]. The high photon flux, nearly-parallel X-ray beam geometry, high spatial coherence and spatial resolution (down to 1 µm) of synchrotron microCT achieved the cellular resolution for a quantitative analysis of cell distribution. A comparison of the conventional and synchrotron X-ray microCT results is reported in Figure 4. Synchrotron data were obtained at the SYRMEP beamline of the Elettra synchrotron facility [19] by using a filtered white beam working in phase-contrast mode. Such data

were used for the study of polarization of cells in the extracellular matrix in salamander limbs [12] or can be used to mathematical modelling regarding joint shaping [20] or more general regenerative dynamics in various skeletal and non-skeletal tissues. We also show the potential of synchrotron imaging on the head structures of axolotl larvae. Also here, the improvement in contrast and spatial resolution is evident despite of some artefacts related to a slight sample movement in the case of synchrotron data (Figure 4). The processing and analysis of the synchrotron data will be the subject of a future investigation.

Research outlook

The morphology of the cranium carries important information related to mechanics involved in feeding, as well as competitive, reproductive, and anti-predatory behaviour. Even small differences in cranial skeleton may have important biomechanical and ecological implications [21]. The most detailed description of the *Proteus anguinus* skull yet are those of Dolivo-Dobrovolsky [22, 23], Ivanović with co-authors [9], Papi et al. [10] and Bizjak Mali & Sket [24]. MicroCT contrast-enhanced data include overall 3D information and further segmentation and work on the provided data can be taken as an input for various type of analyses (Figure 5). The visualisation of muscles of upper and lower jaw could be used for a bite analysis that may shed light on the predatory abilities of proteus.

In general, our datasets will help to investigate how exactly the head undergoes the evolutionary change of its shape, being an integral entity with adapted muscle-skeletal apparatus, nervous system and sensory organs. Because proteus is a cave-dwelling animal, the craniofacial design underwent a number of cave-related evolutionary adaptations, acquiring protracted and longitudinally-elongated skeletal elements, in comparison to an epigean axolotl, subjected in the comparative microCT scanning analysis and in-depth 3D analysis. Indeed, when compared to proteus, axolotl exhibits a wider skull with a massive jaw. Rather than unique property of proteus, elongated skull, body and extremities are common troglomorphism and one of the most prominent examples of converged evolution of organisms inhabiting subterranean habitats from invertebrates to vertebrates [25, 26]. In that view, results of our study can provide insight in evolutionary trends in adaptive morphological traits as a result of convergent evolution across phyla. Even without

evolutionary comparison across phyla, our results might enable further studies on evolution of the skeletal parts in the adaptive landscapes individually for closely related species.

Not only evolutionary questions can be tackled with these data. For instance, to investigate if the elongated skull shape develops evenly with the growth of the animal, we investigated the larval proteus. The larva of proteus, although being small, revealed their miniaturized protracted chondrocrania with similar spatial proportions to the adult form. Thus, the elongated body and stretched cranium may indicate possible benefits at any phase of development. The possibility of such comparative developmental and growth-related studies opens up new opportunities to look into the dynamics of development of a skeletal shape in relation to the specifics of the environment.

However, when it comes to the adaptations in sensory organs, the 3D-analysis of the head revealed major differences in visual and olfactory systems of proteus and axolotl. Firstly, proteus is blind, a typical troglomorphic trait which is explained by the troglobiotic way of living. The eye development in proteus larva begins as in other amphibians. The regression of almost normally formed eyes starts soon after hatching and gradually leads to a considerable reduction of the eyes in adult proteus [16], while surface-dwelling axolotl develops fully functional eyes. The morphological comparisons between the proteus developmental stages with their gradual reduction of eyes may improve our knowledge on the mechanisms of eye degeneration in proteus and possibly other similar blind cave-dwellers. Moreover, the eyeballs develop in a complex conjunction with muscular apparatus [27] and optical nerve. Here, in proteus, it is possible to question how the induction and degeneration of auxiliary tissues is achieved during the degradation of the pre-shaped eyeballs.

The animals with eye-regression are generally known to inhabit caves, where the vision is nearly useless. The blind Mexican cave fish (*Astyanax mexicanus*) is one of the popular model systems to study the loss of vision and developmental arrest of the visual system [28]. However, the blind fish and the other models, including proteus, still have a great potential for comparison with other eyeless vertebrates [29]. Therefore, our results

and datasets have the potential to enable new insights into the evolutionary trends in eye development and degeneration strategies in cave-dwellers across vertebrate clades.

The olfaction plays an important role in the life of the salamanders. The analysis of 3D-rendered olfactory organs in axolotl and proteus revealed striking differences between surface and cave-dwelling species. Elongated and tube-shaped olfactory cavities in proteus likely emerge as another adaptation to the cave environment, where enhanced olfaction capabilities pose an advantage in the absence of visual signals. Comparing to axolotl, elongated olfactory cavities of proteus might enable higher dynamic range of sensitivity, due to more efficient longitudinal diffusion of signals upon the entry via nostril openings. In line with this, the olfactory nerves of proteus are also considerably elongated, which is explained by longer rostral part of the skull in proteus. Therefore, the studies of evolutionary divergence of the olfactory system, its sensitivity and general design, could benefit from the deposited datasets.

Finally, salamanders represent a well-established model system for research of regeneration, and the fundamental principles of multi-tissue regeneration have been already revealed [30]. Regeneration of proteus has been described previously, yet the provided data offer an important insight into the evolutionary differences in regeneration among salamanders with fundamentally different lifestyles.

Availability of supporting data

The data sets supporting the results of this article are available in the Gigascience Database repository. We provide reconstructed slices as DICOM image stacks and segmented structures in head in STL (Standard Triangle Language) format. The folders are structured so that each folder represents one sample containing folder with DICOM stack and folder with STL files. DICOM image stack can be opened in any image viewer supporting this format, we recommend ImageJ for viewing the data [31]. To explore datasets in 3D, specialized free viewers are available - Drishti [32], DragonFly (Object Research Systems ORS Inc, Montreal, Canada) or others. For analysis and further segmentation, we recommend the use of ITK-SNAP [33] or commercial software, e.g., Avizo (Thermo Fisher Scientific, USA) or VG Studio MAX (Volume Graphics GmbH, Germany). The detail description and the manual for segmentation of biological data can be found in our previous works

[15, 34]. The STL files of the embryo heads can be also explored in 3D mesh viewers; a popular free open-source software, e.g., MeshLab [35] or Blender [36].

Figure titles and legends

Figure 1: 3D reconstructions of *Proteus anguinus anguinus* head based on X-ray microCT data (note different scaling). Larva (top), juvenile (middle) and adult (bottom) proteus. Images in the first column show dorsal 3D renderings of the head with skin. Dorsal, lateral and frontal views of the segmented internal structures are shown in second to fourth column.

Figure 2: Semi-automatic segmentation of X-ray microCT data. Raw CT slice and corresponding segmented structures in the head of a juvenile *Proteus anguinus anguinus*. The green plan on the 3D model (right image) indicates the cross-section of the presented CT slice.

Figure 3: Comparison of the larval and adult specimens of *Proteus anguinus anguinus* with *Ambystoma mexicanum*.

Figure 4: Nearly-cellular resolution of the cartilage elements in juvenile proteus obtained by a microCT with a conventional source. White spots represent cell nuclei. Top: 3D detail of cartilaginous 1st basibranchial element of the hyobranchial apparatus (yellow colour) with three orthogonal CT slices. Comparison of synchrotron imaging in comparison with conventional X-ray source; the brain of *Ambystoma mexicanum* and its eye. Some artefacts related to a slight sample movement are visible in synchrotron images.

Figure 5: Potential of contrast-enhanced X-ray microCT data. Segmentation of soft tissues in the head of the adult *Ambystoma mexicanum*. Presented 3D models based on X-ray microCT data can be employed as an input for further analyses.

Declarations

List of abbreviations

MicroCT - X-ray computed microtomography; PTA – phosphotungstic acid; STL – Standard Triangle Language, 3D – three-dimensional

Consent for publication

Not applicable.

Competing interests

The authors declare no competing interests.

Funding

We acknowledge CzechNanoLab Research Infrastructure supported by MEYS CR (LM2018110). MT acknowledges grant CEITEC VUT-J-21-7145, the Brno City Municipality as a Brno Ph.D. Talent Scholarship Holder and Martina Roeselova Memorial Fellowship. JG was funded the Fonds Wetenschappelijk Onderzoek – Vlaanderen by a postdoctoral fellowship (Grant No. 12R5118N).

Authors' contributions

M.T.: Writing – original draft, Visualization; L.M.: Conceptualization, Methodology; E.M.: Investigation, Writing – original draft; G.A.: Investigation, Writing – original draft; M.A.: Investigation, Writing – original draft; R.K.: Validation, Writing – review & editing; L.B-M.: Validation; T.Z.: Project administration; M.K.: Methodology; F.P.: Methodology; J.G.: Data curation; A.B.: Data curation; A.H.: Project administration; I.A.: Conceptualization, Project administration; J.K.: Funding acquisition , Supervision

References

1. Aljančič, G. History of research on Proteus anguinus Laurenti 1768 in Slovenia. *Folia biologica et geologica* **vol. 60**, 39-69 (2019).

2. Laurenti, J. Synopsis Reptilium. *J.T. de Trattnern, Viennae* 35-36 (1768).

3. von Schreibers, K. A. historical and anatomical description of a doubtful amphibious animal of Germany, called, by Laurenti, Proteus anguinus. *Philosophical Transactions* **vol. 91**, 241–264 (1801).

4. Charles, D. On the Origin of Species. *John Murray* (1859).

5. Proteus Genome. www.proteusgenome.com (2019).

6. Schlegel, P.A., Steinfartz, S. & Bulog, B. Non-visual sensory physiology and magnetic orientation in the Blind Cave Salamander, Proteus anguinus (and some other cave-dwelling urodele species). Review and new results on light-sensitivity and non-visual orientation in subterranean urodeles (Amphibia). *Animal Biology* **vol. 59**, 351-384 (2009).

7. Bulog, B. & Schlegel, P. Functional morphology of the inner ear and underwater audiograms of Proteus anguinus (Amphibia, Urodela). *Pflügers Archiv - European Journal of Physiology* **vol. 439**, r165-r167 (2000).

8. Hervant, F., Mathieu, J. & Durand, J. Behavioural, physiological and metabolic responses to long-term starvation and refeeding in a blind cave-dwelling (Proteus anguinus) and a surface-dwelling (Euproctus asper) salamander. *Journal of Experimental Biology* **vol. 204**, 269-281 (2001).

9. Ivanović, A., Aljančič, G., Arntzen, J.W. & van Rooijen, J. Skull shape differentiation of black and white olms (Proteus anguinus anguinus and Proteus a. parkelj): an exploratory analysis with micro-CT scanning. *Contributions to Zoology* **vol. 82**, 107-114 (2013).

10. Papi, F., Mauri, E., Pesaro, S., Tromba, G. & Mancini, L. Analysis of the skull of Proteus anguinus anguinus by high-resolution X-ray computed microtomography. *Natura Sloveniae* **2**, 43-45 (2018).

11. Metscher, B. MicroCT for comparative morphology: simple staining methods allow high-contrast 3D imaging of diverse non-mineralized animal tissues. *BMC Physiology* **vol. 9**, (2009).

12. Tesařová, M., Mancini, L., Simon, A., Adameyko, I., Kaucká, M., Elewa, A., Lanzafame, G., Zhang, Y., Kalasová, D., Szarowská, B., Zikmund, T., Novotná, M. & Kaiser, J. A quantitative analysis of 3D-cell distribution in regenerating muscle-skeletal system with synchrotron X-ray computed microtomography. *Scientific Reports* **vol. 8**, (2018).

13. Bayle, P., Braga, J., Mazurier, A. & Macchiarelli, R. Dental developmental pattern of the Neanderthal child from Roc de Marsal: a high-resolution 3D analysis. *Journal of Human Evolution* **vol. 56**, 66-75 (2009).

14. Zanolli, C., Bondioli, L., Mancini, L., Mazurier, A., Widianto, H. & Macchiarelli, R. Brief communication: Two human fossil deciduous molars from the sangiran dome (Java, Indonesia). *American Journal of Physical Anthropology* **vol. 147**, 472-481 (2012).

15. Tesařová, M., Zikmund, T., Kaucká, M., Adameyko, I., Jaroš, J., Paloušek, D., Škaroupka, D. & Kaiser, J. Use of micro computed-tomography and 3D printing for reverse engineering of mouse embryo nasal capsule. *Journal of Instrumentation* **vol. 11**, C03006-C03006 (2016).

16. Durand, J. Ocular development and involution in the european cave salamander, Proteus anguinus laurenti. *The Biological Bulletin* **vol. 151**, 450-466 (1976).

17. Stuart, S.N., Chanson, J.S., Cox, N.A., Young, B.E., Rodrigues, A.S.L., Fischman, D.L. & Waller, R.W. Status and trends of amphibian declines and extinctions worldwide. *Science* **vol. 306**, 1783–1786 (2004). doi: 10.1126/science.1103538.

18. Ronneberger, O., Fischer, P. & Brox, T. U-Net: Convolutional Networks for Biomedical Image Segmentation. in *Medical Image Computing and Computer-Assisted Intervention – MICCAI 2015* Navab, N., Hornegger, J., Wells, W. & Frangi, A. 234-241 (Springer International Publishing, 2015). doi:10.1007/978-3-319-24574-4_28.

19. Tromba, G., Longo, R., Abrami, A., Arfelli, F., Astolfo, A., Bregant, B., Brun, F., Casarin, K., Chenda, V., Dreossi, D., Hola, M., Kaiser, J., Mancini, L., Menk, R.H., Quai, E., Quaia, E., Rigon, L., Rokvic, T., Sodini, N., Sanabor, D., Schultke, E., Tonutti, M., Vascotto, A., Zanconati, F., Cova M., & Castelli, E. The SYRMEP Beamline of Elettra: Clinical Mammography and Bio-medical Applications. *AIP Conf. Proc.*, **vol. 1266**, 18-23, (2010).

20. Comellas, E., Duerr, T., Farkas, J., Monaghan, J. & Shefelbine, S. Integrating macromolecular data in a tissue-level computational model of vertebrate joint morphogenesis. in *Summer Biomechanics, Bioengineering and Biotransport Conference* (2020). doi:10.13140/RG.2.2.30787.22566.

21. van der Meij, M. & Bout, R. The relationship between shape of the skull and bite force in finches. *Journal of Experimental Biology* **vol. 211**, 1668-1680 (2008).

22. Dolivo-Dobrovolsky, V. Das Kopfskelett des Grottenolmes (Proteus anguinus Laur.). *Zoologischer Anzeiger* **57**, 281-284 (1923).

23. Dolivo-Dobrovolsky, V. Lobanja čovješke ribice (Proteus anguinus Laurenti). *Rad Jugoslavenske akademije zna-nosti i umjetnosti* **232**, 190 - 209 (1926).

24. Bizjak Mali, L. & Sket, B. History and biology of the «black proteus« (Proteus anguinus parkelj Sket & Arntzen 1994; Amphibia: Proteidae). *Folia biologica et geologica* **vol. 60**, 5-37 (2019).

25. Culver, D. & Pipan, T. *The Biology of Caves and Other Subterranean Habitats*. (Oxford University Press, 2019). doi:10.1093/oso/9780198820765.001.0001.

26. Ribera, I., Cieslak, A., Faille, A. & Fresneda, J. Historical and Ecological Factors Determining Cave Diversity. in *Cave Ecology* Moldovan, O., Kováč, Ľ. & Halse, S. 229-252 (Springer International Publishing, 2018). doi:10.1007/978-3-319-98852-8_10.

27. Comai, G., Tesařová, M., Dupé, V., Rhinn, M., Vallecillo-García, P., da Silva, F., Feret, B., Exelby, K., Dollé, P., Carlsson, L., Pryce, B., Spitz, F., Stricker, S., Zikmund, T., Kaiser, J., Briscoe, J., Schedl, A., Ghyselinck, N., Schweitzer, R., Tajbakhsh, S. & Hughes, S. Local retinoic acid signaling directs emergence of the extraocular muscle functional unit. *PLOS Biology* **vol. 18**, (2020).

28. Jeffery, W.R. Emerging model systems in evo-devo: cavefish and microevolution of development. Evolution & development **vol. 10**, 265-272 (2008).

29. Tovar, R., Cantu, V., Fremaux, B., Gonzalez Jr, P., Spikes, A. & García, D. Comparative development and ocular histology between epigean and subterranean salamanders (Eurycea) from central Texas. *PeerJ* **vol. 9**, (2021).

30. Kumar, A., Godwin, J., Gates, P., Garza-Garcia, A. & Brockes, J. Molecular Basis for the Nerve Dependence of Limb Regeneration in an Adult Vertebrate. *Science* **vol. 318**, 772-777 (2007).

31. Schneider, C., Rasband, W. & Eliceiri, K. NIH Image to ImageJ: 25 years of image analysis. *Nature Methods* **vol. 9**, 671-675 (2012).

32. Limaye, A. & Stock, S. Drishti: a volume exploration and presentation tool. in 85060X-doi:10.1117/12.935640.

33. Yushkevich, P.A., Gao, Y. & Gerig, G. ITK-SNAP: An interactive tool for semi-automatic segmentation of multi-modality biomedical images. in *2016 38th Annual International Conference of the IEEE Engineering in Medicine and Biology Society (EMBC)* 3342-3345 (IEEE, 2016). doi:10.1109/EMBC.2016.7591443.

34. Tesařová, M., Heude, E., Comai, G., Zikmund, T., Kaucká, M., Adameyko, I., Tajbakhsh, S. & Kaiser, J. An interactive and intuitive visualisation method for X-ray computed tomography data of biological samples in 3D Portable Document Format. *Scientific Reports* **vol. 9**, 1-8 (2019).

35. Cignoni, P., Callieri, M., Corsini, M., Dellepiane, M., Ganovelli, F. & Ranzuglia, G. MeshLab: an Open-Source Mesh Processing Tool. (2008).

36. Hess, R. *The essential Blender: guide to 3D creation with the open source suite Blender*. (No Starch Press, 2007).

Figure 1

Click here to access/download;Figure;Tesarova_Proteus_Figure1.png

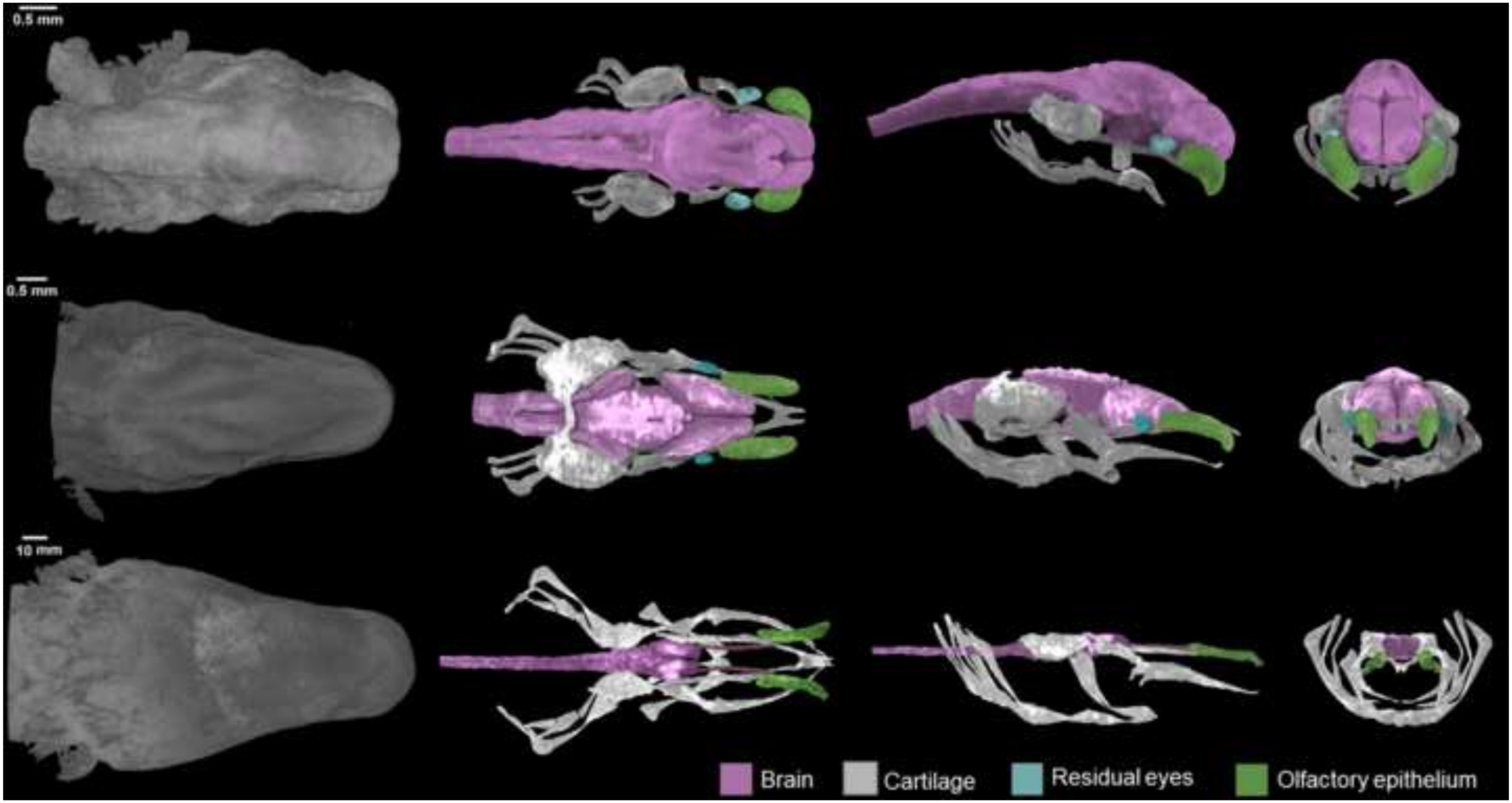

Figure 2

Click here to access/download;Figure;Tesarova_Proteus_Figure2.png

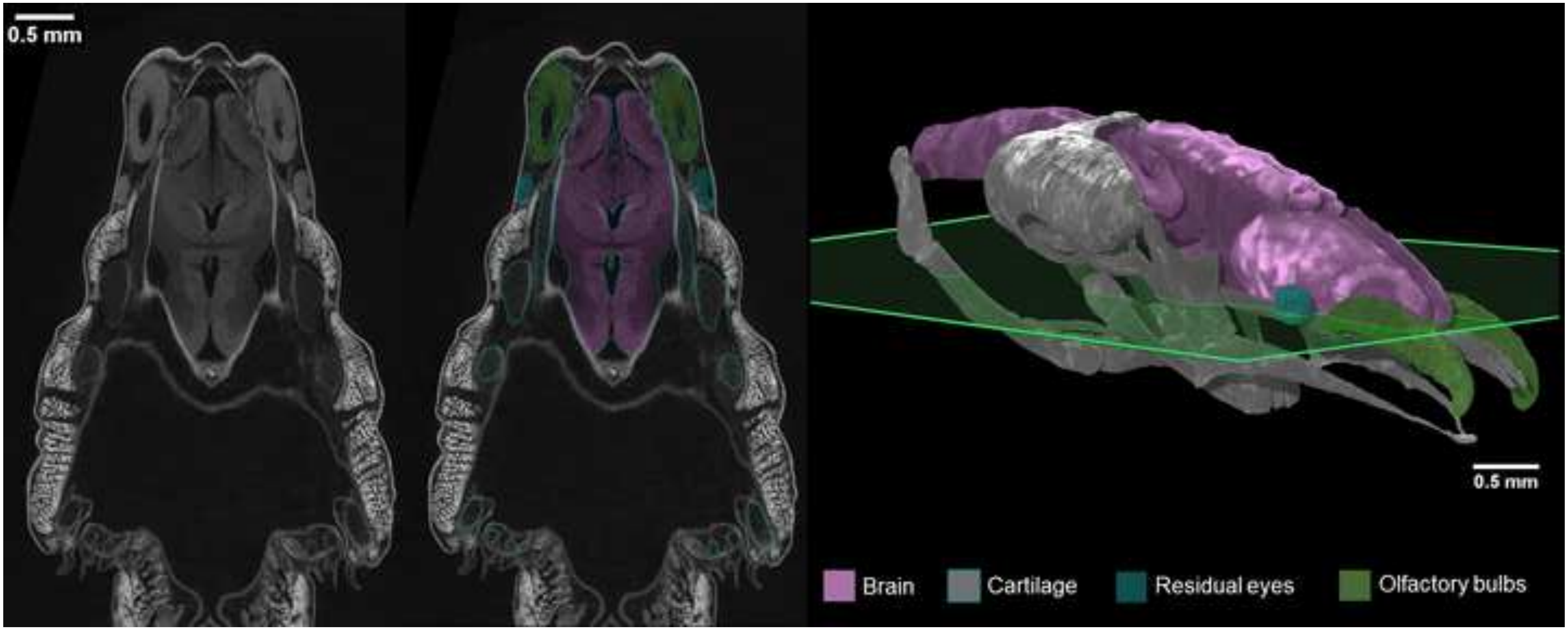

Figure 3
Click here to access/download;Figure;Tesarova_Proteus_Figure3.png

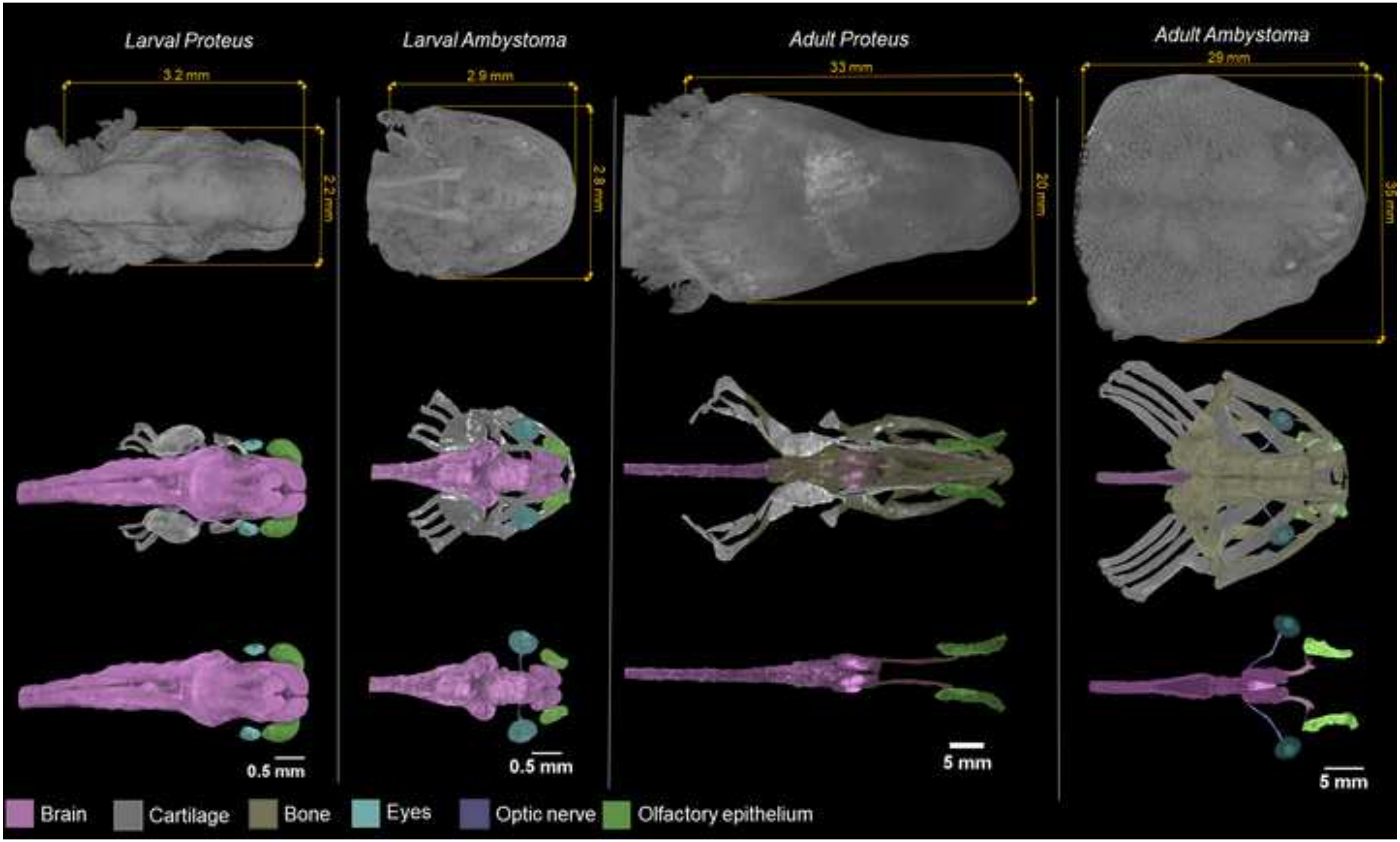

Figure 4

Click here to access/download;Figure;Tesarova_Proteus_Figure4.png

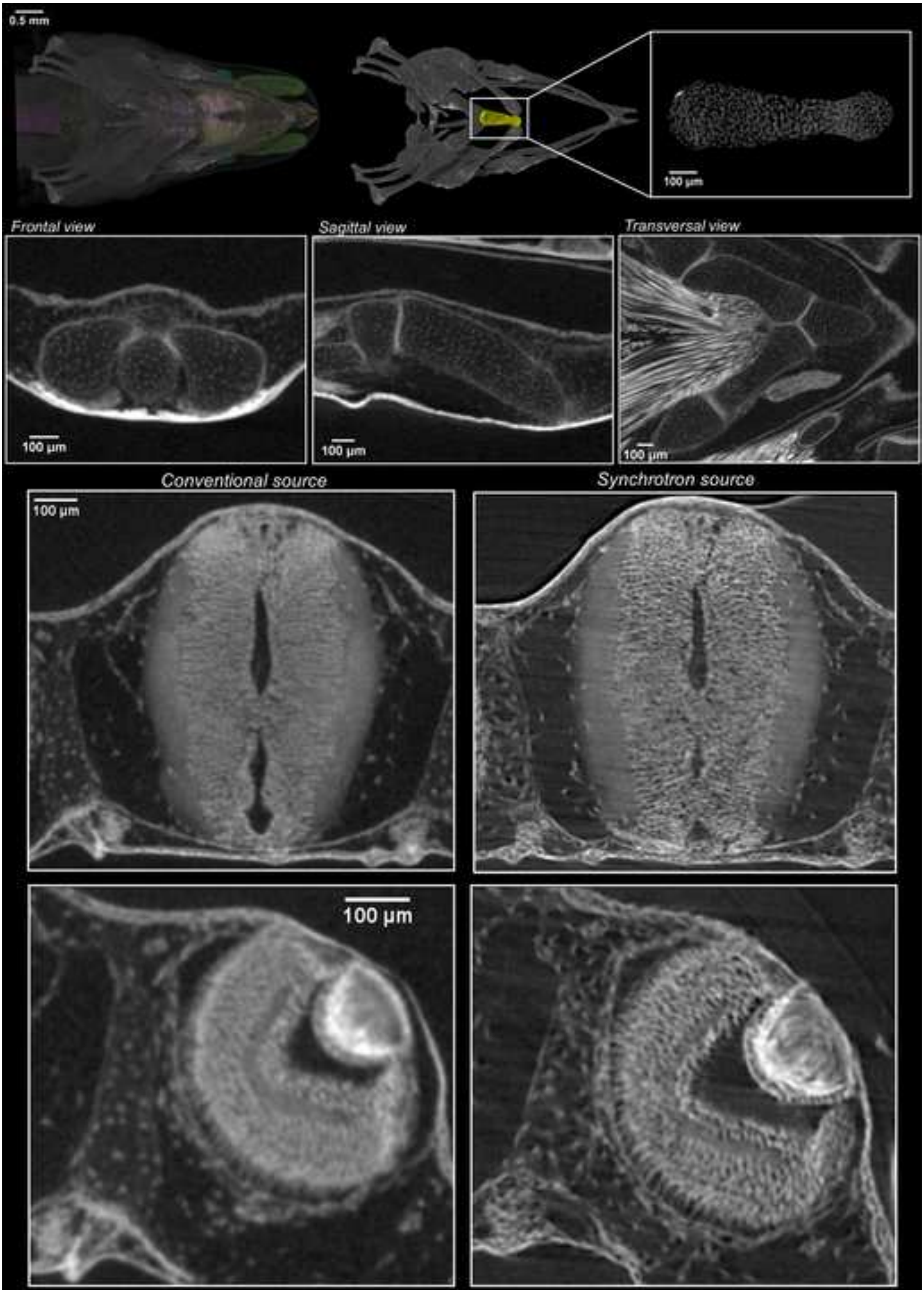

Figure 5

Click here to access/download;Figure;Tesarova_Proteus_Figure5.png

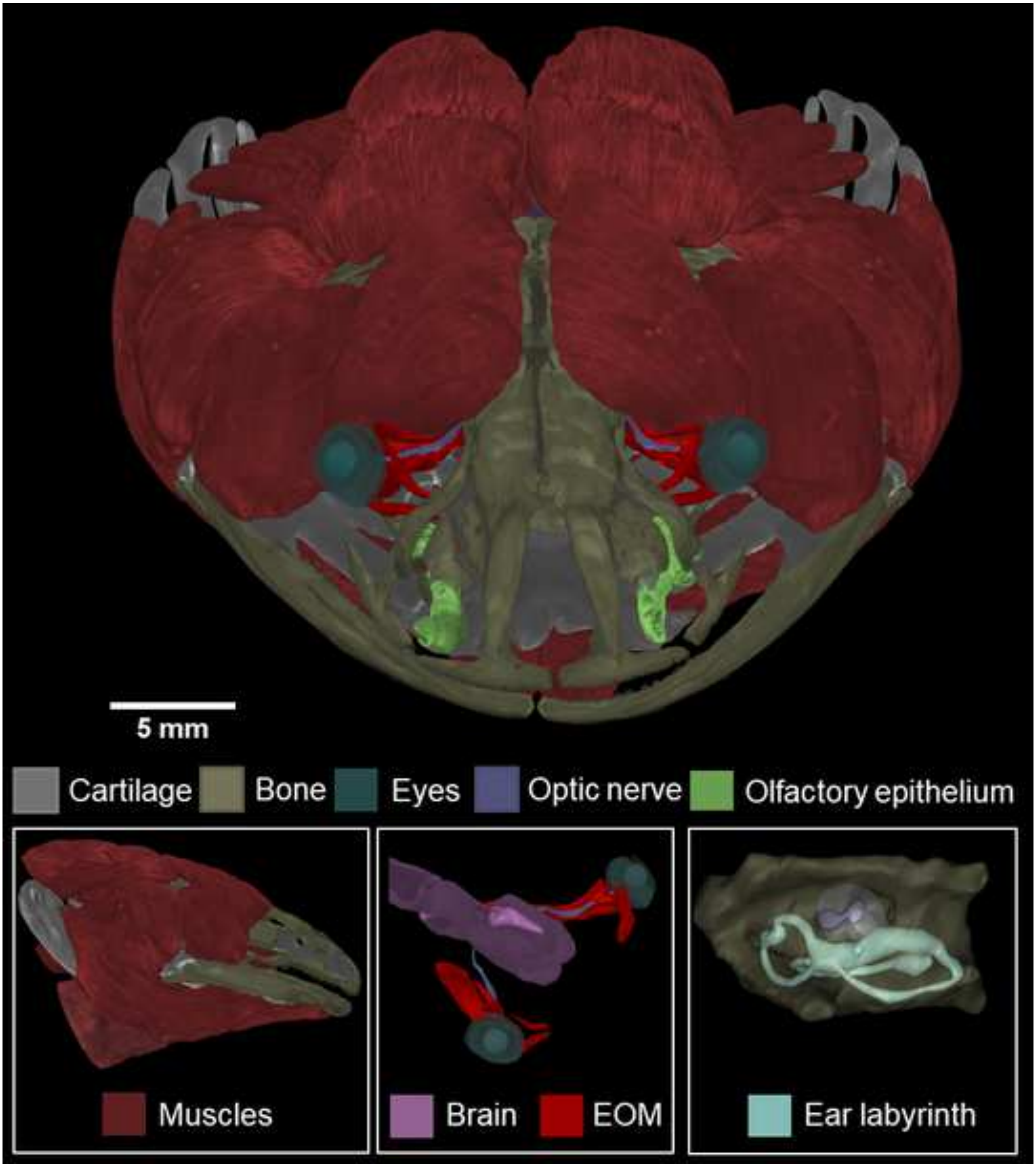

Supplement: giac030_GIGA-D-21-00259_Original_Submission [file giac030_giga-d-21-00259_original_submission.pdf]
