# Supplementary material for: Living in darkness: Exploring adaptation of Proteus anguinus in 3 dimensions by X-ray imaging
Source: Gigascience. 2022 Apr 5;11:giac030. doi: 10.1093/gigascience/giac030 (PMC8982192; doi:10.1093/gigascience/giac030)

GigaScience

Living in darkness: Exploring adaptation of Proteus anguinus in 3D by X-ray imaging

--Manuscript Draft--

**Manuscript Number:**	GIGA-D-21-00259R1
**Full Title:**	Living in darkness: Exploring adaptation of Proteus anguinus in 3D by X-ray imaging
**Article Type:**	Data Note
**Funding Information:**	Ministerstvo Školství, Mládeže a Tělovýchovy (LM2018110) — Prof. Jozef Kaiser<br>Vysoké Učení Technické v Brně (CEITEC VUT-J-21-7145) — Mrs. Markéta Tesařová<br>Fonds Wetenschappelijk Onderzoek (12R5118N) — Dr. Jana Goyens
**Abstract:**	Background: The lightless caves can harbour a wide range of living organisms. Cave animals have evolved a set of morphological, physiological and behavioural adaptations known as troglomorphisms, enabling their survival in perpetual darkness, narrow temperature and humidity ranges, and nutrient scarcity of subterranean environment. In this study, we focused on adaptations of skull shape and sensory systems in blind cave salamander, Proteus anguinus, also known as olm or simply proteus - the largest cave tetrapod and the only European amphibian living exclusively in subterranean environments. This extraordinary amphibian compensates the loss of sight by enhanced non-visual sensory systems including mechanoreceptors, electroreceptors and chemoreceptors. We compared developmental stages of Proteus anguinus with Ambystoma mexicanum, also known as axolotl, to make an exemplary comparison between cave- and surface-dwelling paedomorphic salamanders.<br>Findings: We used contrast-enhanced X-ray computed microtomography for the 3D segmentation of the soft tissues in the head of Proteus anguinus and axolotl. Sensory organs were visualised to understand how the animal is adapted to living in complete darkness. X-ray microCT datasets were provided along with 3D models for larval, juvenile and adult specimens, showing the cartilage of the chondrocranium, the position, shape and size of the brain, eyes and olfactory epithelium.<br>Conclusions: Proteus anguinus still keeps some of its secrets. Our high-resolution X-ray microCT scans together with 3D models of the anatomical structures in the head may help to understand the nature and origin of the mechanisms behind its adaptations to the subterranean environment, which led to series of troglomorphies.
**Corresponding Author:**	Jozef Kaiser<br><br>CZECH REPUBLIC
**Corresponding Author Secondary Information:**	
**Corresponding Author's Institution:**	
**Corresponding Author's Secondary Institution:**	
**First Author:**	Markéta Tesařová
**First Author Secondary Information:**	
**Order of Authors:**	Markéta Tesařová<br>Lucia Mancini<br>Edgardo Mauri<br>Gregor Aljančič<br>Magdalena Năpăruş-Aljančič<br>Rok Kostanjšek

Powered by Editorial Manager® and ProduXion Manager® from Aries Systems Corporation

	Lilijana Bizjak Mali
	Tomáš Zikmund
	Markéta Kaucká
	Federica Papi
	Jana Goyens
	Anass Bouchnita
	Andreas Hellander
	Igor Adameyko
	Jozef Kaiser
**Order of Authors Secondary Information:**	

Response to Reviewers:

Dear Editor,
Thank you for the possibility to revise our manuscript "Living in darkness: Exploring adaptation of Proteus anguinus in 3D by X-ray imaging". We went through the reviewers' comments and tried to do our best in order to improve the manuscript for the community of biologists as well as for imaging experts. We did new segmentation of craniofacial muscles and ear labyrinths which can be found in new Figure 5. We also took in account comments regarding more detailed datasets in the repository and we created STL files of the surface for the all 5 samples and we also added binary masks for each segmented structure. We are also thankful reviewers for their comments regarding the writing style and the manuscript was sent to the language check. Below, you can find our point-by-point response to reviewers' comments.
We hope this revision is suitable for publication GigaScience as a Data Note.

Yours Sincerely,
Prof. Jozef Kaiser
Research Group Leader
Advanced Instrumentation and Methods for Materials Characterization

tel: +420 541149700, +420 731141281
e-mail: jozef.kaiser@ceitec.vutbr.cz
web: www.ceitec.eu

Reviewer reports:

Reviewer #1: The blind white olm (Proteus anguinus anguinus) are unique in many aspects of its biology, including geographic distribution, development and particularly its salient troglomorphism, which resulted from its morphological adaptation to underwater cave-dwelling lifestyle. As mentioned in this technical study, our knowledge on this particular salamander species was accumulated bit by bit over the past few hundred years. Now we are standing at a point where details of morphological and developmental changes of this taxon can be better revealed by non-destructive methods based on more readily available specimens raised in labs due to the advancement of high-resolution microCT scan and contrast enhancing techniques. Sharing of high-resolution CT images of endangered species that are significant to advance our understandings of certain scientific questions should always be encouraged.

This manuscript authored by Tesařová et al. provided a dataset of contrast-enhanced microCT images for the head region of three specimens (a larva, a juvenile and an adult) of Proteus anguinus anguinus and also images for two specimens (a larva and an adult) of Ambystoma mexicanum for the purpose of comparative studies. Besides many annotations I made in the word file as attached, here are the rest of my other concerns:

Response:
Dear Reviewer,
Thank you for your response and all the comments regarding our manuscript. We considered annotations in the word document and we think it improved a manuscript,

Powered by Editorial Manager® and ProduXion Manager® from Aries Systems Corporation

so we are really grateful for that! Below, you can find response for the rest of your concerns below:

1) The comparison between the olm and the axolotl is reasonable considering both species are neotenic and one is cave dwelling and the other is surface dwelling, and therefore avoid introducing noises from changes created by metamorphosis; however, why not choose any neotenic species in the sister group genus of Proteus, Necturus, for comparison, considering their phylogenetic closeness and disparate lifestyles (Necturus is surface dwelling in slow-moving streams)?

Response: This is a very interesting comment and we thank you for this perspective. It is true that comparison with neotenic species in the sister group genus of Proteus could give interesting data. We decided for analyses of Ambystoma mexicanum for its wide use in developmental and regenerative studies. Also, Proteus anguinus and Ambystoma mexicanum share neoteny as a prominent attribute and have similar characteristics in regard to predation, aquatic life and reproduction. Proteus anguinus is currently the only neotenic genus living on the European continent. It is represented by two subspecies: Proteus anguinus anguinus (Laurenti 1768), strictly troglomorphic, and pigmeted subspecies Proteus anguinus parkelj (Skeet & Arntzen, 1994) of less-troglomorphic morphology. In the past, this was very different. The fossil record between the Late Cretaceous and the thermal maximum of Paleocene-Eocene revealed the presence of a large number of caudates in Europe including Cryptobranchidids, Batrachosauroidids and Ambystomidids (Skutschas & Gubin 2012). Many specimens with external gills and neotenic features were found. Therefore, the ancestors of Ambistomidae and Proteidae lived on the European continent about 50 Ma years ago sharing environments favorable to the amphibians spread (Vasilyan & Yanenko 2020). We were then motivated to compare specimens of Proteus and Ambystoma belonging to two families that in ancestral times shared marshy environments on the European continent. We have reason to believe that such a comparison may reveal similarities and / or differences on the morphologies examined which would otherwise be difficult to detect. However, consider other species in our future studies.

2) After checking the dataset by loading dicom files into software VG Studio, it seems that the larval specimen of the olm has some shrink in the head region, which may be unavoidable, but I would appreciate if you can enrich the descriptions for the contrast-enhancing experimental procedures by providing the length of the rehydration process for each specimen.

Response: Thank you for this remark. We added the following information to the method section: Subsequently, the samples were gradually rehydrated in ethanol series (90%, 80%, 70% and 50%), one day for each concentration (i.e. 4 days of rehydration).

3) Most of the datasets contain a complete head region except the one for the adult specimen of the axolotl, which has the posterior part of the hyobranchial apparatus missing. It is highly recommended to provide a more complete dataset for the axolotl adult specimen.

Response: We substituted dataset with more complete dataset for adult axolotl. As we focused on the segmentation of structures in the head, we chose only head area and did not realize that hyobranchial apparatus is missing. Now, there is a new DICOM series for adult axolotl containing also neck part.

4) The dataset for the adult specimen of the olm can not be properly loaded in visualization softwares like VG Studio and Photoshop, because it has three damaged dicom files, i.e., "Proteus_anguinus_adult_0661.dcm", "Proteus_anguinus_adult_2023.dcm" and "Proteus_anguinus_adult_2349". The first image is 590 kb, and the latter two are 0 kb in size, in contrast to most other images in the same dataset which are 637 kb. It's also noticeable that 233 images ranging from "Proteus_anguinus_adult_2116.dcm" to "Proteus_anguinus_adult_2348.dcm" are 2317 kb, and can not be properly loaded into software with files of 637 kb.

Response: Thank you for drawing our attention to this technical error that must have

	happened during the preparation of the data. We substitute the DICOM folder with corrected files and now the dataset is complete.<br><br>Generally speaking, the manuscript is clearly written, and the dataset is easily accessible and well controlled. I look forward to its formal publication. Feel free to contact me (jia.jia@ucalgary.ca) directly if any of my comments are unclear.<br><br>Best wishes,<br>Jia<br>October 8, 2021<br><br>Response: Dear Jia, thank you again for your time spent on our manuscript and for the all helpful comments and notices. If you feel there is still space for improvement, we still be happy to hear your comments.<br>With best wishes<br>Jozef<br><br>Reviewer #2: The authors have studied the cranial anatomy of the Proteus anguinus by x-ray tomography. In particular, they have imaged and segmented the sensory system of these animals across three different stages of development: larval, juvenile and adult. They find that although eye development starts in the larval stage, it gradually reduces its size to the point of blindness, probably due to adaptation to a cave environment. In contrast, axolotls fully develop their eyes.<br><br>Overall the scientific data provided by the authors will certainly lead to further studies on the evolution of the salamander brain in the contest of cave adaptation. However, I do not recommend the publication of this manuscript without a major revision.<br><br>The authors should segment not only the brain, olfactory epithelium, residual eye and skull but also ear labyrinth and muscles across the three different stages of development. They should provide a side-by-side comparison in Figure 5 with the axolotl giving inputs on the different predatory habits by comparing the muscles and the jaws of the two species.<br><br>Response: Dear Reviewer,<br>Thank you for your comments and time spent on reviewing our manuscript. Following your suggestion, we added segmentations and 3D visualizations of the ear labyrinth for the all 5 samples in Figure 5. We also tried to segment the facial muscles. However, segmenting the facial muscles and making the binary mask for the larvae species could be misleading as the muscles only start to form at this stage of development, and as a result there is no clear border of the muscles. Thus, we didn't add the segmentations for larvae, however, we followed your advice for the adult specimens and added the data to Figure 5. We still believe that area of craniofacial muscles for larvae could be further examined by developmental biologists as we provide complete tomographic datasets.<br><br>The authors should also double check the scale bars of the top and middle image in Figure 1 as the dimensions of the animals are significantly different between the two stages of development.<br><br>Response: Thank you for this notice, we double checked the scalebars as we also slightly changed Figure 1 with semi-transparent heads in the first column.<br><br>It is not clear Figure 2 relates to the juvenile specimens from the text and this should be improved.<br><br>Response: Thank you for this note, we made it clearer by adding a sign "Juvenile Proteus anguinus" directly in the image. Also, we added this information to the Figure legend: "Accuracy validation of semi-automatic segmentations of X-ray microCT data in juvenile Proteus anguinus anguinus".<br><br>Furthermore the presentation of the synchrotron x-ray tomography of the axolotl brain in Figure 4 is redundant given the scope of the manuscript which is the study of Proteus anguinus sensory system. Either the authors present synchrotron images of

Powered by Editorial Manager® and ProduXion Manager® from Aries Systems Corporation

the Proteus anguinus or they should omit the figure entirely as this is confusing to the readers.

Response: We thank reviewer also for this notice and we agree that it could be confusing for the readers, so we decided to change Figure 4 as you suggested and removed the synchrotron data. We also modified section “Perspective” in the text:
Perspectives: Cellular resolution
Using microCT scan with a conventional X-ray source, we obtained data of excellent quality which depict single cells in the cartilaginous elements (Figure 4). Despite the fact that the cells can be visually detected, their automatic segmentation and quantification is further challenging. The potential of X-ray microCT imaging with synchrotron sources for the study of 3D-cell distribution was demonstrated in our previous study on salamander limbs [12] and the potential for biomedical applications was shown before [19, 20]. The data with cellular resolution can be used as the input for the study of polarization of cells in the extracellular matrix in salamander limbs or for mathematical modelling of joint formation [12].

Finally, it is not clear the reason why the authors have stained the specimens with PTA and Iodine for several weeks as the contrasting protocol initially developed by Brian Metscher involves only overnight or few days of staining. Perhaps the authors could share the reason for this very long method.

Response: We thank the reviewer also for this point. Brian Metsher’s papers usually refers to small-size animals. However, the adult Proteus and adult Ambystoma are much larger in comparison with specimens as mouse embryos on which Brian Metscher demonstrates some of his experiments. This is the reason why staining took significantly longer time. We add following clarification to the text to make it clearer also for the readers: “The adult Proteus anguinus and axolotl specimens were stained with 2% iodine (instead of PTA) in 90% methanol for six weeks to ensure that the contrasting agent would penetrated to the entire sample, because iodine penetrates better than PTA.”

Reviewer #3: This Data Note showcases microCT datasets of the blind cave salamander Proteus anguinus, which is one of nature's curiosities, and also the Mexican axolotl Ambystoma mexicanum. Like other amphibians, Proteus anguinus begins eye development with the optic vesicles outgrowing from the diencephalon region of the developing brain and making contact with the surface ectoderm to initiate what is known as 'lens induction'. However, in Proteus the eyes soon regress after hatching. This adaptation to living in the dark is seen in other species, such as blind cave fish, and it is thought to be linked to the expression of key regulatory genes such as Pax6 and Shh (see Tian NM, Price DJ. Why cavefish are blind. Bioessays. 2005 Mar;27(3):235-8. doi: 10.1002/bies.20202). In addition, Proteus anguinus and the Mexican axolotl (Ambystoma mexicanum) have remarkable regenerative capabilities, and the axolotl is increasingly seen as a Model Organism for the study of regeneration (for example see Sanor LD, Flowers GP, Crews CM. Multiplex CRISPR/Cas screen in regenerating haploid limbs of chimeric Axolotls. Elife. 2020 Jan 28;9:e48511. doi: 10.7554/eLife.48511).

The larval and juvenile specimens are high quality cellular-resolution 3D models, and as with the previous GigaScience Data Note that was published by the authors (https://doi.org/10.1093/gigascience/giab012), the 3D models are high contrast by virtue of the specimens being stained with phosphotungstic acid prior to scanning. The adult specimens are of tissue-level resolution rather than cellular-level resolution, the difference in voxel size being directly linked to the physical dimensions of the sample. They are nevertheless of very high quality, and the authors further highlight that synchrotron X-ray microCT can produce superior quality images of juvenile and adult specimens (see Figure 4 in the manuscript).

A major strength of this newly submitted GigaScience Data Note is the careful delineation of key anatomical components in the scanned specimens. This involves considerable effort with every 3rd microCT slice being manually segmented and linear interpolation used to fill in the gaps. The 3D segmentations offer immense reuse potential, and enable researchers to further analyse key anatomical components -

including brain, cartilage, bone, residual eyes, optic nerve, olfactory epithelium, ear labyrinth, and extraocular muscles - by morphometry and volumetric analysis.

Eye regression in Proteus is clearly of interest from an evolutionary and developmental biology (evo-devo) perspective. In addition, the anatomical detail provided in this study allows the authors to state that, "elongated and tube-shaped olfactory cavities in proteus likely emerge as another adaptation to the cave environment, where enhanced olfaction capabilities pose an advantage in the absence of visual signals". This novel and potentially fascinating adaptation may highlight a 'trade-off' between olfaction and vision during Proteus development. The authors allude to this in the manuscript, where they state, "when it comes to the adaptations in sensory organs, the 3D-analysis of the head revealed major differences in visual and olfactory systems of proteus and axolotl".

The authors additionally highlight the iconic status of Proteus, referred to in 'On the Origin of Species', where as the authors point out "Charles Darwin attributed the reduction of eyes wholly to their disuse in darkness." In addition, the authors' highlight that Proteus is classified as vulnerable, which means a species considered to be facing a high risk of extinction in the wild. The vulnerable status of this iconic species further increases the impact of this study.

In summary, the three stages of Proteus development and two stages of Ambystoma development are of interest from an evolutionary and developmental biology (evo-devo) perspective. Ambystoma is additionally of interest as a Model Organism for the study of regeneration.

I recommend this Data Note for publication in GigaScience.

Minor comment 1

In Figure 3 (larval and adult specimens of Proteus anguinus anguinus and Ambystoma mexicanum) and Figure 5 (adult Ambystoma mexicanum), the authors refer to the following segmentations:
-Brain
-Cartilage
-Bone
-Eyes / Residual Eyes
-Optic nerve
-Olfactory epithelium / bulbs
-Ear labyrinth
-Extraocular muscles (EOM)
-Craniofacial muscle

However, the STL files submitted to GigaDB only include the following:
-Brain
-Cartilage
-Bone
-Eyes / Residual eyes
-Olfactory epithelium
-Optic nerve (larval Ambystoma mexicanum)

Can the authors please submit the following 3D segmentation files to GigaDB?
-Optic nerve
-Ear labyrinth
-Extraocular muscles (EOM)
-Craniofacial muscle

Response: Dear Reviewer,
Thank you for your comments and time spent on reviewing our manuscript. We uploaded new STL files to GigaDB for all samples where the structure appears.

Minor comment 2

Can the authors provide the masks (binary image files) that were used to create the 3D

surface reconstructions (STL format) from the volumetric DICOM image stacks? This is important for reproducibility, and for every segmentation this should include: 1) manually delineated image masks where every 3rd section was used according to the manuscript; 2) processed image masks where linear interpolation was used to fill in the gaps between manually delineated sections.

Response: We are thankful for this comment and we realize that for some purposes, masks can have different usage than STL files. Thus, we uploaded segmented masks to GigaDB for each segmented structure and add this information to “Availability of supporting data” part: For segmented structures, we also provide segmented masks as DICOM image stacks – one stack for each structure.
However, we did not upload the masks before interpolation as these data are only the preliminary step and contains artifacts caused by manual segmentation of the operator. This non-complete data are 34 GB, so we don’t see added value by adding them on the server and then someone can accidentally download these non-complete data and be confused. However, we are open to discussion regarding this topic.

Minor comment 3

Can the authors provide 3D surface reconstructions (STL format) of the whole specimens? This will provide the necessary context for enabling researchers to explore the relationship between surface anatomy and internal anatomy.

Response: Thank you for this suggestion. We uploaded STL format of the 3D surface for the all specimens to GigaDB.

Reviewer #4: Dear authors,
This is the first time I reviewed a manuscript for GigaScience. From my understanding the main aim of your study was to share a detailed 3D morphological dataset (including soft tissue) of the head of two salamander species. I guess that agrees well with the scope of GigaScience.
However I had some problems with accessing the science behind the presentation of the comparative morphology of the optic system of the cave dwelling paedomorphic olm Proteus anguinus and the well known lentic paedomorphic Axolotl Ambystoma mexicanum.

Response: Dear Reviewer,
Thank you for your comments and time spent on our manuscript, below we provide the answers for the points you raised:

(1) From my point of you should definitely present a much more detailed comparative analysis of your data, maybe in the results section, just expand it.

Response: We are thankful for this comment and we are aware that more analysis could bring greater insight to the topic. From this reason, we submitted the manuscript as the Data Note and not as the Research article, so our data and segmentations could be available for any researcher for further investigation.

(2) Then I would strongly suggest using the term "olm" instead of "proteus" (if you by all means want to apply a common name). Otherwise better apply the scientific name "Proteus anguinus".

Response: Thank you for this comment and we were discussing this matter with other co-authors whether to use “olm” or “proteus” or “Proteus anguinus” as the term differs among the literature. Based on your advice and on the text by Trontelj et al. (Recommendations for a consistent use of vernacular names for Proteus anguinus in English and Slovenian scientific texts, 2017), we decided to apply scientific name “Proteus anguinus” throughout the entire manuscript.

(3) You should be aware of that n = 2 Ambystoma mexicanum and n = 3 Proteus anguinus is not an exhaustive sample size at all.

Response: Thank you for this notice, we are aware that five samples are not an exhaustive sample size. Opposite to axolotl, proteus samples are often of an

	inadequate numerus due to the extreme rarity (e.g., only a few larvae were ever found in nature, difficult captive breeding), as well as because proteus is a protected species. In such circumstances, we believe that covering larval, juvenile and adult stages could bring interesting information. Towards with detailed contrasting, measuring and segmentation protocols, we believe that anyone could apply this procedure to specimens in their collection and compare with data provided by us.<br><br>(4) The use of English is OK in most parts of the manuscript but certainly not good enough to get published. I suggest you present the manuscript to a native speaker for a closer language check.<br><br>Response: We are thankful for this comment and for all annotations in the pdf version. We went carefully through the text and your comments. We also passed the manuscript to the native speaker for language check.<br><br>Minor comments I have made directly on the pdf version of the manuscript<br><br>But I hope my comments will aid in the revision<br><br>Bests<br><br>Alex Kupfer
**Additional Information:**	
**Question**	**Response**
Are you submitting this manuscript to a special series or article collection?	No
**Experimental design and statistics**<br><br>Full details of the experimental design and statistical methods used should be given in the Methods section, as detailed in our Minimum Standards Reporting Checklist. Information essential to interpreting the data presented should be made available in the figure legends.<br><br>Have you included all the information requested in your manuscript?	Yes
**Resources**<br><br>A description of all resources used, including antibodies, cell lines, animals and software tools, with enough information to allow them to be uniquely identified, should be included in the Methods section. Authors are strongly encouraged to cite Research Resource Identifiers (RRIDs) for antibodies, model organisms and tools, where possible.	Yes

Have you included the information requested as detailed in our Minimum Standards Reporting Checklist?	
**Availability of data and materials**<br><br>All datasets and code on which the conclusions of the paper rely must be either included in your submission or deposited in publicly available repositories (where available and ethically appropriate), referencing such data using a unique identifier in the references and in the "Availability of Data and Materials" section of your manuscript.<br><br>Have you have met the above requirement as detailed in our Minimum Standards Reporting Checklist?	Yes

Manuscript

Click here to access/download;Manuscript;Proteus_Tesarova_gigascience_r

Living in darkness: Exploring adaptation of *Proteus anguinus* in 3D by X-ray imaging

Authors:

Markéta Tesařová[1], Lucia Mancini[2], Edgardo Mauri[3], Gregor Aljančič[4], Magdalena Năpăruş-Aljančič[4,5], Rok Kostanjšek[6], Lilijana Bizjak Mali[6], Tomáš Zikmund[1], Markéta Kaucká[7], Federica Papi[3], Jana Goyens[8], Anass Bouchnita[9,10], Andreas Hellander[9], Igor Adameyko[11] and Jozef Kaiser[1*]

[1] Central European Institute of Technology, Brno University of Technology, Czech Republic

[2] Elettra-Sincrotrone Trieste S.C.p.A., Basovizza, Trieste, Italy

[3] Speleovivarium Erwin Pichl, Adriatic Speleology Society, Trieste, Italy

[4] Institute Tular Cave Laboratory, Kranj, Slovenia

[5] Research Centre of the Slovenian Academy of Sciences and Arts: Karst Research Institute, Postojna, Slovenia

[6] University of Ljubljana, Biotechnical Faculty, Department of Biology, Ljubljana, Slovenia

[7] Max Planck Institute for Evolutionary Biology, Plon, Germany

[8] Laboratory of Functional Morphology, University of Antwerp, Belgium

[9] Department of Information Technology, Uppsala University, Sweden

[10] Department of Integrative Biology, University of Texas at Austin, USA

[11] Medical University of Vienna, Vienna, Austria

* Jozef.Kaiser@ceitec.vutbr.cz

ORCID iDs:

Jozef Kaiser [0000-0002-7397-125X]; Markéta Tesařová [0000-0002-5200-7365]; Lucia Mancini [0000-0003-2416-3464; Rok Kostanjšek [0000-0001-8377-1182]; Lilijana B Mali [0000-0001-7027-9535]; Tomáš Zikmund [0000-0003-2948-5198]; Markéta Kaucká [0000-0002-8781-9769]; Jana Goyens [0000-0003-0176-884X]; Anass Bouchnita [0000-0002-2729-6484]; Andreas Hellander [0000-0001-7273-7923]; Igor Adameyko [0000-0001-5471-0356];

Abstract

Background: The lightless caves can harbour a wide range of living organisms. Cave animals have evolved a set of morphological, physiological and behavioural adaptations known as troglomorphisms, enabling their survival in perpetual darkness, narrow temperature and humidity ranges, and nutrient scarcity of subterranean environment. In this study, we focused on adaptations of skull shape and sensory systems in blind cave salamander, *Proteus anguinus*, also known as olm or simply proteus - the largest cave tetrapod and the only European amphibian living exclusively in subterranean environments. This extraordinary amphibian compensates the loss of sight by enhanced non-visual sensory systems including mechanoreceptors, electroreceptors and chemoreceptors. We compared developmental stages of *Proteus anguinus* with *Ambystoma mexicanum,* also known as axolotl*,* to make an exemplary comparison between cave- and surface-dwelling paedomorphic salamanders.

Findings: We used contrast-enhanced X-ray computed microtomography for the 3D segmentation of the soft tissues in the head of *Proteus anguinus* and *Ambystoma mexicanum*. Sensory organs were visualised to understand how the animal is adapted to living in complete darkness. X-ray microCT datasets were provided along with 3D models for larval, juvenile and adult specimens, showing the cartilage of the chondrocranium, the position, shape and size of the brain, eyes and olfactory epithelium.

Conclusions: *Proteus anguinus* still keeps some of its secrets. Our high-resolution X-ray microCT scans together with 3D models of the anatomical structures in the head may help to understand the nature and

origin of the mechanisms behind its adaptations to the subterranean environment, which led to series of troglomorphies.

Keywords

Proteus anguinus, *Ambystoma mexicanum*, olm, axolotl, X-ray microCT, microtomography, salamander, cave animal, subterranean adaptations

Data Description

Context

Proteus anguinus, also known as the olm or simply proteus, has been under the attention of scientists and animal traders for centuries. *Proteus anguinus* is an apex predator of the karst underground waters. Its presence indicates a stability of food chains in the subterranean ecosystem. Its geographic distribution is limited to the Dinaric Karst; it ranges from the Gulf of Trieste in Italy, through the southern half of Slovenia, costal mainland of Croatia and parts of Bosnia and Hercegovina, as far as adjacent parts of Montenegro. With its extremely fragmented and limited habitat, *Proteus anguinus* is particularly vulnerable to pollution of groundwater. Some populations have been locally destroyed or endangered by pollution or habitat destruction [1, 2]. *Proteus anguinus* is an important object of research from at least two perspectives. First, *Proteus anguinus* has a historical role during the formation of the modern science from 17th to 19th century, puzzling the minds of most prominent early naturalists, from Valvasor to Linnaeus, Scopoli, Cuvier and Humboldt, and from Lamarck to Darwin and Goette. Second, *Proteus anguinus* has a potential to answer the questions of the science of today and future (e.g., regeneration, cave-related adaptations, enormous genome, and conservation of subterranean biodiversity) [3].

Proteus anguinus was first mentioned Janez Vajkard Valvasor in 1689. Its scientific name *Proteus anguinus* was given by Josephus Nicolaus Laurenti in 1768 and thus *Proteus anguinus* became the first taxonomically described cave animal in the world [4]. In the last two centuries, more detailed research has been performed on *Proteus anguinus*. Two of the central figures of the early *Proteus anguinus* research were Žiga

Zois, a naturalist from Ljubljana, who firstly described its behaviour and conducted earliest physiological and ecological observations. Then, Karl von Schreibers, a Viennese zoologist, who firstly explored the anatomy of *Proteus anguinus* [5]. This mysterious animal, which retains larval features at adult stage, started to interest the scientific community in the early 19th century, with a focus on its secretive mode of reproduction. In 1859, *Proteus anguinus* served as an example of blind cave animals in the famous monograph *On the Origin of Species,* where Charles Darwin attributed the reduction of eyes wholly to their disuse in darkness [6]. *Proteus anguinus* also became a model species in classical studies of comparative anatomy of the late 19th century. In the 20th century, more systematic research was enabled by overcoming the inaccessibility of *Proteus anguinus* subterranean habitat in captivity of cave laboratories worldwide, including France, Slovenia and Germany; the studies focused on cave-related physiology, ecology, behaviour and molecular phylogeny [3]. Fortunately, the attention to *Proteus anguinus* and its habitat has gradually received an important cultural and conservation attitude (1951 protected species in Slovenia, 1971 Ramsar convention, 1979 Bern convention and 1992 EU Habitat Directive). The year 2019 represented another milestone in the research of *Proteus anguinus*. The public presentation of the project of *Proteus anguinus* genome sequencing project [7] aimed to decipher the *Proteus anguinus* genetic information coded in the genome which is approximately 15-times larger than the human genome.

Obligate cave-dwellers often result in a set of specific morphological, physiological and behavioural traits (i.e., troglomorphism). Compared to their epigean relatives, cave-dwelling animals may have phylogenetically retained older sensory properties, improved them, or acquired new ones, enabling their survival in dark habitats [8]. *Proteus anguinus* evolved a range of adaptations such as the loss of pigmentation, slow metabolism, and capability of getting through extreme starvation. Moreover, the loss of eyesight is compensated by other specialized senses which enable to navigate in complete darkness. The mode of cave life and other biological peculiarities of *Proteus anguinus* and other troglobionts evoke the potential role of underwater audio-, mechano-, electro- and magnetoreception [9, 10].

Specimens of *Proteus anguinus* are valuable study material as they are protected and vulnerable. Our knowledge of *Proteus anguinus* larval and juvenile stages are still extremely scarce. For these reasons, we

aimed to use *Proteus anguinus* specimens from existing collections to avoid collecting them from nature. These animals died by natural causes and their bodies were preserved. We accessed the collection, applied non-destructive staining and a non-destructive imaging method X-ray computed microtomography (microCT) and then returned the specimens back to the collection. In this way, we obtained high-quality and high-resolution 3D data without a need for sacrificing any animal.

X-ray microCT has become a powerful method in developmental biology for exploring morphological changes in 3D. Geometric morphometrics based on X-ray microCT has already been used previously for an exploratory analysis of the morphology of the cranial osteology in the white and black subspecies of *Proteus anguinus* [11, 12]. However, without contrast-enhanced techniques, less dense tissues such as sensory organs would be unrecognisable or inaccurate due to an insufficient contrast for detailed 3D analysis. In our study, we use staining of soft tissues by phosphotungstic acid (PTA) or iodine in order to visualise soft tissues in the head of *Proteus anguinus*. For the first time, we look to volumetric internal structures by using a non-destructive imaging technique and we show the sensory organ with a high spatial and contrast resolution.

Proteus anguinus has been an important object of study in the history of international nature research, intriguing scientists. Thanks to studies in the last 300 years [5], mysteries of this cave amphibian are slowly being unravelled. However, *Proteus anguinus* still leaves gaps in our knowledge about its ecology, evolution and physiology. Our high-resolution microCT scans together with 3D models of anatomical structures in the head could help to understand the nature and origin of the mechanisms behind its adaptations to the subterranean lightless environment, which led to the acquisition of the troglomorphic features.

Methods

Sample preparation

The approval for the capture, handling, maintenance and breeding in captivity of the animals used in the study was granted by the Ministry of the Environment and Spatial Planning of the Republic of Slovenia, Slovenian Agency for the Environment (Permits nos. 35701-36/01, 35601-95/2009-4 and 35601-132/2014-

4), by the Italian Republic, Friuli Venezia Giulia Region (Permit nos. 4105/6MU4/95/04/12), and by the Italian Republic, Ministry of Ecological Transition (Permits nos. 3006/015590-93, 39/04). No animals were sacrificed for this study. Our experimental plan involved scanning *Proteus anguinus* and the axolotl *Ambystoma mexicanum* samples at different stages to study various parts of the skull and sensory organs.

All five *Proteus anguinus* specimens (NCBI:txid221568) were stored in 75% ethanol. Specimens of *Ambystoma mexicanum* (NCBI:txid8296) were reared at the Speleovivarium Erwin Pichl (Italy) since 2004 and included a 5-year-old adult female (died in 2009) with a total length of 194 mm, and a larva of 6 days old (died in 2012) with a total length of 11 mm. Specimens of *Proteus anguinus*: one larva, 3 weeks old (died in 2007), length 23 mm, captive breeding originating from the Postojna-Planina Cave System, from the collection of the Tular Cave Laboratory (Slovenia); one juvenile, 35 mm long, deceased at the collection site in a spring near Metković (Croatia), from the collection of the Department of Biology, Biotechnical Faculty, University of Ljubljana (Slovenia); one adult, sex unknown, length 276 mm, collected in Postojna-Planina Cave System (Slovenia, 1989), died in captivity of the Speleovivarium in 1999, from the collection of the Speleovivarium Erwin Pichl (Italy).

We modified a contrast-enhancing protocol initially developed by Brian Metscher [13] that has been successfully applied on salamander tissues before [14]. Larval samples of both *Proteus anguinus* and *Ambystoma mexicanum*, and juvenile *Proteus anguinus* were stained with 1% phosphotungstic acid (PTA) in 90% methanol for seven weeks. The solution was exchanged with fresh one once a week. The adult *Proteus anguinus* and *Ambystoma mexicanum* specimens were stained with 2% iodine (instead of PTA) in 90% methanol for six weeks to ensure that the contrasting agent would penetrated to the entire sample, because iodine penetrates better than PTA. Subsequently, the samples were gradually rehydrated in ethanol series (90%, 80%, 70% and 50%), one day for each concentration (i.e. 4 days of rehydration). The samples were then stabled in polyimide tubes filled with 1% low-melting agarose gel to prevent the sample movement during CT scan.

Image acquisition

The head region of the samples was scanned by using a laboratory X-ray microCT system GE Phoenix v|tome|x L 240 (GE Sensing & Inspection Technologies GmbH, Germany). The system was equipped with a 180 kV/15 W maximum power nanofocus X-ray tube and a high-contrast flat panel detector DXR250 with 2,048 × 2,048 pixels resolution and (200 × 200) μm^2 pixel size. 2,000 projections over a total scan angle of 360° were acquired with an exposure time of 900 ms per projection. Each projection was captured three times, and an average of the signal was used to improve the signal-to-noise ratio. The acceleration voltage of the X-ray tube was set to 60 kV and the tube current to 200 µA for larval and juvenile *Proteus anguinus*; 80 kV and 250 µA were used, respectively, for voltage and current for adult samples. The X-ray beams of lower energies were filtered with a 0.2 mm-thick aluminium plate for larval and with a 1 mm-thick aluminium plate for adult samples. The voxel sizes of the reconstructed data were the followings: 3.5 µm for juvenile *Proteus anguinus*, 5.8 µm for larval *Proteus anguinus*, 25 µm for adult *Proteus anguinus*, 2.8 µm for larval *Ambystoma mexicanum* and 27.5 µm for adult *Ambystoma mexicanum*.

Tomographic data processing

The tomographic reconstruction was performed using GE phoenix datos|x 2.0 software (GE Sensing & Inspection Technologies GmbH, Germany, RRID:SCR_017996). A segmentation procedure was then applied to reconstructed slices. The Avizo 7.1 (Thermo Fisher Scientific, USA, RRID:SCR_014431) image processing software was used for semi-automatic segmentation [15-17] of structures in the head. To reduce the load of 3D segmentation volume, every 3rd slice was manually segmented, and the rest was calculated by linear interpolation between manually segmented slices [17]. We converted the semi-manually segmented models into polygonal meshes and imported this in VG Studio MAX 2.2 software (Volume Graphics GmbH, Germany , RRID:SCR_017997) for 3D visualisations.

Data validation and quality control

By contrast-enhanced X-ray microCT scan, we were able to visualise the internal structures of *Proteus anguinus* head. Figure 1 shows the manually segmented cartilaginous chondrocranium as well as the position and the shape of the brain, the remnant eyes and the olfactory epithelium. A considerable portion of the cranial skeleton in the adult *Proteus anguinus* specimen remains cartilaginous.

A validation of the semi-automatic segmentation procedure is presented in Figure 2. The 3D models were created by an operator based on the grey-scale value contrast and the shape of the structures. The detailed procedure was described in the methods section and followed our previous study [17].

In Figure 3, we compared the microCT datasets and segmentations of the internal head structures of the larval and adult specimens of both *Proteus anguinus* and *Ambystoma mexicanum*. The head of the troglobiotic *Proteus anguinus* is narrower and more elongated in comparison with the epigean *Ambystoma mexicanum* which lives in open surface waterbody but not underwater caves. Our 3D segmentations show remnant eyes at larval and juvenile *Proteus anguinus*, but no remnant of eyes was noticed in the adult specimen. The progressive degeneration of the eye in the development of the *Proteus anguinus* may lead to the apparent disappearance of the eye in the adult animal [18]. By contrast, 3D models of *Ambystoma mexicanum* clearly show the eyes together with an optic nerve that leads to the brain in both the larval and adult stages. However, the absence of remnant eyes and optic nerves in our data can be caused by low contrast of these structures in microCT data as the segmentation was done manually by the operator and based on their grey-scale values and their shape.

Re-use potential

Museum-type documentation of rare and endangered species

The presented datasets give insight not only to developmental biology of *Proteus anguinus*. Together with 3D datasets of *Ambystoma mexicanum*, they are important study materials for the conservation efforts to preserve these endangered amphibians. According to the global assessment of the International Union for Conservation of Nature (IUCN), 43% of amphibian species are in decline while 32% are threatened with

extinction [19], *Proteus anguinus* is currently classified as vulnerable. The X-ray microCT method enables anatomical studies without damaging the morphology of the specimens and is therefore exceptionally appropriate for studying endangered species with limited amount of available specimens. Semi-automatically segmented images and the extracted 3D models could also be taken as an input into a machine learning algorithm. The field of image processing is becoming dominated by deep learning algorithms and convolutional neural networks [20]. Creating an online database could be also beneficial for student studies and distant learning. Especially, last year showed the importance of an easy access to online study materials because of Covid-19 restrictions.

Perspectives: Cellular resolution

Using microCT scan with a conventional X-ray source, we obtained data of excellent quality which depict single cells in the cartilaginous elements (Figure 4). Despite the fact that the cells can be visually detected, their automatic segmentation and quantification is further challenging. The potential of X-ray microCT imaging with synchrotron sources for the study of 3D-cell distribution was demonstrated in our previous study on salamander limbs [12] and the potential for biomedical applications was shown before [21, 22]. The data with cellular resolution can be used as the input for the study of polarization of cells in the extracellular matrix in salamander limbs or for mathematical modelling of joint formation [14].

Research outlook

The morphology of the cranium carries important information related to mechanics involved in feeding, as well as competitive, reproductive, and anti-predatory behaviour. Even small differences in cranial skeleton may have important biomechanical and ecological implications [23]. The most detailed description of the *Proteus anguinus* skull are those of Dolivo-Dobrovolsky [24, 25], Ivanović et al. [11], Papi et al. [12] and Bizjak Mali & Sket [26]. MicroCT contrast-enhanced data included overall 3D information and a further segmentation. Working on the provided data can be taken as an input for various type of analyses (Figure 5). The visualisation of muscles of upper and lower jaw could be used for biting analyses that may shed light on the predatory abilities of *Proteus anguinus*.

In general, our datasets will help to investigate how evolutionary changes of the head in shape and its integrated muscle-skeletal apparatus, nervous system and sensory organs would help for troglomorphic adaptation. Because *Proteus anguinus* is a cave-dwelling animal, the craniofacial design underwent a number of cave-related evolutionary adaptations, acquiring protracted and longitudinally-elongated skeletal elements, in comparison to an epigean *Ambystoma mexicanum*, subjected in the comparative microCT scanning analysis and in-depth 3D analysis. Indeed, when compared to *Proteus anguinus*, *Ambystoma mexicanum* exhibits a wider skull with a massive jaw. Elongated skull, body and limbs are common features of troglomorphism for organisms inhabiting subterranean habitats [27, 28]. Only 14 cave-obligate species are known (all plethodontid and proteid salamanders), but this is likely to be an underestimate number because of cryptic species [29]. In regard, results of our study provide insight in evolutionary trends in adaptive morphological traits as a result of convergent evolution across phyla. Even without evolutionary comparison across phyla, our results enable further studies on evolution of the skeletal parts in the adaptive landscapes individually for closely related species.

Not only evolutionary questions but also developmental patterns can be tackled with these data. For instance, to investigate if the elongated skull shape develops evenly with the growth of the animal, we investigated the larval *Proteus anguinus*. The larva of *Proteus anguinus*, although being small, revealed their miniaturized protracted chondrocrania with similar spatial proportions to the adult form. Thus, the elongated body and stretched cranium may indicate possible benefits at any phase of development. Such comparative developmental and growth-related studies open up new opportunities to look into the dynamics of the skeletal shape development in relation to the specifics of the environment.

However, when it comes to the adaptations in sensory organs, the 3D-analysis of the head revealed major differences in visual and olfactory systems of *Proteus anguinus* and *Ambystoma mexicanum*. Firstly, *Proteus anguinus* is blind, a typical troglomorphic trait which is explained by the troglobiotic way of living. The eye development in *Proteus anguinus* larva begins as in other amphibians. The regression of almost normally formed eyes starts soon after hatching and gradually leads to a considerable reduction of the eyes in adult *Proteus anguinus* [18], while surface-dwelling salamanders develop fully functional eyes. The morphological

comparisons of the *Proteus anguinus* developmental stages with their gradual reduction of eyes may improve our knowledge on the mechanisms of eye degeneration in *Proteus anguinus* and possibly other similar blind cave-dwellers. Moreover, the eyeballs develop in a complex conjunction with muscular apparatus [30] and optical nerve. Here, in *Proteus anguinus*, it is possible to question how the induction and degeneration of auxiliary tissues is achieved during the degradation of the pre-shaped eyeballs.

The animals with eye-regression are generally known to inhabit caves, therefore the vision is nearly useless. The blind Mexican cave fish (*Astyanax mexicanus*) is one of the popular model systems to study the loss of vision and developmental arrest of the visual system [31]. However, the blind fish and the other models, including *Proteus anguinus*, still have a great potential for comparison with other eyeless vertebrates [32]. Therefore, our results and datasets may enable new insights into the evolutionary trends in the eye development and degeneration strategies in cave-dwellers across vertebrate clades.

The olfaction plays an important role in the life of the salamanders [8]. The analysis of 3D-rendered olfactory organs revealed striking differences between the surface-dwelling *Ambystoma mexicanum* and the cave-dwelling *Proteus anguinus*. Elongated and tube-shaped olfactory cavities in *Proteus anguinus* likely emerge as another adaptation to the cave environment, where enhanced olfaction capabilities pose an advantage in the absence of visual signals [33]. Comparing to *Ambystoma mexicanum*, elongated olfactory cavities of *Proteus anguinus* might enable higher dynamic range of sensitivity, due to a more efficient longitudinal diffusion of signals upon the entry via nostril openings. In line with this, the olfactory nerves of *Proteus anguinus* are also considerably elongated, which is explained by a rather long rostral part of the skull in *Proteus anguinus*. Therefore, the studies of evolutionary divergence of the olfactory system, its sensitivity and general design, could benefit from the deposited datasets.

Finally, salamanders represent a well-established model system for the research of regeneration, and the fundamental principles of multi-tissue regeneration have been already revealed [34]. Regeneration of *Proteus anguinus* has been described previously, yet the provided data offer an important insight into the evolutionary differences in regeneration among salamanders with fundamentally different lifestyles.

Data Availability

The data sets supporting the results of this article are available in the *GigaScience* Database repository [35]. We provide reconstructed slices as DICOM image stacks and segmented structures in Standard Triangle Language (STL) format for the head region of three specimens (a larva, a juvenile and an adult) of *Proteus anguinus* and two specimens (a larva and an adult) of *Ambystoma mexicanum*. For segmented structures, we also provide segmented masks as DICOM image stacks – one stack for each structure. The folders are structured so that each folder represents one sample containing a folder with DICOM stack, a folder with STL files and a folder with segmented masks. DICOM image stack can be opened in any image viewer supporting this format, we recommend ImageJ for viewing the data [36]. To explore datasets in 3D, specialized free viewers are available - Drishti [37], DragonFly (Object Research Systems ORS Inc, Montreal, Canada) or others. For analysis and further segmentation, we recommend the use of ITK-SNAP [38] or commercial software, e.g., Avizo (Thermo Fisher Scientific, USA) or VG Studio MAX (Volume Graphics GmbH, Germany). A detailed description and a manual for segmentation of biological data can be found in our previous works [17, 39]. The STL files can be also explored in 3D mesh viewers; popular free open-source software, e.g., MeshLab [40] or Blender [41] as well as in the sketchfab repository [42]..

Figure titles and legends

Figure 1: 3D reconstructions of *Proteus anguinus* head based on X-ray microCT data. Larva (top), juvenile (middle) and adult (bottom) *Proteus anguinus*. Images in the first column show semi-transparent 3D renderings of the head with skin in dorsal view. Dorsal, lateral and frontal views of the segmented and color-coded internal soft structures are shown in the second to the fourth column.

Figure 2: Accuracy validation of semi-automatic segmentations of X-ray microCT data in juvenile *Proteus anguinus*. Raw CT image (left) and the corresponding segmented image (middle) through the transverse plane (in green) of the head of a juvenile *Proteus anguinus*(right).

Figure 3: Comparison of the head and internal soft tissue anatomy of the cave-dwelling *Proteus anguinus*with *Ambystoma mexicanum* in larval and adult specimens. Images in the first row show 3D renderings of the head with skin in dorsal view. The second row shows color-coded segmented brain, cartilage, bones, eyes with optic nerve and olfactory epithelium. The third row shows these structures without bone and cartilage for better clarity.

Figure 4: Images at near cellular-level resolution showing the cartilaginous elements in juvenile *Proteus anguinus* obtained by microCT. The white dots represent cell nuclei. 3D detail of the cartilaginous 1st basibranchial element of the hyobranchial apparatus in ventral view (yellow colour; top row) with three orthogonal CT slices along the frontal, sagittal and transverse plans (second row).

Figure 5: Potential use of contrast-enhanced X-ray microCT data. Segmentation of craniofacial muscles and ear labyrinth in *Ambystoma mexicanum* and *Proteus anguinus.*

Declarations

List of abbreviations

MicroCT - X-ray computed microtomography; PTA – phosphotungstic acid; STL – Standard Triangle Language, 3D – three-dimensional

Consent for publication

Not applicable.

Competing interests

The authors declare no competing interests.

Funding

We acknowledge CzechNanoLab Research Infrastructure supported by MEYS CR (LM2018110). MT acknowledges grant CEITEC VUT-J-21-7145, the Brno City Municipality as a Brno Ph.D. Talent Scholarship Holder and Martina Roeselova Memorial Fellowship. JG was funded the Fonds Wetenschappelijk Onderzoek

– Vlaanderen by a postdoctoral fellowship (Grant No. 12R5118N). We thank Teo Delić for collecting the juvenile *Proteus anguinus* specimen.

Authors' contributions

M.T.: Writing – original draft, Visualization; L.M.: Conceptualization, Methodology; E.M.: Investigation, Writing – original draft; G.A.: Investigation, Writing – original draft; M.A.: Investigation, Writing – original draft; R.K.: Validation, Writing – review & editing; L.B-M.: Validation; T.Z.: Project administration; M.K.: Methodology; Writing – review & editing; F.P.: Methodology; J.G.: Data curation, Writing – review & editing; A.B.: Data curation; A.H.: Project administration; I.A.: Conceptualization, Project administration; J.K.: Funding acquisition , Supervision

References

1. Sket, B. (1997). Distribution of Proteus (Amphibia: Urodela: Proteidae) and its possible explanation. – *Journal of Biogeography.* 1997. 24:263–280.

2. Gorički, Š., Stanković, D., Snoj, A., Kuntner, M., Jeffery, W.R., Trontelj, P., Pavićević, M., Grizelj, Z., Năpăruş-Aljančič, M., Aljančič, G. Environmental DNA in subterranean biology: Range extension and taxonomic implications for Proteus. *Sci Rep.* 2017. doi:10.1038/srep45054

3. Aljančič, G. History of research on Proteus anguinus Laurenti 1768 in Slovenia. *Folia biologica et geologica***.** 2019. doi:10.3986/fbg0050

4. Laurenti, J. Synopsis Reptilium. *J.T. de Trattnern, Viennae.* 1768. 35-36.

5. von Schreibers, K. A. historical and anatomical description of a doubtful amphibious animal of Germany, called, by Laurenti, Proteus anguinus. *Philosophical Transactions.* 1801. 91:241–264.

6. Charles, D. On the Origin of Species. *John Murray* (1859).

7. Kostanjšek, R., Diderichsen, B.,Recknagel, H., Gunde-Cimerman, N., Gostinčar, C., Fan, G., Kordiš, D., Trontelj, P., Jiang, H., Bolund, L., Luo, Y.. Toward the massive genome of *Proteus anguinus*— illuminating longevity, regeneration, convergent evolution, and metabolic disorders. Ann. N.Y. Acad. Sci. 2021. doi:10.1111/nyas.14686

8. Schlegel, P.A., Steinfartz, S. & Bulog, B. Non-visual sensory physiology and magnetic orientation in the Blind Cave Salamander, *Proteus anguinus* (and some other cave-dwelling urodele species). Review and new results on light-sensitivity and non-visual orientation in subterranean urodeles (Amphibia). *Animal Biology.* 2009. 59:351-384.

9. Bulog, B. & Schlegel, P. Functional morphology of the inner ear and underwater audiograms of *Proteus anguinus* (Amphibia, Urodela). *Pflügers Archiv - European Journal of Physiology.* 2000. 439:165-167.

10. Hervant, F., Mathieu, J. & Durand, J. Behavioural, physiological and metabolic responses to long-term starvation and refeeding in a blind cave-dwelling (*Proteus anguinus*) and a surface-dwelling (*Euproctus asper*) salamander. *Journal of Experimental Biology.* 2001. 204:269-281.

11. Ivanović, A., Aljančič, G., Arntzen, J.W. & van Rooijen, J. Skull shape differentiation of black and white olms (*Proteus anguinus anguinus* and *Proteus a. parkelj*): an exploratory analysis with micro-CT scanning. *Contributions to Zoology.* 2013. 82:107-114.

12. Papi, F., Mauri, E., Pesaro, S., Tromba, G. & Mancini, L. Analysis of the skull of *Proteus anguinus* anguinus by high-resolution X-ray computed microtomography. *Natura Sloveniae.* 2018. 2:43-45.

13. Metscher, B. MicroCT for comparative morphology: simple staining methods allow high-contrast 3D imaging of diverse non-mineralized animal tissues. *BMC Physiol. 2009.* doi: 10.1186/1472-6793-9-11

14. Tesařová, M., Mancini, L., Simon, A., Adameyko, I., Kaucká, M., Elewa, A., Lanzafame, G., Zhang, Y., Kalasová, D., Szarowská, B., Zikmund, T., Novotná, M. & Kaiser, J. A quantitative analysis of 3D-cell distribution in regenerating muscle-skeletal system with synchrotron X-ray computed microtomography.

Sci Rep. 2018. doi:10.1038/s41598-018-32459-2

15. Bayle, P., Braga, J., Mazurier, A. & Macchiarelli, R. Dental developmental pattern of the Neanderthal child from Roc de Marsal: a high-resolution 3D analysis. *Journal of Human Evolution.* 2009. 56:66-75.

16. Zanolli, C., Bondioli, L., Mancini, L., Mazurier, A., Widianto, H. & Macchiarelli, R. Brief communication: Two human fossil deciduous molars from the sangiran dome (Java, Indonesia). *American Journal of Physical Anthropology.* 2012. 147:472-481.

17. Tesařová, M., Zikmund, T., Kaucká, M., Adameyko, I., Jaroš, J., Paloušek, D., Škaroupka, D. & Kaiser, J. Use of micro computed-tomography and 3D printing for reverse engineering of mouse embryo nasal capsule. *J Instrum.* 2016. doi:10.1088/1748-0221/11/03/C03006

18. Durand, J. Ocular development and involution in the european cave salamander, *Proteus anguinus* laurenti. *The Biological Bulletin.* 1976. 151:450-466.

19. Stuart, S.N., Chanson, J.S., Cox, N.A., Young, B.E., Rodrigues, A.S.L., Fischman, D.L. & Waller, R.W. Status and trends of amphibian declines and extinctions worldwide. *Science***.** 2004. doi:10.1126/science.1103538.

20. Ronneberger, O., Fischer, P. & Brox, T. U-Net: Convolutional Networks for Biomedical Image Segmentation. in *Medical Image Computing and Computer-Assisted Intervention – MICCAI 2015* Navab. 2015. doi:10.1007/978-3-319-24574-4_28.

21. Tromba, G., Longo, R., Abrami, A., Arfelli, F., Astolfo, A., Bregant, B., Brun, F., Casarin, K., Chenda, V., Dreossi, D., Hola, M., Kaiser, J., Mancini, L., Menk, R.H., Quai, E., Quaia, E., Rigon, L., Rokvic, T., Sodini, N., Sanabor, D., Schultke, E., Tonutti, M., Vascotto, A., Zanconati, F., Cova M., & Castelli, E. The SYRMEP Beamline of Elettra: Clinical Mammography and Bio-medical Applications. *AIP Conf. Proc.* 2010. 1266:

18-23.

22. Du Plesis, A., Broeckhoven C., Guelpa A., Gerhard le Roux S. Laboratory x-ray micro-computed tomography: a user guideline for biological samples. *Gigascience*. 2017. doi:10.1093/gigascience/gix027

23. van der Meij, M. & Bout, R. The relationship between shape of the skull and bite force in finches. *Journal of Experimental Biology.* 2008. 211:1668-1680.

24. Dolivo-Dobrovolsky, V. Das Kopfskelett des Grottenolmes (*Proteus anguinus* Laur.). *Zoologischer Anzeiger. 1923;* 57:281-284.

25. Dolivo-Dobrovolsky, V. Lobanja človeške ribice (Proteus anguinus Laurenti). *Rad Jugoslavenske akademije zna-nosti i umjetnost. 1926;* 232:190-209.

26. Bizjak Mali, L. & Sket, B. History and biology of the «black proteus« (*Proteus anguinus parkelj* Sket & Arntzen 1994; Amphibia: Proteidae). *Folia biologica et geologica.* 2019; 60: 5-37.

27. Culver, D. & Pipan, T. *The Biology of Caves and Other Subterranean Habitats*. Oxford University Press. 2019. doi:10.1093/oso/9780198820765.001.0001.

28. Ribera, I., Cieslak, A., Faille, A. & Fresneda, J. Historical and Ecological Factors Determining Cave Diversity: Analysis and synthesis. *Cave Ecology*. 2018. doi:10.1007/978-3-319-98852-8_10.

29. Recknagel, H., Trontelj, P. 2021. From Cave Dragons to Genomics: Advancements in the Study of Subterranean Tetrapods. *BioScience*. 2021. doi: 10.1093/biosci/biab117

30. Comai, G., Tesařová, M., Dupé, V., Rhinn, M., Vallecillo-García, P., da Silva, F., Feret, B., Exelby, K., Dollé, P., Carlsson, L., Pryce, B., Spitz, F., Stricker, S., Zikmund, T., Kaiser, J., Briscoe, J., Schedl, A., Ghyselinck, N., Schweitzer, R., Tajbakhsh, S. & Hughes, S. Local retinoic acid signaling directs emergence of the extraocular muscle functional unit. *PLOS Biology.* 2020. doi: 10.1371/journal.pbio.3000902

31. Jeffery, W.R. Emerging model systems in evo-devo: cavefish and microevolution of development. *Evolution & development.* 2008, 10:265-272.

32. Tovar, R., Cantu, V., Fremaux, B., Gonzalez Jr, P., Spikes, A. & García, D. Comparative development and ocular histology between epigean and subterranean salamanders (Eurycea) from central Texas. *PeerJ.* 2021. doi: 10.7717/peerj.11840

33. Dumas, P., Chris, B. The olfaction in *Proteus anguinus*: a behavioural and cytological study. *Behavioural Processes.* 1998. 43:107–113.

34. Kumar, A., Godwin, J., Gates, P., Garza-Garcia, A. & Brockes, J. Molecular Basis for the Nerve Dependence of Limb Regeneration in an Adult Vertebrate. *Science. 2007.* 318:772-777.

35. Tesarova M; Mancini L; Mauri E; Aljančič G; Năpăruş-Aljančič M; Kostanjšek R; Mali LB; Zikmund T; Kaucka M; Papi F; Goyens J; Bouchnita A; Hellander A; Adameyko I; Kaiser J (2022): Supporting data for "Living in darkness: Exploring adaptation of Proteus anguinus in 3D by X-ray imaging" GigaScience Database. http://dx.doi.org/10.5524/102196

36. Schneider, C., Rasband, W. & Eliceiri, K. NIH Image to ImageJ: 25 years of image analysis. *Nature Methods.* 2012. 9:671-675.

37. Limaye, A. & Stock, S. Drishti: a volume exploration and presentation tool. doi:10.1117/12.935640.

38. Yushkevich, P.A., Gao, Y. & Gerig, G. ITK-SNAP: An interactive tool for semi-automatic segmentation of multi-modality biomedical images. in *2016 38th Annual International Conference of the IEEE Engineering in Medicine and Biology Society.* 2016. doi:10.1109/EMBC.2016.7591443.

39. Tesařová, M., Heude, E., Comai, G., Zikmund, T., Kaucká, M., Adameyko, I., Tajbakhsh, S. & Kaiser, J. An interactive and intuitive visualisation method for X-ray computed tomography data of biological samples in 3D Portable Document Format. *Sci Rep.* 2019*. doi: 10.1038/s41598-019-51180-2*

40. Cignoni, P., Callieri, M., Corsini, M., Dellepiane, M., Ganovelli, F. & Ranzuglia, G. MeshLab: an Open-Source Mesh Processing Tool. 2008.

41. Hess, R. *The essential Blender: guide to 3D creation with the open source suite Blender*. No Starch Press. 2007.

42. Salamander 3D Model Collection. Sketchfab. 2022 https://sketchfab.com/GigaDB/collections/salamander

Figure 1

Click here to access/download;Figure;Tesarova_Proteus_Figure1_rev.png

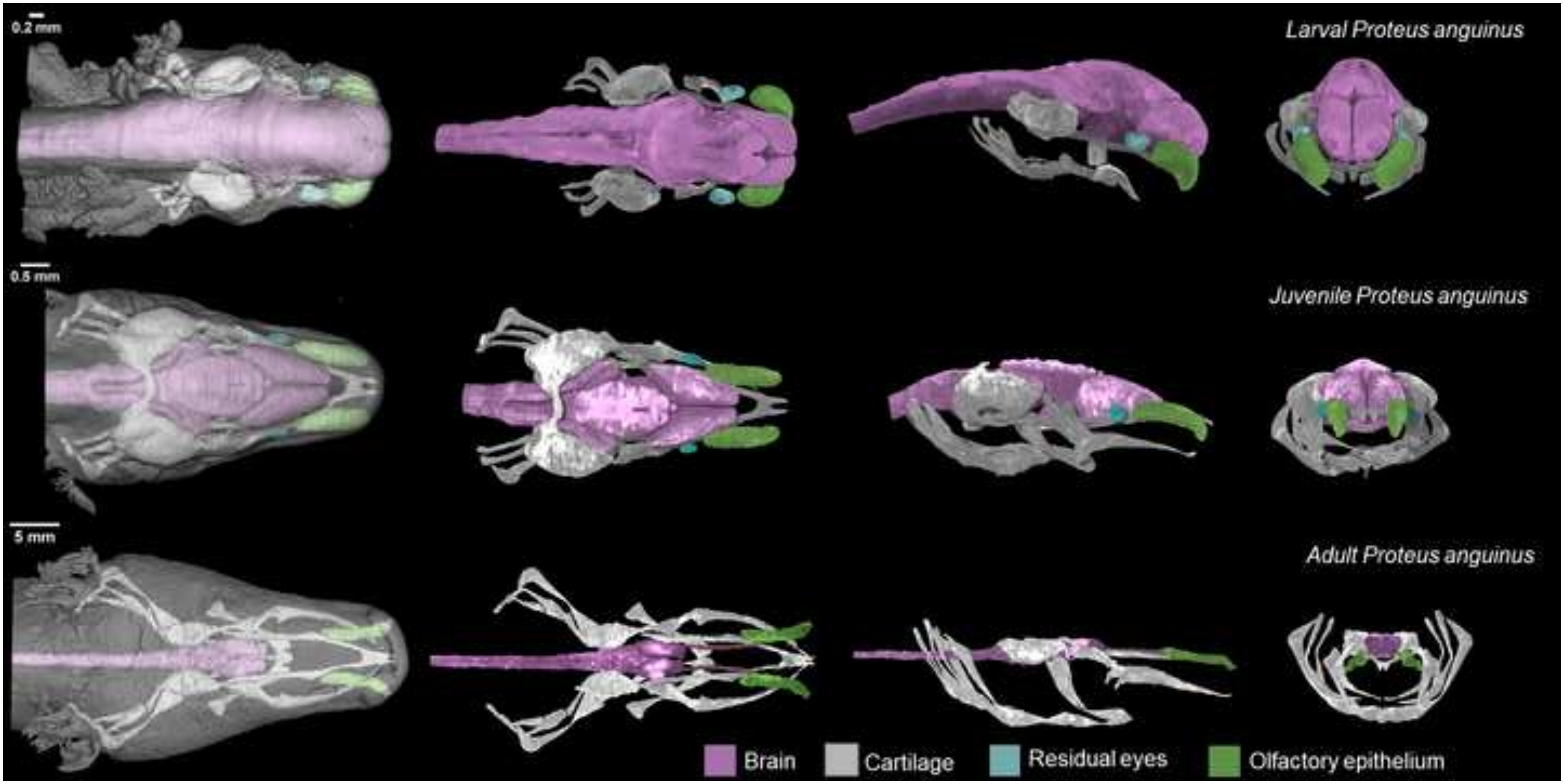

Figure 2 Click here to access/download;Figure;Tesarova_Proteus_Figure2_rev.png

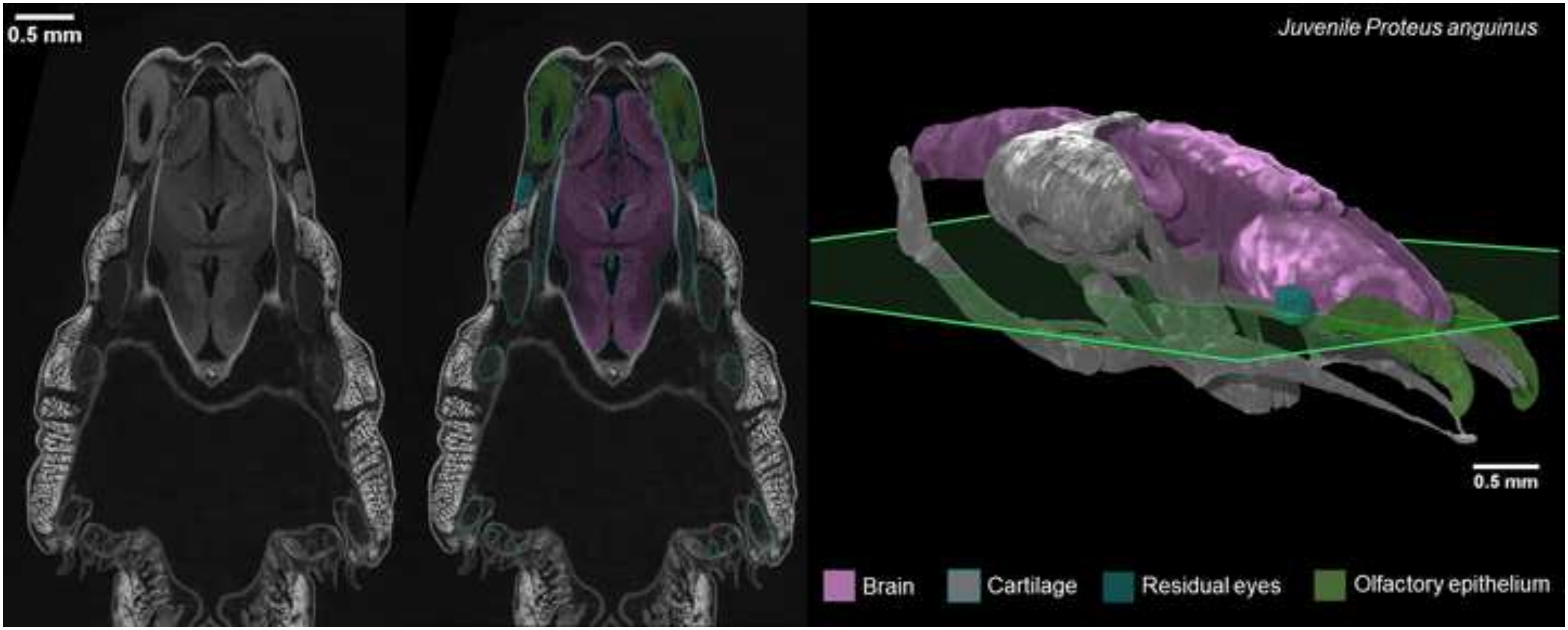

Figure 3

Click here to access/download;Figure;Tesarova_Proteus_Figure3_rev.png

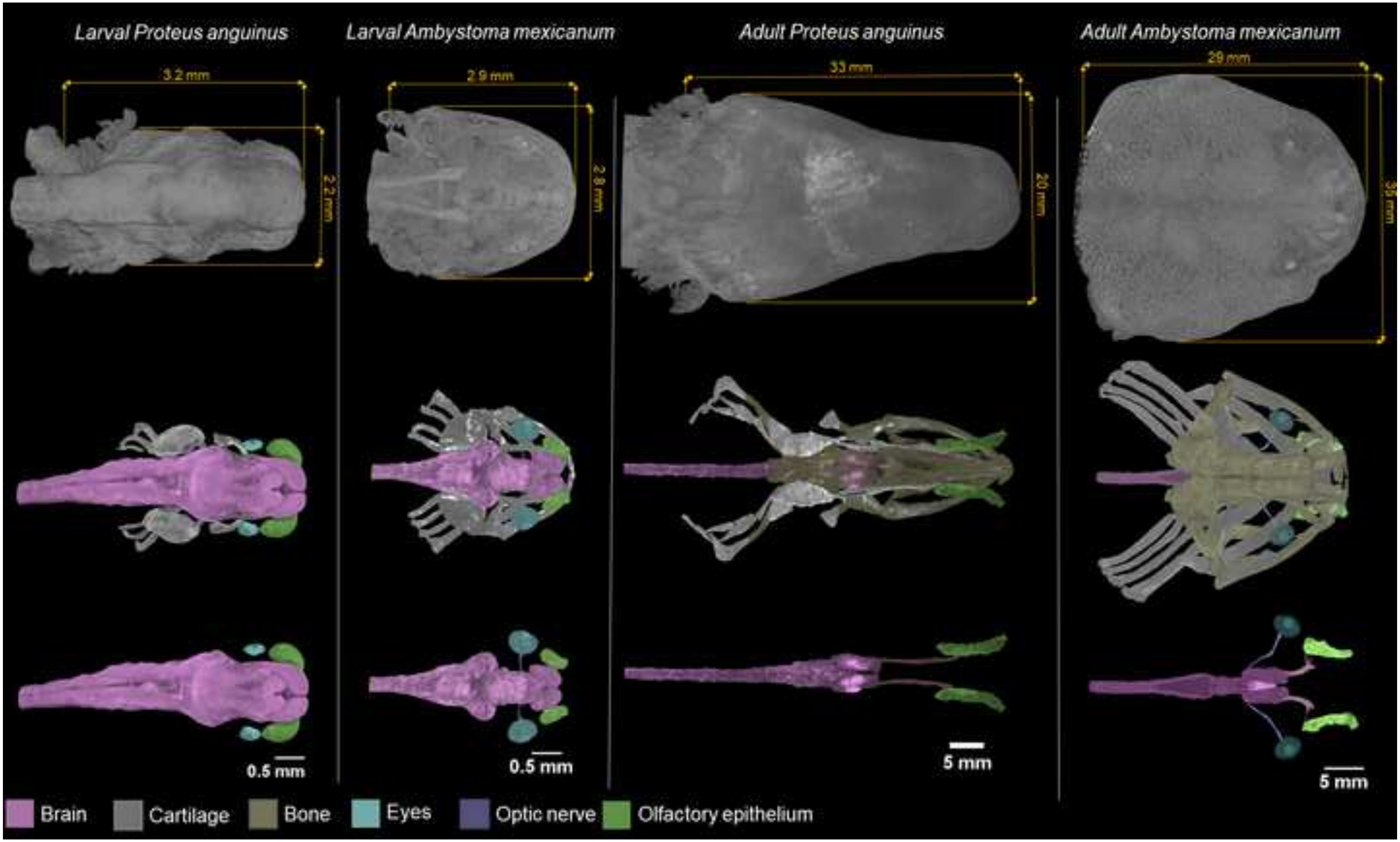

Figure 4

Click here to access/download;Figure;Tesarova_Proteus_Figure4_rev.png

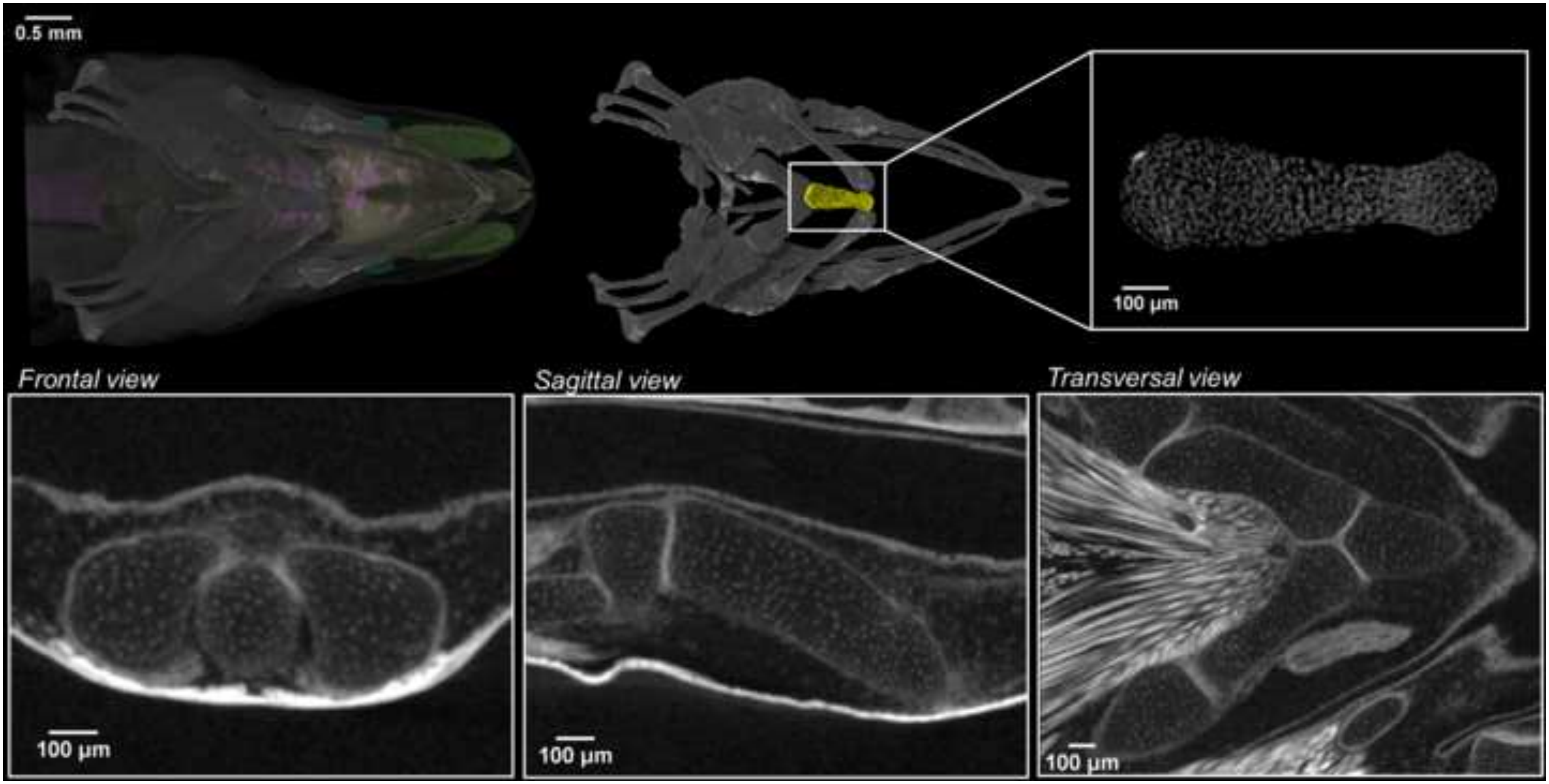

Figure 5

Click here to access/download;Figure;Tesarova_Proteus_Figure5_rev.png

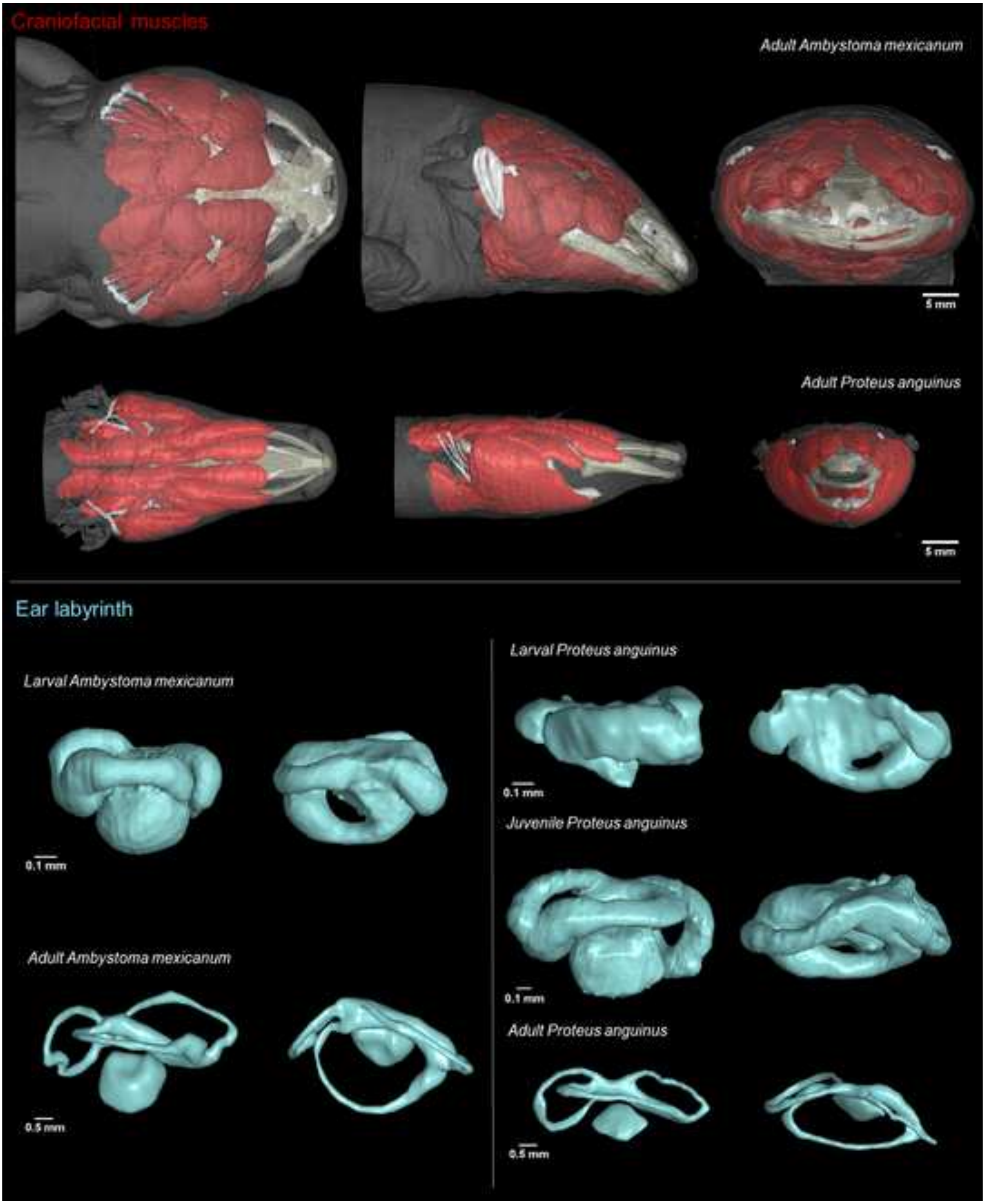

Supplement: giac030_GIGA-D-21-00259_Revision_1 [file giac030_giga-d-21-00259_revision_1.pdf]
