# Supplementary material for: Living in darkness: Exploring adaptation of Proteus anguinus in 3 dimensions by X-ray imaging
Source: Gigascience. 2022 Apr 5;11:giac030. doi: 10.1093/gigascience/giac030 (PMC8982192; doi:10.1093/gigascience/giac030)
Supplement: giac030_Response_to_Reviewer_Comments_Revision_1 [file giac030_response_to_reviewer_comments_revision_1.pdf]

Author's Response To Reviewer Comments

Close

Dear Editor,
Thank you for the possibility to revise our manuscript "Living in darkness: Exploring adaptation of Proteus anguinus in 3D by X-ray imaging". We went through the reviewers' comments and tried to do our best in order to improve the manuscript for the community of biologists as well as for imaging experts. We did new segmentation of craniofacial muscles and ear labyrinths which can be found in new Figure 5. We also took in account comments regarding more detailed datasets in the repository and we created STL files of the surface for the all 5 samples and we also added binary masks for each segmented structure. We are also thankful reviewers for their comments regarding the writing style and the manuscript was sent to the language check. Below, you can find our point-by-point response to reviewers' comments.
We hope this revision is suitable for publication GigaScience as a Data Note.

Yours Sincerely,
Prof. Jozef Kaiser
Research Group Leader
Advanced Instrumentation and Methods for Materials Characterization

tel: +420 541149700, +420 731141281
e-mail: jozef.kaiser@ceitec.vutbr.cz
web: www.ceitec.eu

Reviewer reports:

Reviewer #1: The blind white olm (Proteus anguinus anguinus) are unique in many aspects of its biology, including geographic distribution, development and particularly its salient troglomorphism, which resulted from its morphological adaptation to underwater cave-dwelling lifestyle. As mentioned in this technical study, our knowledge on this particular salamander species was accumulated bit by bit over the past few hundred years. Now we are standing at a point where details of morphological and developmental changes of this taxon can be better revealed by non-destructive methods based on more readily available specimens raised in labs due to the advancement of high-resolution microCT scan and contrast enhancing techniques. Sharing of high-resolution CT images of endangered species that are significant to advance our understandings of certain scientific questions should always be encouraged.

This manuscript authored by Tesařová et al. provided a dataset of contrast-enhanced microCT images for the head region of three specimens (a larva, a juvenile and an adult) of Proteus anguinus anguinus and also images for two specimens (a larva and an adult) of Ambystoma mexicanum for the purpose of comparative studies. Besides many annotations I made in the word file as attached, here are the rest of my other concerns:

Response:
Dear Reviewer,
Thank you for your response and all the comments regarding our manuscript. We considered annotations in the word document and we think it improved a manuscript, so we are really grateful for that! Below, you can find response for the rest of your concerns below:

1) The comparison between the olm and the axolotl is reasonable considering both species are neotenic and one is cave dwelling and the other is surface dwelling, and therefore avoid introducing noises from changes created by metamorphosis; however, why not choose any neotenic species in the sister group genus of Proteus, Necturus, for comparison, considering their phylogenetic closeness and disparate lifestyles (Necturus is surface dwelling in slow-moving streams)?

Response: This is a very interesting comment and we thank you for this perspective. It is true that comparison with neotenic species in the sister group genus of Proteus could give interesting data. We

decided for analyses of Ambystoma mexicanum for its wide use in developmental and regenerative studies. Also, Proteus anguinus and Ambystoma mexicanum share neoteny as a prominent attribute and have similar characteristics in regard to predation, aquatic life and reproduction. Proteus anguinus is currently the only neotenic genus living on the European continent. It is represented by two subspecies: Proteus anguinus anguinus (Laurenti 1768), strictly troglomorphic, and pigmeted subspecies Proteus anguinus parkelj (Skeet & Arntzen, 1994) of less-troglomorphic morphology. In the past, this was very different. The fossil record between the Late Cretaceous and the thermal maximum of Paleocene-Eocene revealed the presence of a large number of caudates in Europe including Cryptobranchidids, Batrachosauroidids and Ambystomidids (Skutschas & Gubin 2012). Many specimens with external gills and neotenic features were found. Therefore, the ancestors of Ambistomidae and Proteidae lived on the European continent about 50 Ma years ago sharing environments favorable to the amphibians spread (Vasilyan & Yanenko 2020). We were then motivated to compare specimens of Proteus and Ambystoma belonging to two families that in ancestral times shared marshy environments on the European continent. We have reason to believe that such a comparison may reveal similarities and / or differences on the morphologies examined which would otherwise be difficult to detect. However, consider other species in our future studies.

2) After checking the dataset by loading dicom files into software VG Studio, it seems that the larval specimen of the olm has some shrink in the head region, which may be unavoidable, but I would appreciate if you can enrich the descriptions for the contrast-enhancing experimental procedures by providing the length of the rehydration process for each specimen.

Response: Thank you for this remark. We added the following information to the method section: Subsequently, the samples were gradually rehydrated in ethanol series (90%, 80%, 70% and 50%), one day for each concentration (i.e. 4 days of rehydration).

3) Most of the datasets contain a complete head region except the one for the adult specimen of the axolotl, which has the posterior part of the hyobranchial apparatus missing. It is highly recommended to provide a more complete dataset for the axolotl adult specimen.

Response: We substituted dataset with more complete dataset for adult axolotl. As we focused on the segmentation of structures in the head, we chose only head area and did not realize that hyobranchial apparatus is missing. Now, there is a new DICOM series for adult axolotl containing also neck part.

4) The dataset for the adult specimen of the olm can not be properly loaded in visualization softwares like VG Studio and Photoshop, because it has three damaged dicom files, i.e., "Proteus_anguinus_adult_0661.dcm", "Proteus_anguinus_adult_2023.dcm" and "Proteus_anguinus_adult_2349". The first image is 590 kb, and the latter two are 0 kb in size, in contrast to most other images in the same dataset which are 637 kb. It's also noticeable that 233 images ranging from "Proteus_anguinus_adult_2116.dcm" to "Proteus_anguinus_adult_2348.dcm" are 2317 kb, and can not be properly loaded into software with files of 637 kb.

Response: Thank you for drawing our attention to this technical error that must have happened during the preparation of the data. We substitute the DICOM folder with corrected files and now the dataset is complete.

Generally speaking, the manuscript is clearly written, and the dataset is easily accessible and well controlled. I look forward to its formal publication. Feel free to contact me (jia.jia@ucalgary.ca) directly if any of my comments are unclear.

Best wishes,
Jia
October 8, 2021

Response: Dear Jia, thank you again for your time spent on our manuscript and for the all helpful comments and notices. If you feel there is still space for improvement, we still be happy to hear your comments.
With best wishes
Jozef

Reviewer #2: The authors have studied the cranial anatomy of the Proteus anguinus by x-ray tomography. In particular, they have imaged and segmented the sensory system of these animals

across three different stages of development: larval, juvenile and adult. They find that although eye development starts in the larval stage, it gradually reduces its size to the point of blindness, probably due to adaptation to a cave environment. In contrast, axolotls fully develop their eyes.

Overall the scientific data provided by the authors will certainly lead to further studies on the evolution of the salamander brain in the contest of cave adaptation. However, I do not recommend the publication of this manuscript without a major revision.

The authors should segment not only the brain, olfactory epithelium, residual eye and skull but also ear labyrinth and muscles across the three different stages of development. They should provide a side-by-side comparison in Figure 5 with the axolotl giving inputs on the different predatory habits by comparing the muscles and the jaws of the two species.

Response: Dear Reviewer,
Thank you for your comments and time spent on reviewing our manuscript. Following your suggestion, we added segmentations and 3D visualizations of the ear labyrinth for the all 5 samples in Figure 5. We also tried to segment the facial muscles. However, segmenting the facial muscles and making the binary mask for the larvae species could be misleading as the muscles only start to form at this stage of development, and as a result there is no clear border of the muscles. Thus, we didn't add the segmentations for larvae, however, we followed your advice for the adult specimens and added the data to Figure 5. We still believe that area of craniofacial muscles for larvae could be further examined by developmental biologists as we provide complete tomographic datasets.

The authors should also double check the scale bars of the top and middle image in Figure 1 as the dimensions of the animals are significantly different between the two stages of development.

Response: Thank you for this notice, we double checked the scalebars as we also slightly changed Figure 1 with semi-transparent heads in the first column.

It is not clear Figure 2 relates to the juvenile specimens from the text and this should be improved.

Response: Thank you for this note, we made it clearer by adding a sign "Juvenile Proteus anguinus" directly in the image. Also, we added this information to the Figure legend: "Accuracy validation of semi-automatic segmentations of X-ray microCT data in juvenile Proteus anguinus anguinus".

Furthermore the presentation of the synchrotron x-ray tomography of the axolotl brain in Figure 4 is redundant given the scope of the manuscript which is the study of Proteus anguinus sensory system. Either the authors present synchrotron images of the Proteus anguinus or they should omit the figure entirely as this is confusing to the readers.

Response: We thank reviewer also for this notice and we agree that it could be confusing for the readers, so we decided to change Figure 4 as you suggested and removed the synchrotron data. We also modified section "Perspective" in the text:
Perspectives: Cellular resolution
Using microCT scan with a conventional X-ray source, we obtained data of excellent quality which depict single cells in the cartilaginous elements (Figure 4). Despite the fact that the cells can be visually detected, their automatic segmentation and quantification is further challenging. The potential of X-ray microCT imaging with synchrotron sources for the study of 3D-cell distribution was demonstrated in our previous study on salamander limbs [12] and the potential for biomedical applications was shown before [19, 20]. The data with cellular resolution can be used as the input for the study of polarization of cells in the extracellular matrix in salamander limbs or for mathematical modelling of joint formation [12].

Finally, it is not clear the reason why the authors have stained the specimens with PTA and Iodine for several weeks as the contrasting protocol initially developed by Brian Metscher involves only overnight or few days of staining. Perhaps the authors could share the reason for this very long method.

Response: We thank the reviewer also for this point. Brian Metsher's papers usually refers to small-size animals. However, the adult Proteus and adult Ambystoma are much larger in comparison with specimens as mouse embryos on which Brian Metscher demonstrates some of his experiments. This is the reason why staining took significantly longer time. We add following clarification to the text to make it clearer also for the readers: "The adult Proteus anguinus and axolotl specimens were stained with 2% iodine (instead of PTA) in 90% methanol for six weeks to ensure that the contrasting agent would

penetrated to the entire sample, because iodine penetrates better than PTA.”

Reviewer #3: This Data Note showcases microCT datasets of the blind cave salamander Proteus anguinus, which is one of nature's curiosities, and also the Mexican axolotl Ambystoma mexicanum. Like other amphibians, Proteus anguinus begins eye development with the optic vesicles outgrowing from the diencephalon region of the developing brain and making contact with the surface ectoderm to initiate what is known as 'lens induction'. However, in Proteus the eyes soon regress after hatching. This adaptation to living in the dark is seen in other species, such as blind cave fish, and it is thought to be linked to the expression of key regulatory genes such as Pax6 and Shh (see Tian NM, Price DJ. Why cavefish are blind. Bioessays. 2005 Mar;27(3):235-8. doi: 10.1002/bies.20202). In addition, Proteus anguinus and the Mexican axolotl (Ambystoma mexicanum) have remarkable regenerative capabilities, and the axolotl is increasingly seen as a Model Organism for the study of regeneration (for example see Sanor LD, Flowers GP, Crews CM. Multiplex CRISPR/Cas screen in regenerating haploid limbs of chimeric Axolotls. Elife. 2020 Jan 28;9:e48511. doi: 10.7554/eLife.48511).

The larval and juvenile specimens are high quality cellular-resolution 3D models, and as with the previous GigaScience Data Note that was published by the authors (https://doi.org/10.1093/gigascience/giab012), the 3D models are high contrast by virtue of the specimens being stained with phosphotungstic acid prior to scanning. The adult specimens are of tissue-level resolution rather than cellular-level resolution, the difference in voxel size being directly linked to the physical dimensions of the sample. They are nevertheless of very high quality, and the authors further highlight that synchrotron X-ray microCT can produce superior quality images of juvenile and adult specimens (see Figure 4 in the manuscript).

A major strength of this newly submitted GigaScience Data Note is the careful delineation of key anatomical components in the scanned specimens. This involves considerable effort with every 3rd microCT slice being manually segmented and linear interpolation used to fill in the gaps. The 3D segmentations offer immense reuse potential, and enable researchers to further analyse key anatomical components - including brain, cartilage, bone, residual eyes, optic nerve, olfactory epithelium, ear labyrinth, and extraocular muscles - by morphometry and volumetric analysis.

Eye regression in Proteus is clearly of interest from an evolutionary and developmental biology (evo-devo) perspective. In addition, the anatomical detail provided in this study allows the authors to state that, "elongated and tube-shaped olfactory cavities in proteus likely emerge as another adaptation to the cave environment, where enhanced olfaction capabilities pose an advantage in the absence of visual signals". This novel and potentially fascinating adaptation may highlight a 'trade-off' between olfaction and vision during Proteus development. The authors allude to this in the manuscript, where they state, "when it comes to the adaptations in sensory organs, the 3D-analysis of the head revealed major differences in visual and olfactory systems of proteus and axolotl".

The authors additionally highlight the iconic status of Proteus, referred to in 'On the Origin of Species', where as the authors point out "Charles Darwin attributed the reduction of eyes wholly to their disuse in darkness." In addition, the authors' highlight that Proteus is classified as vulnerable, which means a species considered to be facing a high risk of extinction in the wild. The vulnerable status of this iconic species further increases the impact of this study.

In summary, the three stages of Proteus development and two stages of Ambystoma development are of interest from an evolutionary and developmental biology (evo-devo) perspective. Ambystoma is additionally of interest as a Model Organism for the study of regeneration.

I recommend this Data Note for publication in GigaScience.

Minor comment 1

In Figure 3 (larval and adult specimens of Proteus anguinus anguinus and Ambystoma mexicanum) and Figure 5 (adult Ambystoma mexicanum), the authors refer to the following segmentations:
-Brain
-Cartilage
-Bone
-Eyes / Residual Eyes

-Optic nerve
-Olfactory epithelium / bulbs
-Ear labyrinth
-Extraocular muscles (EOM)
-Craniofacial muscle

However, the STL files submitted to GigaDB only include the following:
-Brain
-Cartilage
-Bone
-Eyes / Residual eyes
-Olfactory epithelium
-Optic nerve (larval Ambystoma mexicanum)

Can the authors please submit the following 3D segmentation files to GigaDB?
-Optic nerve
-Ear labyrinth
-Extraocular muscles (EOM)
-Craniofacial muscle

Response: Dear Reviewer,
Thank you for your comments and time spent on reviewing our manuscript. We uploaded new STL files to GigaDB for all samples where the structure appears.

Minor comment 2

Can the authors provide the masks (binary image files) that were used to create the 3D surface reconstructions (STL format) from the volumetric DICOM image stacks? This is important for reproducibility, and for every segmentation this should include: 1) manually delineated image masks where every 3rd section was used according to the manuscript; 2) processed image masks where linear interpolation was used to fill in the gaps between manually delineated sections.

Response: We are thankful for this comment and we realize that for some purposes, masks can have different usage than STL files. Thus, we uploaded segmented masks to GigaDB for each segmented structure and add this information to “Availability of supporting data” part: For segmented structures, we also provide segmented masks as DICOM image stacks – one stack for each structure.
However, we did not upload the masks before interpolation as these data are only the preliminary step and contains artifacts caused by manual segmentation of the operator. This non-complete data are 34 GB, so we don’t see added value by adding them on the server and then someone can accidentally download these non-complete data and be confused. However, we are open to discussion regarding this topic.

Minor comment 3

Can the authors provide 3D surface reconstructions (STL format) of the whole specimens? This will provide the necessary context for enabling researchers to explore the relationship between surface anatomy and internal anatomy.

Response: Thank you for this suggestion. We uploaded STL format of the 3D surface for the all specimens to GigaDB.

Reviewer #4: Dear authors,
This is the first time I reviewed a manuscript for GigaScience. From my understanding the main aim of your study was to share a detailed 3D morphological dataset (including soft tissue) of the head of two salamander species. I guess that agrees well with the scope of GigaScience.
However I had some problems with accessing the science behind the presentation of the comparative morphology of the optic system of the cave dwelling paedomorphic olm Proteus anguinus and the well known lentic paedomorphic Axolotl Ambystoma mexicanum.

Response: Dear Reviewer,
Thank you for your comments and time spent on our manuscript, below we provide the answers for the points you raised:

(1) From my point of you should definitely present a much more detailed comparative analysis of your data, maybe in the results section, just expand it.

Response: We are thankful for this comment and we are aware that more analysis could bring greater insight to the topic. From this reason, we submitted the manuscript as the Data Note and not as the Research article, so our data and segmentations could be available for any researcher for further investigation.

(2) Then I would strongly suggest using the term "olm" instead of "proteus" (if you by all means want to apply a common name). Otherwise better apply the scientific name "Proteus anguinus".

Response: Thank you for this comment and we were discussing this matter with other co-authors whether to use "olm" or "proteus" or "Proteus anguinus" as the term differs among the literature. Based on your advice and on the text by Trontelj et al. (Recommendations for a consistent use of vernacular names for Proteus anguinus in English and Slovenian scientific texts, 2017), we decided to apply scientific name "Proteus anguinus" throughout the entire manuscript.

(3) You should be aware of that n = 2 Ambystoma mexicanum and n = 3 Proteus anguinus is not an exhaustive sample size at all.

Response: Thank you for this notice, we are aware that five samples are not an exhaustive sample size. Opposite to axolotl, proteus samples are often of an inadequate numerus due to the extreme rarity (e.g., only a few larvae were ever found in nature, difficult captive breeding), as well as because proteus is a protected species. In such circumstances, we believe that covering larval, juvenile and adult stages could bring interesting information. Towards with detailed contrasting, measuring and segmentation protocols, we believe that anyone could apply this procedure to specimens in their collection and compare with data provided by us.

(4) The use of English is OK in most parts of the manuscript but certainly not good enough to get published. I suggest you present the manuscript to a native speaker for a closer language check.

Response: We are thankful for this comment and for all annotations in the pdf version. We went carefully through the text and your comments. We also passed the manuscript to the native speaker for language check.

Minor comments I have made directly on the pdf version of the manuscript

But I hope my comments will aid in the revision

Bests

Alex Kupfer

Close
