# Supplementary material for: Living in darkness: Exploring adaptation of Proteus anguinus in 3 dimensions by X-ray imaging
Source: Gigascience. 2022 Apr 5;11:giac030. doi: 10.1093/gigascience/giac030 (PMC8982192; doi:10.1093/gigascience/giac030)
Supplement: giac030_Reviewer_1_Report_Original_Submission — Jia Jia -- 10/8/2021 Reviewed [file giac030_reviewer_1_report_original_submission.pdf]

Reviewer Report

Title: Living in darkness: Exploring adaptation of Proteus anguinus in 3D by X-ray imaging

Version: Original Submission **Date:** 10/8/2021

Reviewer name: Jia Jia

Reviewer Comments to Author:

The blind white olm (Proteus anguinus anguinus) are unique in many aspects of its biology, including geographic distribution, development and particularly its salient troglomorphism, which resulted from its morphological adaptation to underwater cave-dwelling lifestyle. As mentioned in this technical study, our knowledge on this particular salamander species was accumulated bit by bit over the past few hundred years. Now we are standing at a point where details of morphological and developmental changes of this taxon can be better revealed by non-destructive methods based on more readily available specimens raised in labs due to the advancement of high-resolution microCT scan and contrast enhancing techniques. Sharing of high-resolution CT images of endangered species that are significant to advance our understandings of certain scientific questions should always be encouraged.
This manuscript authored by TesaÅ™ovÃ¡ et al. provided a dataset of contrast-enhanced microCT images for the head region of three specimens (a larva, a juvenile and an adult) of Proteus anguinus anguinus and also images for two specimens (a larva and an adult) of Ambystoma mexicanum for the purpose of comparative studies. Besides many annotations I made in the word file as attached, here are the rest of my other concerns:
1) The comparison between the olm and the axolotl is reasonable considering both species are neotenic and one is cave dwelling and the other is surface dwelling, and therefore avoid introducing noises from changes created by metamorphosis; however, why not choose any neotenic species in the sister group genus of Proteus, Necturus, for comparison, considering their phylogenetic closeness and disparate lifestyles (Necturus is surface dwelling in slow-moving streams)?
2) After checking the dataset by loading dicom files into software VG Studio, it seems that the larval specimen of the olm has some shrink in the head region, which may be unavoidable, but I would appreciate if you can enrich the descriptions for the contrast-enhancing experimental procedures by providing the length of the rehydration process for each specimen.
3) Most of the datasets contain a complete head region except the one for the adult specimen of the axolotl, which has the posterior part of the hyobranchial apparatus missing. It is highly recommended to provide a more complete dataset for the axolotl adult specimen.
4) The dataset for the adult specimen of the olm can not be properly loaded in visualization softwares like VG Studio and Photoshop, because it has three damaged dicom files, i.e., "Proteus_anguinus_adult_0661.dcm", "Proteus_anguinus_adult_2023.dcm" and "Proteus_anguinus_adult_2349". The first image is 590 kb, and the latter two are 0 kb in size, in contrast to most other images in the same dataset which are 637 kb. It's also noticeable that 233 images ranging from "Proteus_anguinus_adult_2116.dcm" to "Proteus_anguinus_adult_2348.dcm" are 2317 kb, and can not be properly loaded into software with files of 637 kb.

Generally speaking, the manuscript is clearly written, and the dataset is easily accessible and well controlled. I look forward to its formal publication. Feel free to contact me (jia.jia@ucalgary.ca) directly if any of my comments are unclear.
Best wishes,
Jia
October 8, 2021

Level of Interest

Please indicate how interesting you found the manuscript: Choose an item.

Quality of Written English

Please indicate the quality of language in the manuscript: Choose an item.

Declaration of Competing Interests

Please complete a declaration of competing interests, considering the following questions:

- Have you in the past five years received reimbursements, fees, funding, or salary from an organisation that may in any way gain or lose financially from the publication of this manuscript, either now or in the future?
- Do you hold any stocks or shares in an organisation that may in any way gain or lose financially from the publication of this manuscript, either now or in the future?
- Do you hold or are you currently applying for any patents relating to the content of the manuscript?
- Have you received reimbursements, fees, funding, or salary from an organization that holds or has applied for patents relating to the content of the manuscript?
- Do you have any other financial competing interests?
- Do you have any non-financial competing interests in relation to this paper?

If you can answer no to all of the above, write 'I declare that I have no competing interests' below. If your reply is yes to any, please give details below.

I declare that I have no competing interests

I agree to the open peer review policy of the journal. I understand that my name will be included on my report to the authors and, if the manuscript is accepted for publication, my named report including any attachments I upload will be posted on the website along with the authors' responses. I agree for my report to be made available under an Open Access Creative Commons CC-BY license (http://creativecommons.org/licenses/by/4.0/). I understand that any comments which I do not wish to

be included in my named report can be included as confidential comments to the editors, which will not be published.

Choose an item.

To further support our reviewers, we have joined with Publons, where you can gain additional credit to further highlight your hard work (see: https://publons.com/journal/530/gigascience). On publication of this paper, your review will be automatically added to Publons, you can then choose whether or not to claim your Publons credit. I understand this statement.

Yes Choose an item.
