# Supplementary material for: Living in darkness: Exploring adaptation of Proteus anguinus in 3 dimensions by X-ray imaging
Source: Gigascience. 2022 Apr 5;11:giac030. doi: 10.1093/gigascience/giac030 (PMC8982192; doi:10.1093/gigascience/giac030)
Supplement: giac030_Reviewer_2_Report_Original_Submission — Claudio Polisseni -- 10/11/2021 Reviewed [file giac030_reviewer_2_report_original_submission.pdf]

Reviewer Report

Title: Living in darkness: Exploring adaptation of Proteus anguinus in 3D by X-ray imaging

Version: Original Submission **Date:** 10/11/2021

Reviewer name: Claudio Polisseni

Reviewer Comments to Author:

The authors have studied the cranial anatomy of the Proteus anguinus by x-ray tomography. In particular, they have imaged and segmented the sensory system of these animals across three different stages of development: larval, juvenile and adult. They find that although eye development starts in the larval stage, it gradually reduces its size to the point of blindness, probably due to adaptation to a cave environment. In contrast, axolotls fully develop their eyes.

Overall the scientific data provided by the authors will certainly lead to further studies on the evolution of the salamander brain in the contest of cave adaptation. However, I do not recommend the publication of this manuscript without a major revision.

The authors should segment not only the brain, olfactory epithelium, residual eye and skull but also ear labyrinth and muscles across the three different stages of development. They should provide a side-by-side comparison in Figure 5 with the axolotl giving inputs on the different predatory habits by comparing the muscles and the jaws of the two species.

The authors should also double check the scale bars of the top and middle image in Figure 1 as the dimensions of the animals are significantly different between the two stages of development.

It is not clear Figure 2 relates to the juvenile specimens from the text and this should be improved.

Furthermore the presentation of the synchrotron x-ray tomography of the axolotl brain in Figure 4 is redundant given the scope of the manuscript which is the study of Proteus anguinus sensory system. Either the authors present synchrotron images of the Proteus anguinus or they should omit the figure entirely as this is confusing to the readers.

Finally, it is not clear the reason why the authors have stained the specimens with PTA and Iodine for several weeks as the contrasting protocol initially developed by Brian Metscher involves only overnight or few days of staining. Perhaps the authors could share the reason for this very long method.

Level of Interest

Please indicate how interesting you found the manuscript: Choose an item.

Quality of Written English

Please indicate the quality of language in the manuscript: Choose an item.

Declaration of Competing Interests

Please complete a declaration of competing interests, considering the following questions:

- Have you in the past five years received reimbursements, fees, funding, or salary from an organisation that may in any way gain or lose financially from the publication of this manuscript, either now or in the future?
- Do you hold any stocks or shares in an organisation that may in any way gain or lose financially from the publication of this manuscript, either now or in the future?
- Do you hold or are you currently applying for any patents relating to the content of the manuscript?
- Have you received reimbursements, fees, funding, or salary from an organization that holds or has applied for patents relating to the content of the manuscript?
- Do you have any other financial competing interests?
- Do you have any non-financial competing interests in relation to this paper?

If you can answer no to all of the above, write 'I declare that I have no competing interests' below. If your reply is yes to any, please give details below.

No

I agree to the open peer review policy of the journal. I understand that my name will be included on my report to the authors and, if the manuscript is accepted for publication, my named report including any attachments I upload will be posted on the website along with the authors' responses. I agree for my report to be made available under an Open Access Creative Commons CC-BY license (http://creativecommons.org/licenses/by/4.0/). I understand that any comments which I do not wish to be included in my named report can be included as confidential comments to the editors, which will not be published.

Choose an item.

To further support our reviewers, we have joined with Publons, where you can gain additional credit to further highlight your hard work (see: https://publons.com/journal/530/gigascience). On publication of this paper, your review will be automatically added to Publons, you can then choose whether or not to claim your Publons credit. I understand this statement.

Yes Choose an item.
