# Supplementary material for: Living in darkness: Exploring adaptation of Proteus anguinus in 3 dimensions by X-ray imaging
Source: Gigascience. 2022 Apr 5;11:giac030. doi: 10.1093/gigascience/giac030 (PMC8982192; doi:10.1093/gigascience/giac030)
Supplement: giac030_Reviewer_2_Report_Revision_1 — Claudio Polisseni -- 2/7/2022 Reviewed [file giac030_reviewer_2_report_revision_1.pdf]

**Reviewer Report**

**Title: Living in darkness: Exploring adaptation of Proteus anguinus in 3D by X-ray imaging**

**Version: Revision 1** **Date:** 2/7/2022

**Reviewer name: Claudio Polisseni**

**Reviewer Comments to Author:**

Dear authors,
The manuscript has significantly improved. You have addressed all my major concerns and I therefore reccommend the pubblication in its current form. I am looking forward to sharing this paper and its further studies on the anatomical cave adaptation of Proteus anguinus.
Best wishes,
Claudio Polisseni

**Level of Interest**

Please indicate how interesting you found the manuscript: Choose an item.

**Quality of Written English**

Please indicate the quality of language in the manuscript: Choose an item.

**Declaration of Competing Interests**

Please complete a declaration of competing interests, considering the following questions:

- Have you in the past five years received reimbursements, fees, funding, or salary from an organisation that may in any way gain or lose financially from the publication of this manuscript, either now or in the future?
- Do you hold any stocks or shares in an organisation that may in any way gain or lose financially from the publication of this manuscript, either now or in the future?
- Do you hold or are you currently applying for any patents relating to the content of the manuscript?
- Have you received reimbursements, fees, funding, or salary from an organization that holds or has applied for patents relating to the content of the manuscript?
- Do you have any other financial competing interests?
- Do you have any non-financial competing interests in relation to this paper?

If you can answer no to all of the above, write 'I declare that I have no competing interests' below. If your reply is yes to any, please give details below.

I declare that I have no competing interests

I agree to the open peer review policy of the journal. I understand that my name will be included on my report to the authors and, if the manuscript is accepted for publication, my named report including any attachments I upload will be posted on the website along with the authors' responses. I agree for my report to be made available under an Open Access Creative Commons CC-BY license (http://creativecommons.org/licenses/by/4.0/). I understand that any comments which I do not wish to be included in my named report can be included as confidential comments to the editors, which will not be published.

Choose an item.

To further support our reviewers, we have joined with Publons, where you can gain additional credit to further highlight your hard work (see: https://publons.com/journal/530/gigascience). On publication of this paper, your review will be automatically added to Publons, you can then choose whether or not to claim your Publons credit. I understand this statement.

Yes Choose an item.
