# Supplementary material for: Living in darkness: Exploring adaptation of Proteus anguinus in 3 dimensions by X-ray imaging
Source: Gigascience. 2022 Apr 5;11:giac030. doi: 10.1093/gigascience/giac030 (PMC8982192; doi:10.1093/gigascience/giac030)
Supplement: giac030_Reviewer_3_Report_Original_Submission — Chris Armit -- 10/11/2021 Reviewed [file giac030_reviewer_3_report_original_submission.pdf]

Reviewer Report

Title: Living in darkness: Exploring adaptation of Proteus anguinus in 3D by X-ray imaging

Version: Original Submission **Date:** 10/11/2021

Reviewer name: Chris Armit

Reviewer Comments to Author:

This Data Note showcases microCT datasets of the blind cave salamander Proteus anguinus, which is one of nature's curiosities, and also the Mexican axolotl Ambystoma mexicanum. Like other amphibians, Proteus anguinus begins eye development with the optic vesicles outgrowing from the diencephalon region of the developing brain and making contact with the surface ectoderm to initiate what is known as 'lens induction'. However, in Proteus the eyes soon regress after hatching. This adaptation to living in the dark is seen in other species, such as blind cave fish, and it is thought to be linked to the expression of key regulatory genes such as Pax6 and Shh (see Tian NM, Price DJ. Why cavefish are blind. Bioessays. 2005 Mar;27(3):235-8. doi: 10.1002/bies.20202). In addition, Proteus anguinus and the Mexican axolotl (Ambystoma mexicanum) have remarkable regenerative capabilities, and the axolotl is increasingly seen as a Model Organism for the study of regeneration (for example see Sanor LD, Flowers GP, Crews CM. Multiplex CRISPR/Cas screen in regenerating haploid limbs of chimeric Axolotls. Elife. 2020 Jan 28;9:e48511. doi: 10.7554/eLife.48511).

The larval and juvenile specimens are high quality cellular-resolution 3D models, and as with the previous GigaScience Data Note that was published by the authors (https://doi.org/10.1093/gigascience/giab012), the 3D models are high contrast by virtue of the specimens being stained with phosphotungstic acid prior to scanning. The adult specimens are of tissue-level resolution rather than cellular-level resolution, the difference in voxel size being directly linked to the physical dimensions of the sample. They are nevertheless of very high quality, and the authors further highlight that synchrotron X-ray microCT can produce superior quality images of juvenile and adult specimens (see Figure 4 in the manuscript).

A major strength of this newly submitted GigaScience Data Note is the careful delineation of key anatomical components in the scanned specimens. This involves considerable effort with every 3rd microCT slice being manually segmented and linear interpolation used to fill in the gaps. The 3D segmentations offer immense reuse potential, and enable researchers to further analyse key anatomical components - including brain, cartilage, bone, residual eyes, optic nerve, olfactory epithelium, ear labyrinth, and extraocular muscles - by morphometry and volumetric analysis.

Eye regression in Proteus is clearly of interest from an evolutionary and developmental biology (evo-devo) perspective. In addition, the anatomical detail provided in this study allows the authors to state that, "elongated and tube-shaped olfactory cavities in proteus likely emerge as another adaptation to the cave environment, where enhanced olfaction capabilities pose an advantage in the absence of visual signals". This novel and potentially fascinating adaptation may highlight a 'trade-off' between olfaction and vision during Proteus development. The authors allude to this in the manuscript, where they state, "when it comes to the adaptations in sensory organs, the 3D-analysis of the head revealed major

differences in visual and olfactory systems of proteus and axolotl".
The authors additionally highlight the iconic status of Proteus, referred to in 'On the Origin of Species', where as the authors point out "Charles Darwin attributed the reduction of eyes wholly to their disuse in darkness." In addition, the authors' highlight that Proteus is classified as vulnerable, which means a species considered to be facing a high risk of extinction in the wild. The vulnerable status of this iconic species further increases the impact of this study.
In summary, the three stages of Proteus development and two stages of Ambystoma development are of interest from an evolutionary and developmental biology (evo-devo) perspective. Ambystoma is additionally of interest as a Model Organism for the study of regeneration.
I recommend this Data Note for publication in GigaScience.
Minor comment 1
In Figure 3 (larval and adult specimens of Proteus anguinus anguinus and Ambystoma mexicanum) and Figure 5 (adult Ambystoma mexicanum), the authors refer to the following segmentations:
-Brain
-Cartilage
-Bone
-Eyes / Residual Eyes
-Optic nerve
-Olfactory epithelium / bulbs
-Ear labyrinth
-Extraocular muscles (EOM)
-Craniofacial muscle
However, the STL files submitted to GigaDB only include the following:
-Brain
-Cartilage
-Bone
-Eyes / Residual eyes
-Olfactory epithelium
-Optic nerve (larval Ambystoma mexicanum)
Can the authors please submit the following 3D segmentation files to GigaDB?
-Optic nerve
-Ear labyrinth
-Extraocular muscles (EOM)
-Craniofacial muscle
Minor comment 2
Can the authors provide the masks (binary image files) that were used to create the 3D surface reconstructions (STL format) from the volumetric DICOM image stacks? This is important for reproducibility, and for every segmentation this should include: 1) manually delineated image masks where every 3rd section was used according to the manuscript; 2) processed image masks where linear interpolation was used to fill in the gaps between manually delineated sections.
Minor comment 3

Can the authors provide 3D surface reconstructions (STL format) of the whole specimens? This will provide the necessary context for enabling researchers to explore the relationship between surface anatomy and internal anatomy.

Level of Interest

Please indicate how interesting you found the manuscript: Choose an item.

Quality of Written English

Please indicate the quality of language in the manuscript: Choose an item.

Declaration of Competing Interests

Please complete a declaration of competing interests, considering the following questions:

- Have you in the past five years received reimbursements, fees, funding, or salary from an organisation that may in any way gain or lose financially from the publication of this manuscript, either now or in the future?
- Do you hold any stocks or shares in an organisation that may in any way gain or lose financially from the publication of this manuscript, either now or in the future?
- Do you hold or are you currently applying for any patents relating to the content of the manuscript?
- Have you received reimbursements, fees, funding, or salary from an organization that holds or has applied for patents relating to the content of the manuscript?
- Do you have any other financial competing interests?
- Do you have any non-financial competing interests in relation to this paper?

If you can answer no to all of the above, write 'I declare that I have no competing interests' below. If your reply is yes to any, please give details below.

I work for GigaScience where I perform checks on the quality of image data and the image analysis approach. My funding is not dependent on the outcome of this review, and I declare that I have no competing interests.

I agree to the open peer review policy of the journal. I understand that my name will be included on my report to the authors and, if the manuscript is accepted for publication, my named report including any attachments I upload will be posted on the website along with the authors' responses. I agree for my report to be made available under an Open Access Creative Commons CC-BY license (http://creativecommons.org/licenses/by/4.0/). I understand that any comments which I do not wish to

be included in my named report can be included as confidential comments to the editors, which will not be published.

Choose an item.

To further support our reviewers, we have joined with Publons, where you can gain additional credit to further highlight your hard work (see: https://publons.com/journal/530/gigascience). On publication of this paper, your review will be automatically added to Publons, you can then choose whether or not to claim your Publons credit. I understand this statement.

Yes Choose an item.
