# Supplementary material for: Living in darkness: Exploring adaptation of Proteus anguinus in 3 dimensions by X-ray imaging
Source: Gigascience. 2022 Apr 5;11:giac030. doi: 10.1093/gigascience/giac030 (PMC8982192; doi:10.1093/gigascience/giac030)
Supplement: giac030_Reviewer_3_Report_Revision_1 — Chris Armit -- 2/1/2022 Reviewed [file giac030_reviewer_3_report_revision_1.pdf]

**Reviewer Report**

**Title: Living in darkness: Exploring adaptation of Proteus anguinus in 3D by X-ray imaging**

**Version: Revision 1** **Date:** 2/1/2022

**Reviewer name: Chris Armit**

**Reviewer Comments to Author:**

This Data Note showcases microCT datasets of the blind cave salamander Proteus anguinus, which is one of nature's curiosities, and also the Mexican axolotl Ambystoma mexicanum. In this second review, I can confirm that the authors have addressed my comments, and in support of the manuscript have submitted the following surface-rendered 3D reconstructions to GigaDB.

Surface-rendered 3D reconstruction (STL format) of adult Ambystoma mexicanum - Complete surface 3D reconstruction

Surface-rendered 3D reconstruction (STL format) of adult Ambystoma mexicanum - 3D reconstruction of bone

Surface-rendered 3D reconstruction (STL format) of adult Ambystoma mexicanum - 3D reconstruction of brain

Surface-rendered 3D reconstruction (STL format) of adult Ambystoma mexicanum - 3D reconstruction of cartilage

Surface-rendered 3D reconstruction (STL format) of adult Ambystoma mexicanum - 3D reconstruction of craniofacial muscles

Surface-rendered 3D reconstruction (STL format) of adult Ambystoma mexicanum - 3D reconstruction of extraocular muscles

Surface-rendered 3D reconstruction (STL format) of adult Ambystoma mexicanum - 3D reconstruction of eyes

Surface-rendered 3D reconstruction (STL format) of adult Ambystoma mexicanum - 3D reconstruction of labyrinth

Surface-rendered 3D reconstruction (STL format) of adult Ambystoma mexicanum - 3D reconstruction of olfactory epithelium

Surface-rendered 3D reconstruction (STL format) of adult Ambystoma mexicanum - 3D reconstruction of optic nerve

Surface-rendered 3D reconstruction (STL format) of larval Ambystoma mexicanum - Complete surface 3D reconstruction

Surface-rendered 3D reconstruction (STL format) of larval Ambystoma mexicanum - 3D reconstruction of brain

Surface-rendered 3D reconstruction (STL format) of larval Ambystoma mexicanum - 3D reconstruction of cartilage

Surface-rendered 3D reconstruction (STL format) of larval Ambystoma mexicanum - 3D reconstruction of eyes

Surface-rendered 3D reconstruction (STL format) of larval Ambystoma mexicanum - 3D reconstruction of

labyrinth
Surface-rendered 3D reconstruction (STL format) of larval Ambystoma mexicanum - 3D reconstruction of olfactory epithelium
Surface-rendered 3D reconstruction (STL format) of larval Ambystoma mexicanum - 3D reconstruction of optic nerve
Surface-rendered 3D reconstruction (STL format) of adult Proteus anguinus - Complete surface 3D reconstruction
Surface-rendered 3D reconstruction (STL format) of adult Proteus anguinus - 3D reconstruction of bone
Surface-rendered 3D reconstruction (STL format) of adult Proteus anguinus - 3D reconstruction of brain
Surface-rendered 3D reconstruction (STL format) of adult Proteus anguinus - 3D reconstruction of cartilage
Surface-rendered 3D reconstruction (STL format) of adult Proteus anguinus - 3D reconstruction of craniofacial muscles
Surface-rendered 3D reconstruction (STL format) of adult Proteus anguinus - 3D reconstruction of labyrinth
Surface-rendered 3D reconstruction (STL format) of adult Proteus anguinus - 3D reconstruction of olfactory epithelium
Surface-rendered 3D reconstruction (STL format) of juvenile Proteus anguinus - Complete surface 3D reconstruction
Surface-rendered 3D reconstruction (STL format) of juvenile Proteus anguinus - 3D reconstruction of bone
Surface-rendered 3D reconstruction (STL format) of juvenile Proteus anguinus - 3D reconstruction of brain
Surface-rendered 3D reconstruction (STL format) of juvenile Proteus anguinus - 3D reconstruction of cartilage
Surface-rendered 3D reconstruction (STL format) of juvenile Proteus anguinus - 3D reconstruction of eyes
Surface-rendered 3D reconstruction (STL format) of juvenile Proteus anguinus - 3D reconstruction of labyrinth
Surface-rendered 3D reconstruction (STL format) of juvenile Proteus anguinus - 3D reconstruction of olfactory epithelium
Surface-rendered 3D reconstruction (STL format) of larval Proteus anguinus - Complete surface 3D reconstruction
Surface-rendered 3D reconstruction (STL format) of larval Proteus anguinus - 3D reconstruction of brain
Surface-rendered 3D reconstruction (STL format) of larval Proteus anguinus - 3D reconstruction of cartilage
Surface-rendered 3D reconstruction (STL format) of larval Proteus anguinus - 3D reconstruction of eyes
Surface-rendered 3D reconstruction (STL format) of larval Proteus anguinus - 3D reconstruction of labyrinth
Surface-rendered 3D reconstruction (STL format) of larval Proteus anguinus - 3D reconstruction of olfactory epithelium
In addition, in response to my request I can confirm that the authors have additionally submitted to

GigaDB the masks (DICOM format) used to generate the 3D surface reconstructions. These masks represent the processed image masks after linear interpolation, and are an important addition to this imaging dataset.
I can additionally confirm that the authors have addressed the major comment made by Reviewer 2 by submitting the aforementioned segmentations and 3D visualisations of the brain (adult specimens), olfactory epithelium (adult specimens), eyes (larval and adult specimens of Ambystoma mexicanum, larval and juvenile specimens of Proteus anguinus), ear labyrinth (all specimens) and craniofacial muscles (adult specimens). In their response to Reviewer 2, the authors have provided what I consider to be a reasonable explanation as to why segmentation of craniofacial muscles was not accomplished at earlier stages, which is that there is inherent ambiguity as to the boundary of muscle tissue in the craniofacial region at these earlier (larval, juvenile) stages. In addition, 3D segmentations of residual eye are not provided for adult Proteus anguinus, but I understand that this reflects this anatomical component - which is a vestigial tissue in the juvenile - being absent in the adult. This is my understanding from the segmentations shown in Figures 1 and 2, but it would be helpful if the authors could clarify as to whether this is indeed the case.
I further confirm that the authors have addressed the comments of Reviewer 4, with some notable improvements in the language in the revised manuscript, and specifically with the species name Proteus anguinus now being used throughout the manuscript. However, I do note that some incidences of the species name "Proteus anguinus" read as "Proteus anguinus anguinus", for example in the Figure legends. I request that the authors correct any type errors relating to species names, and that they are consistent with species names in the main body of the text and the Figure legends.
Furthermore, Reviewer 1 noted corrupt files in one of the DICOM image stacks. To address this issue, the authors have now updated this DICOM directory. However, to ensure that there are not any additional corrupt files that may be harboring within this imaging dataset, I request that the authors liaise with the GigaScience Database team and provide MD5 checksums for all image data files that were submitted to GigaDB. This will ensure that file integrity was maintained during file transfer.

**Level of Interest**

Please indicate how interesting you found the manuscript: Choose an item.

**Quality of Written English**

Please indicate the quality of language in the manuscript: Choose an item.

**Declaration of Competing Interests**

Please complete a declaration of competing interests, considering the following questions:

- Have you in the past five years received reimbursements, fees, funding, or salary from an organisation that may in any way gain or lose financially from the publication of this manuscript, either now or in the future?
- Do you hold any stocks or shares in an organisation that may in any way gain or lose financially from the publication of this manuscript, either now or in the future?
- Do you hold or are you currently applying for any patents relating to the content of the manuscript?
- Have you received reimbursements, fees, funding, or salary from an organization that holds or has applied for patents relating to the content of the manuscript?
- Do you have any other financial competing interests?
- Do you have any non-financial competing interests in relation to this paper?

If you can answer no to all of the above, write 'I declare that I have no competing interests' below. If your reply is yes to any, please give details below.

I work for GigaScience where I perform checks on the quality of image data and the image analysis approach. My funding is not dependent on the outcome of this review, and I declare that I have no competing interests.

I agree to the open peer review policy of the journal. I understand that my name will be included on my report to the authors and, if the manuscript is accepted for publication, my named report including any attachments I upload will be posted on the website along with the authors' responses. I agree for my report to be made available under an Open Access Creative Commons CC-BY license (http://creativecommons.org/licenses/by/4.0/). I understand that any comments which I do not wish to be included in my named report can be included as confidential comments to the editors, which will not be published.

Choose an item.

To further support our reviewers, we have joined with Publons, where you can gain additional credit to further highlight your hard work (see: https://publons.com/journal/530/gigascience). On publication of this paper, your review will be automatically added to Publons, you can then choose whether or not to claim your Publons credit. I understand this statement.

Yes Choose an item.
