# Supplementary material for: Living in darkness: Exploring adaptation of Proteus anguinus in 3 dimensions by X-ray imaging
Source: Gigascience. 2022 Apr 5;11:giac030. doi: 10.1093/gigascience/giac030 (PMC8982192; doi:10.1093/gigascience/giac030)
Supplement: giac030_Reviewer_4_Report_Original_Submission — Alexander Kupfer -- 10/19/2021 Reviewed [file giac030_reviewer_4_report_original_submission.pdf]

Reviewer Report

Title: Living in darkness: Exploring adaptation of Proteus anguinus in 3D by X-ray imaging

Version: Original Submission **Date:** 10/19/2021

Reviewer name: Alexander Kupfer

Reviewer Comments to Author:

Dear authors,
This is the first time I reviewed a manuscript for GigaScience. From my understanding the main aim of your study was to share a detailed 3D morphological dataset (including soft tissue) of the head of two salamander species. I guess that agrees well with the scope of GigaScience.
However I had some problems with accessing the science behind the presentation of the comparative morphology of the optic system of the cave dwelling paedomorphic olm Proteus anguinus and the well known lentic paedomorphic Axolotl Ambystoma mexicanum.
(1) From my point of you should definitely present a much more detailed comparative analysis of your data, maybe in the results section, just expand it.
(2) Then I would strongly suggest using the term "olm" instead of "proteus" (if you by all means want to apply a common name). Otherwise better apply the scientific name "Proteus anguinus".
(3) You should be aware of that n = 2 Ambystoma mexicanum and n = 3 Proteus anguinus is not an exhaustive sample size at all.
(4) The use of English is OK in most parts of the manuscript but certainly not good enough to get published. I suggest you present the manuscript to a native speaker for a closer language check.
Minor comments I have made directly on the pdf version of the manuscript
But I hope my comments will aid in the revision
Bests
Alex Kupfer

Level of Interest

Please indicate how interesting you found the manuscript: Choose an item.

Quality of Written English

Please indicate the quality of language in the manuscript: Choose an item.

Declaration of Competing Interests

Please complete a declaration of competing interests, considering the following questions:

- Have you in the past five years received reimbursements, fees, funding, or salary from an organisation that may in any way gain or lose financially from the publication of this manuscript, either now or in the future?
- Do you hold any stocks or shares in an organisation that may in any way gain or lose financially from the publication of this manuscript, either now or in the future?
- Do you hold or are you currently applying for any patents relating to the content of the manuscript?
- Have you received reimbursements, fees, funding, or salary from an organization that holds or has applied for patents relating to the content of the manuscript?
- Do you have any other financial competing interests?
- Do you have any non-financial competing interests in relation to this paper?

If you can answer no to all of the above, write 'I declare that I have no competing interests' below. If your reply is yes to any, please give details below.

I herewith declare that I have no known competing financial interests or personal relationships that could have appeared to influence my review work of this manuscript.

I agree to the open peer review policy of the journal. I understand that my name will be included on my report to the authors and, if the manuscript is accepted for publication, my named report including any attachments I upload will be posted on the website along with the authors' responses. I agree for my report to be made available under an Open Access Creative Commons CC-BY license (http://creativecommons.org/licenses/by/4.0/). I understand that any comments which I do not wish to be included in my named report can be included as confidential comments to the editors, which will not be published.

Choose an item.

To further support our reviewers, we have joined with Publons, where you can gain additional credit to further highlight your hard work (see: https://publons.com/journal/530/gigascience). On publication of this paper, your review will be automatically added to Publons, you can then choose whether or not to claim your Publons credit. I understand this statement.

Yes Choose an item.
